# The Targeted Pesticides as Acetylcholinesterase Inhibitors: Comprehensive Cross-Organism Molecular Modelling Studies Performed to Anticipate the Pharmacology of Harmfulness to Humans In Vitro

**DOI:** 10.3390/molecules23092192

**Published:** 2018-08-30

**Authors:** Milan Mladenović, Biljana B. Arsić, Nevena Stanković, Nezrina Mihović, Rino Ragno, Andrew Regan, Jelena S. Milićević, Tatjana M. Trtić-Petrović, Ružica Micić

**Affiliations:** 1 Kragujevac Center for Computational Biochemistry, Faculty of Science, University of Kragujevac, Radoja Domanovića 12, P.O. Box 60, 34000 Kragujevac, Serbia; nevena.stankovic@pmf.kg.ac.rs (N.S.); nezrina.mihovic@pmf.kg.ac.rs (N.M.); 2 Department of Mathematics, Faculty of Sciences and Mathematics, University of Niš, Višegradska 33, 18000 Niš, Serbia; biljana.arsic@pmf.edu.rs; 3 Division of Pharmacy and Optometry, University of Manchester, Oxford Road, Manchester M13 9PT, UK; 4 Rome Center for Molecular Design, Department of Drug Chemistry and Technologies, Faculty of Pharmacy and Medicine, Sapienza Rome University, P.le A. Moro 5, 00185 Rome, Italy; rino.ragno@uniroma1.it; 5 Alchemical Dynamics srl, 00125 Rome, Italy; 6 School of Chemistry, University of Manchester, Oxford Road, Manchester M13 9PL, UK; Andrew.Regan@manchester.ac.uk; 7 Vinča Institute of Nuclear Sciences, University of Belgrade, P.O. Box 522, 11001 Belgrade, Serbia; jdjordjevic@vin.bg.ac.rs (J.S.M.); ttrtic@vinca.rs (T.M.T.-P.); 8 Faculty of Sciences and Mathematics, University of Priština, Lole Ribara 29, 38220 Kosovska Mitrovica, Serbia; ruzica.micic@pr.ac.rs

**Keywords:** pesticides, AChE, conformational analysis, QSAR, molecular docking, molecular dynamics, quantum-chemical studies, concentration-dependent kinetic studies

## Abstract

Commercially available pesticides were examined as *Mus musculus* and *Homo sapiens* acetylcholinesterase (*m*AChE and *h*AChE) inhibitors by means of ligand-based (LB) and structure-based (SB) in silico approaches. Initially, the crystal structures of simazine, monocrotophos, dimethoate, and acetamiprid were reproduced using various force fields. Subsequently, LB alignment rules were assessed and applied to determine the inter synaptic conformations of atrazine, propazine, carbofuran, carbaryl, tebufenozide, imidacloprid, diuron, monuron, and linuron. Afterwards, molecular docking and dynamics SB studies were performed on either *m*AChE or *h*AChE, to predict the listed pesticides’ binding modes. Calculated energies of global minima (*E*_glob_min_) and free energies of binding (∆*G*_binding_) were correlated with the pesticides’ acute toxicities (i.e*.*, the LD_50_ values) against mice, as well to generate the model that could predict the LD_50_s against humans. Although for most of the pesticides the low *E*_glob_min_ correlates with the high acute toxicity, it is the ∆*G*_binding_ that conditions the LD_50_ values for all the evaluated pesticides. Derived *pLD*_50_ = *f*(∆*G*_binding_) *m*AChE model may predict the *pLD*_50_ against *h*AChE, too. The *h*AChE inhibition by atrazine, propazine, and simazine (the most toxic pesticides) was elucidated by SB quantum mechanics (QM) DFT mechanistic and concentration-dependent kinetic studies, enriching the knowledge for design of less toxic pesticides.

## 1. Introduction

Pesticides [1] are either chemical or biological agents, widely used in agriculture [2], that deter, incapacitate, kill, or otherwise discourage pests. Their inappropriate administration leads to contaminated food while intentional release pollutes the environment, especially the soil, groundwater and natural waterways [3]. The uptake of contaminated food and water is potentially toxic to humans and other species. Pesticides mostly, exert toxicity by inhibiting the enzyme acetylcholinesterase (AChE) [4] which degrades acetylcholine (ACh), an essential neurotransmitter in the central nervous system (CNS) of insects, rodents, and humans [5]. AChE competitive inhibitors interrupt the physiology of autonomic ganglia, as well as, neuromuscular, parasympathetic and sympathetic effector junctions [6], controlled by ACh. However, little is known about the pharmacology of pesticides acting as AChE inhibitors. This report considers the pharmacology of common agricultural pesticides used in particular in the Western Balkans region [7,8]: atrazine, simazine, propazine, carbofuran, monocrotophos, dimethoate, carbaryl, tebufenozide, imidacloprid, acetamiprid, diuron, monuron, and linuron (Table 1). Despite their widespread usage, experimental crystal structures deposited at Cambridge Crystallographic Data Centre (CCDC) are available only for simazine [9], monocrotophos [10], dimethoate [11], and acetamiprid [12], highlighting the poor availability of either ligand-based (CCDC deposition) or structure-based (Protein Data Bank (PDB) deposition) information about these compounds. 

Triazines, such as atrazine, simazine, and propazine [13], are used as herbicides in crabgrass supression. Carbamates (carbofuran and carbaryl) are used against potato beetle; carbofuran was found extremely toxic for humans [14]. Organophosphate pesticides (monocrotophos and dimethoate) are used as insecticides [15]. Dimethoate is widely used to kill insects and as an insecticide for crops, vegetables, orchards, as well as for residential purposes [16]. Tebufenozide (a dialkylhydrazine pesticide) is used for plant protection [17]. Imidacloprid and acetamiprid, as neonicotinoids, are prescribed for the control of sucking insect pests (aphids, whiteflies, plant-hoppers, and thrips) and a number of coleopteran pests [17,18]. Diuron (a methylurea pesticide) is used to protect fruit, cotton, sugar cane and wheat [13]. Linuron and monuron (phenylurea pesticides) are plant protection agents [19]. Lethal doses for house and field mouse *Mus musculus*, for all of the above-mentioned pesticides, are known (Table 1) [20,21,22,23,24,25,26,27,28,29,30,31]. Lethal doses for *Homo sapiens* were calculated (Table 1) according to the recommendations of Reagan-Shaw et al. [32], using Equation (1):

(1) $$HED=animal\;dose\;\frac{animal\;K_{m},}{human\;K_{m}}$$

where *HED* is human equivalent dose in mg/kg of body weight, animal dose is used dosage for the specific model organism in mg/kg of body weight, animal *K_m_* factor is equal to 3, human *K_m_* factor is equal to 37.

The overexposure to pesticides results in the AChE inhibition at brain and nervous system nerve endings, as well as with other types of AChE found in the blood [33]; as a consequence, the ACh concentration increases its effects. Although, the AChE inhibition by pesticides listed in Table 1 is firmly established and the lethal doses are known, their behaviour in the inter-synaptic space and their interaction within the AChE active site, are still poorly investigated. Bearing in mind that different ACh conformations can trigger different receptors (nicotinics and muscarinics, respectively), conformational analysis (CA, ligand-based (LB) approach) could be considered as a computational tool to predict pesticides behaviour before their interaction with AChE [34]. Due to the lack of crystal structures of any pesticides co-crystallized with AChE, CA can be used to gather pesticide conformations that are, upon the interaction with AChE, going to be transformed into the bioactive ones [35]. 

**Figure 1:**
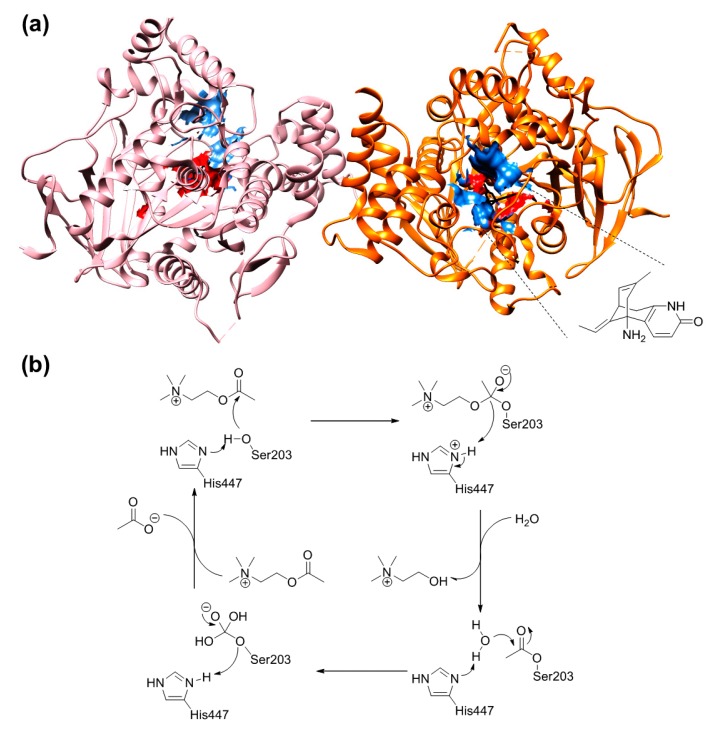
The crystal structure of *h*AChE (PDB ID: 4EY5) in complex with huperzine A (**a**). Subunit A is depicted in pink ribbons whereas the B subunit is coloured in orange. Huperzine A is presented in black and its structure is highlighted with dashed lines. The anionic sub-site is described by blue spheres, while the esterase sub-area is encircled by red spheres. For the sake of clarity, hydrogen atoms were omitted. The graphic was generated using the UCSF Chimera software (Resource for Biocomputing, Visualization, and Informatics (RBVI), University of California, San Francisco, CA, USA). Biochemical mechanism of ACh hydrolysis by AChE (**b**).

For either mouse or humans as a model organism, AChE is a homodimer (Figure 1a), formed of two independent units, comprising 614 amino acids [36]. The AChE active site comprises the aromatic anionic domain, which accommodates the ACh positive quaternary amine, and the esterase catalytic domain, containing the catalytic triad Ser203, His447, and Glu334 with the purpose of hydrolysing the ACh to acetate and choline. The ester hydrolysis reaction leads to the formation of an acetylated-enzyme. Then, the acetyl-enzyme undergoes nucleophilic attack by a water molecule, assisted by the His447, freeing the acetic acid, likely as an acetate ion, and regenerating the free enzyme (Figure 1b) [33].

Given that the pharmacology of the majority of widely used pesticides (Table 1) is still poorly understood, the focus of this study was to provide the LB or SB cheminformatics-based guidelines for several important tasks, and unresolved questions: (1) how to define the pesticides’ structures in aqueous solution prior to interaction with AChE? (2) how to define the pesticides’ bioactive conformations upon the interaction with AChE? Other important assignments of the LB or SB protocols applied herein were to provide answers to several important questions: (1) is there any possibility to anticipate the pesticides acute toxicity from pre-bound conformations, before the actual interaction with AChE? (2) can the pesticides’ acute toxicity be quantified from the bound conformations, upon the actual interaction with AChE? To the best of our knowledge, there are no previous reports on either LB or SB efforts to predict the bioactive conformations of investigated pesticides (Table 1) for both mouse and human AChEs, as well as to enumerate their acute toxicities by means of free energy of formation (*E*_glob_min_) in the aqueous solution and/or free energy of binding (∆*G*_binding_) to AChE. All the cheminformatics findings were externally validated on the series of other commonly used pesticides (Table 2), also known as AChE inhibitors (i.e., the test set). 

The test set was compiled of organophosphates, carbamates and structurally non-classified pesticides. Regarding organophosphates, chlorpyrifos [37], DDVP [14], fenitrothion [38], azinphos-methyl [39], naled (dibrom) [40], TCVP [41], parathion [39], methyl parathion [42], diazinon [43], phosmet [14], azamethiphos [44], and terbufos [45], respectively, are used as insecticides. Moreover, glyphosate is a broad-spectrum systemic herbicide and crop desiccant [46], malathion is mostly used for mosquito eradication [47], parathion is used as an acaricide. Phosmet is mainly used on apple trees to control codling moth and on a numerous fruit crops, ornamentals, and wines for the control of aphids, suckers, mites, and fruit flies. Terbufos is a nematicide used on corn, sugar beets and grain sorghum, and it is extremely toxic to birds. On the other hand, carbamate pesticides, such as methomyl and methiocarb are used against insects; oxamyl is an extremely toxic pesticide to humans, fish, and birds [48,49,50], whereas methiocarb is additionally used as a bird repellent, acaricide and molluscicide. Among the structurally non-classified pesticides, 2,4-D is widely used as a herbicide, which mimics the action of the plant growth hormone auxin, resulting in the uncontrolled growth and eventual death in susceptible plants [14]; DDT is originally developed as an insecticide, used to control malaria and typhus, and as a contact poison against several arthropods [51]; DEET is the most common active ingredient in insect repellents for protection against mosquitoes, ticks, fleas, chiggers, leeches and many biting insects [52].

Among all of the listed pesticides (training and test set compounds), the pharmacology of organophosphorus (OP), compounds as *h*AChE inhibitors, is by far the most investigated; it involves the phosphorylation of catalytic Ser203 and the formation of stable inactive phosphyl-AChE adducts [36]. Moreover, methylcarbamate (MC) insecticides like carbofuran and similar compounds, occupy the catalytic triad area and act as reversible competitive inhibitors of ACh [73], elucidated by molecular docking (SB) studies on *Nephotettix cincticeps* AChE. Docking studies were also performed for imidacloprid analogues [74].

Therefore, to enrich the information related to the pharmacology of commonly used commercial pesticides, the targets of herein study, their mechanisms of action were either confirmed or nominated by means of reversible and reversible-like (for organophosphorus pesticides only, docking performed to obtain non-covalent conformation that is going to be transformed to the covalent one) cross-docking, as well as by single step molecular dynamics studies on *Mus musculus* and *Homo sapiens* AChE level. Alongside with the prediction of free energy of binding, generated bioactive conformations, were the starting points for the definition of the mechanism of action of 2-chloro-1,3,5-triazine-based pesticides (atrazine, simazine, and propazine, respectively), as the most toxic ones. DFT quantum mechanics mechanistic studies were supposed to shed further light on pesticide-AChE interactions for the purpose of designing pesticides with lower acute toxicity profiles.

## 2. Results and Discussion

### 2.1. The Conformational Analysis of Pesticides in Aqueous Solution

Conformational analysis (CA) studies are herein reported for simazine [9], monocrotophos [10], dimethoate [11], and acetamiprid [12] (Table 3), as pesticides with known crystal structures, as well as for atrazine and carbofuran, as pesticides that exert the highest acute toxicity (Table 4). To avoid redundancy, applied studies to the other pesticides are reported as Supplementary Material. The crystal structures of simazine [9], monocrotophos [10], dimethoate [11], and acetamiprid [12] (Table 3, Tables S1–S3) were used as starting conformational geometries for the CA. For pesticides without experimental data (Table 4, Tables S1–S3), starting conformations were built from scratch and energy minimized. For the sake of clarity, pesticides were divided into four groups based on their common structural patterns: 2-chloro-1,3,5-triazine derivatives: simazine, atrazine, and propazine (Table 3 and Table 4, Table S1, respectively); amide derivatives: monocrotophos, dimethoate, and carbofuran (Table 3 and Table 4, Table S2, respectively), carbaryl and tebufenozide (Table S2); 6-chloropyridine-3-yl)methanamine derivates: acetamiprid (Table 3, Table S3) and imidacloprid (Table S3); 1-(4-chlorophenyl)-3-methylurea condensates: diuron, monuron, and linuron (Table S3). 

CA in explicit solvent may be used to predict the single pesticide stability prior to AChE binding. Herein, CAs were tentatively carried out by using five different force fields (FFs): MM3, AMBER94, MMFF, MMFFs, and OPLSAA [35]. Either for crystallized pesticides or pesticides with unknown experimental structures, the procedure was divided into the following steps: (1) conformational analysis; (2) determination of energy of the global minimum (*E*_glob_min_), and (3) the LB superposition of structures to define the alignment accuracy. All steps were performed in order to obtain the best performing FF, either by means of capability to predict the free energy of global minimum or to superimpose molecules. The higher goal of CA was to answer the question: How to consider the FF of choice while treating pesticides: by predicted *E*_glob_min_ values or by alignment capability?

CA results for simazine [9], monocrotophos [10], dimethoate [11], and acetamiprid [12] are reported in Table 3. MM3 and AMBER94 could not be applied to all of the crystallized pesticides as the needed parameters for the optimization were not available. Possible workaround could be the utilization of Antechamber suite [75], by which the generic AMBER parameters could be calculated, but then a different protocol would have been developed instead of the one based on the well-known accredited Schrödinger software [76]. MMFF showed to be the only FF containing the parameterizations for all of the compounds; MMFFs failed in the optimization of simazine whereas OPLSAA was not applicable to monocrotophos and acetamiprid. 

The absolute values of *E*_glob_min_ are not necessarily good measures of FF quality [77]. FFs and are usually parameterized to give good values of relative energies among different conformations of the same molecule [77], so a lower value of energy for a global minimum does not necessarily mean that a given FF would perform better [77]. The energy differences between the conformations are simple reliable indicators of conformational analysis algorithm ability than the absolute energy values [77]. 

Therefore, the evaluation of conformation reproducibility was achieved by means of calculated minima root mean square deviation (RMSD) values [78] upon conformations’ superimposition (Table 3 and Table 4, Tables S1–S3). Comparison of the experimentally available crystal structures conformations with those obtained by CA revealed, a high level of performance by MMFF (displaying RMSD values lower than 1 Å for all of the four considered pesticides) [78]. That was somehow expected, given that MMFF-based optimization was applicable to all of the listed pesticides. Regarding the MMFFs and OPLSAA, these FFs only succeeded in reproducing the crystal structure of acetamiprid. 

### 2.2. The Prediction of Pesticides Structures in Aqueous Solution

The above assessed CA procedure was further applied to the training set pesticides with an unknown crystal structures. The results of optimization for the pesticides with the highest acute toxicity (i.e., the lowest LD_50_ values), atrazine and carbofuran, using the best performing FFs, are reported in Table 4, while the comparison of all utilized FFs for atrazine, carbofuran, and the remaining compounds are presented in Tables S1–S3. As expected, AMBER94 FF, as implemented in Schrödinger suite [76] was not applicable at all, MM3, MMFF and MMFFs FFs gave similar global minima for the majority of the listed pesticides, while OPLSAA was not applicable for all compounds. A significant conformational alignment accuracy (RMSD < 1 Å) was achieved for atrazine (MM3/MMFF, MM3/MMFFs, and MMFF/MMFFs combinations, Table 2, Table S1) and carbofuran, (MMFF/MMFFs, MMFF/OPLSAA, and MMFFs/OPLSAA combinations, Table 3, Table S2). Summarizing the CA results, conclusion can be made, despite the limited number of training set compounds, MMFF is universally applicable FF for either *E*_glob_min_ calculation or RMSD-based alignment accuracy assessment and can be used in any further study, describing the pesticides conformational analysis.

### 2.3. The Prediction of Externally Evaluated Pesticides Structures in Aqueous Solution

MMFF was thereafter used to predict the global minimum conformation of test set pesticides (Table 2) as well. The results of the optimisation are presented as Supplementary Materials (Table S4). Global minimum conformations were generated for by the means of the identical CA setup. Generated conformations were then used for the purposes of training set results external validation. 

### 2.4. The Pesticides Acute Toxicity Anticipation through the Pre-Bound Conformations

The lowest energy conformers of the 2-chloro-1,3,5-triazine group were of high stability in the aquous solution, according to the values obtained by MMFF (Table 3 and Table 4, Table S1). Being chemically stable, pesticides may in perspective saturate the AChE active site and low values of global minimum (*E*_glob_min_) represent the indicators of high acute toxicity (i.e., the low LD_50_ values) against *Mus musculus*. For either atrazine, propazine, or simazine, low *E*_glob_min_ values are indeed the precondition of high acute toxicity (Table 1: LD_50_ values equal to 0.85, 3.18, and 5 mg/kg of body weight, respectively, Table 3 and Table 4, Table S1: *E*_glob_min_ values equal to −1007.32, −1022.18, and −992.47 kJ/mol, respectively). For much of the lasting pesticides (monocrotophos and dimethoate: Table 1 vs. Table 3 and Table S2; carbaryl and tebufenozide: Table 1 vs. Table S2; imidacloprid: Table 1 vs. Table 3 and Table S3, and acetamiprid: Table 1 vs. Table S3), it appeared that high to medium acute toxicities mostly correlate with low to medium *E*_glob_min_ values.

The above paradigm was confirmed for carbaryl and tebufenozide, where the high *E*_glob_min_ values are a precondition for low acute toxicity (Table 1: LD_50_ values equal to 100 and 5000 mg/kg of body weight, respectively, Table S2: *E*_glob_min_ values equal to 20.43 and 467.38 kJ/mol, respectively), as well as for monocrotophos and dimethoate, whose medium LD_50_ values (Table 1: LD_50_ equal to 14 and 60 mg/kg of body weight, respectively) are associated with medium *E*_glob_min_ values (Table 3: *E*_glob_min_ values equal to −307.36 and −383.68 kJ/mol, respectively). On the other hand, the high acute toxicity of carbofuran against *Mus musculus* (Table 1: LD_50_ = 2 mg/kg of body weight) disagreed with calculated high *E*_glob_min_ values (Table 4: *E*_glob_min_ value equal to 20.30 kJ/mol). Interestingly, a thermodynamic parameter calculated for carbofuran suggests some instability in aqueous solution; according to the previous observations, a high dosage of pesticide would be required to induce mortality in *Mus musculus*. Therefore, other parameters need to be considered, to explain why carbofuran acts as an outlier.

Among the last group of pesticides, acetamiprid (Table 1 vs. Table 3 and Table S3) and imidacloprid (Table 1 vs. Table S3) also follow the archetype that relatively low *E*_glob_min_ values are a precondition for medium LD_50_ dosages. Linuron, however, behaves oppositely to 2-chloro-1,3,5-triazines group inasmuch as with its low *E*_glob_min_ values there is a high LD_50_ value (Table 1: LD_50_ = 2400 mg/kg of body weight, Table S3: *E*_glob_min_ value equal to −992.47 kJ/mol). Linuron’s structural analogues diuron and monuron, despite the fact they are slightly less stable (higher *E*_glob_min_ values, Table S3: *E*_glob_min_ values equal to −142.96 and −197.63 kJ/mol, respectively) need to be administered at high dosages to induce mice mortality (Table 1, LD_50_ = 500 and 1700 mg/kg of body weight, respectively). 

In order to enumerate the *Mus musculus LD*_50_/*E*_glob_min_ interconnection, simple linear regression (as implemented in LibreOffice Calc) was analysed considering *pLD_5_*_0_ (calculated by converting LD_50_ from mg/kg of body weight to −log(mol/kg of body weight)), as dependent variable, and *E*_glob_min_ values as calculated by MMFF (Table S5), as descriptive independent variables. The use of all the data (model 0) did not lead to a significant statistical regression. However, the exclusion of carbofuran and linuron as highlighted outliers (those with the worse p*LD*_50_ fitted values), led to a monoparametric QSAR model 1 (Equation (2)):

(2) $$\;pLD_{50\;}=-0.0021E_{\mathrm{glob}\_\min}-2.82\;\left(r^{2}=0.78\right),$$

**Figure 2:**
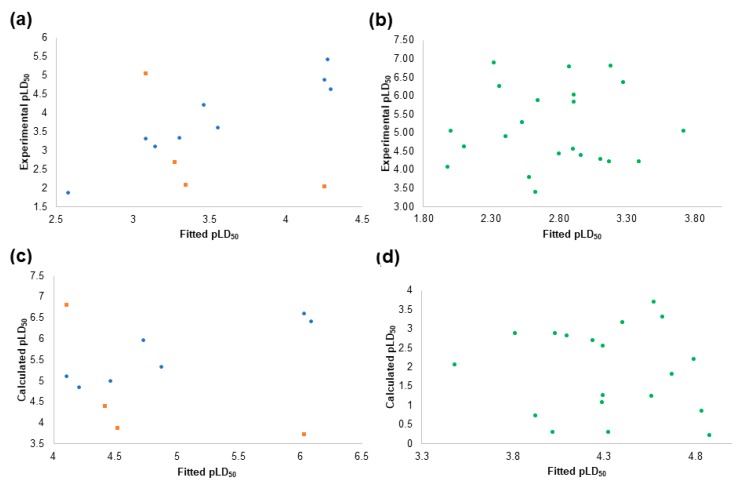
The plot of experimental vs. fitted *pLD*_50_ values calculated with QSAR model 2 for the training set pesticides (training set values are depicted by blue circles; internal test set values are indicated by orange squares) (**a**); test set pesticides (green circles) (**b**) against *Mus musculus*. The plot of calculated vs. fitted *pLD*_50_ values calculated with the QSAR model 4 for the training set pesticides (training set values are depicted by blue circles; internal test set values are indicated by orange squares (**c**); test set pesticides (green circles) (**d**) against *Homo sapiens***.**

Additional exclusion of diuron and monuron further increased the statistical robustness leading to a QSAR model 2 (Tables S5 and S6, Figure 2) with more than 90% of explained variance (90.5%) (Equation (3)):

(3) $$\;pLD_{50\;}=-0.0019E_{\mathrm{glob}\_\min}-3.05\;\left(r^{2}=0.905\right),$$

The obtained model 2 estimated the *pLD*_50_ values of carbofuran, linuron, diuron, and monuron, as in situ generated test set, with good accuracy (Figure 2a). On the other hand, model 2 was also used to predict the *Mus musculus pLD*_50_ values of the test set pesticides (Table 2 and Table S6, Figure 2b). However, despite the good statistical potential of model 2 (i.e., the explained variance value), the *E*_glob_min_ values cannot be considered as valuable indicators to anticipate the *Mus musculus pLD*_50_ values and for the acute toxicity prediction. 

Moreover, models 1 and 2 cannot be universally applied for the prediction of the *Homo sapiens pLD*_50_ as the *E*_glob_min_ values are common to both model organisms. Therefore, different models are required for *Homo sapiens* as model organism. In that sense, additional models were developed from calculated *Homo sapiens pLD*_50_ values (Equation (1)). As expected, the incorporation of all the training set, pesticides did not lead to any statistically significant model. As above, exclusion of carbofuran and linuron led to the model 3 (Equation (4)):

(4) $$\;\;\;\;\;\;pLD_{50\;}=-0.0021E_{\mathrm{glob}\_\min}+3.01\;\left(r^{2}=0.783\right),$$

and further exclusion of diuron and monuron led to model 4 (Equation (5), Tables S7 and S8, Figure 2c,d):

(5) $$\;\;pLD_{50\;}=-0.0019E_{\mathrm{glob}\_\min}+4.15\;\left(r^{2}=0.91\right),$$

Like for *Mus musculus* and *Homo sapiens*, the *pLD*_50_ values were not fully satisfactory correlated to the global minima energies, leading to the conclusion that *E*_glob_min_ values are not adequate indicators to anticipate the *pLD*_50_ against any model organism, the level was changed from LB to SB, directing that the free energies of binding could be considered as appropriate acute toxicity indicators. Free energies of binding can be calculated separately for each pesticide and model organism, by performing molecular docking studies, either for *Mus musculus* AChE or *Homo sapiens* AChE. 

### 2.5. The Structure-Based Studies

To get more insights into the mechanistic profile of understudied pesticides, their interactions with either *m*AChE and *h*AChE, as molecular targets, were also considered. Sequence similarity value between *m*AChE and *h*AChE was found to be 88.44% (Figure S1), rising to the 100% identity between [the active sites, indicating that similar pharmacodynamics profile should be expected with either *m*AChE or *h*AChE by the same molecule. Therefore, the application of SB tools could represent the computational approach to predict the biochemical mechanism and hence the level of acute toxicity. Two SB methodologies were chosen: molecular docking and molecular dynamics. Docking and dynamics studies were initially performed to anticipate the bioactive conformations of pesticides with known acute toxicity and unknown binding mode into the AChE, after which the obtained free energies of binding were correlated to the acute toxicities. Despite the fact there are no co-crystalized AChEs in complex with pesticides as either non-covalent or covalent inhibitors, it was arbitrarily decided to simulate reversible docking, for pure reversible AChE inhibitors, and reversible-like docking, for covalent AChE inhibitors, followed by the appropriate, one step molecular dynamics simulations. Reversible-like docking, was supposed to obtain the pre-covalent conformation that will lead to the AChE covalent modification by literature-claimed [73] covalent inhibitors. The ultimate task of SB studies was to reveal the free energies of binding and to correlate them to the pesticides acute toxicities.

#### 2.5.1. The Alignment Rules Assessment

In order to learn how to perform the SB alignment of targeted pesticides within the AChE active sites of either mouse or human molecular target, the 3-D coordinates of reversible AChE inhibitors were taken from their corresponding minimized complexes and used to gain the knowledge how to reproduce their co-crystallized conformations by means of the SB alignment assessment, as well as to determine the best SB molecular docking methodology. Within the SB alignment assessment, AutoDock [79], AutoDock Vina [80] (in the future text just Vina) and DOCK [81] were evaluated to select the best docking algorithm/scoring function and to perform the SB positioning of pesticides. The SB alignment assessment procedure was performed with the standard four step protocol [78]: Experimental Conformation Re-Docking (ECRD), Randomized Conformation Re-Docking (RCRD), Experimental Conformation Cross-Docking (ECCD), and Randomized Conformation Cross-Docking (RCCD).

Thus, within the ECRD stage, Vina and DOCK possessed the highest ability to reproduce the experimental conformations of *Mus musculus* and *Homo sapiens* AChE inhibitors. Even 75% of the available inhibitors were correctly reproduced by Vina, whereas DOCK was slightly less potent, reproducing the 50% of structures in either rigid or flexible manner, respectively (Tables S9 and S10). Similarly, Vina was of the highest potency in the initial reproduction of *Mus musculus* AChE inhibitors with the docking accuracy (DA) of 55.55% (Table S9). DOCK, however, completely failed in the reproduction the experimental poses of *Mus musculus* AChE inhibitors (Table S9). In the initial process, as well as in all other difficulty levels, AutoDock algorithm failed in the experimental conformations reposition. 

Similar potency for Vina and DOCK was observed within the RCRD stage in the process of human inhibitors reproduction (Table S10, Vina DA equal to 68.75%, DOCK DA equal to 56.25%, respectively). While processing mouse inhibitors (Table S9), Vina re-positioned the modelled inhibitors structures with the high efficiency (DAs equal to 57.78%), while DOCK’s potency dropped below 30%.

The level of difficulty was increased during the ECCD stage where either Vina or DOCK retained the high level of accuracy while cross-aligning of human inhibitors (Table S10). As for the other model organism, either Vina or DOCK were inaccurate while matching the co-crystallized inhibitors with diverse mouse enzymes (Table S9).

The decisive difference in favour of Vina, by means of the general docking accuracy, was noticed within the RCCD stage, as the most difficult one. The cross-alignment of randomized conformations is considered as the most important validation stage for any docking program as it represents the ability of a program to position a random ligand conformation in a non-native environment, i.e., into its molecular target whose active site amino acids suffered induced fit conformational change due to the presence of structurally different inhibitors. Within the RCCD stage, Vina was far superior in comparison with other programs, by means of The SB alignment accuracy, and, therefore, can be considered as a valuable tool for the prediction of bioactive conformations of acetylcholine esterase inhibitors with the unknown binding mode. 

#### 2.5.2. The Cross-Docking and Molecular Dynamics Studies of ACh

**Figure 3:**
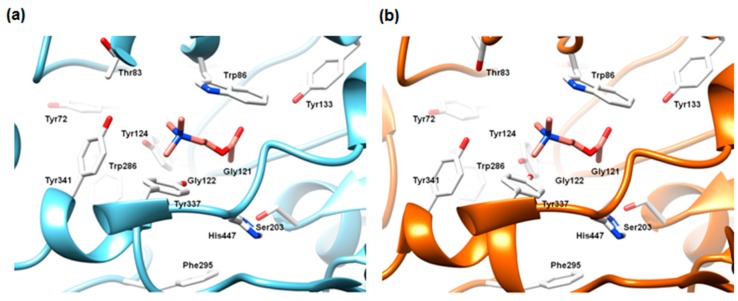
The SB alignment of acetylcholine into *m*AChE active site (**a**); *h*AChE active site (**b**). The enzyme ribbons are presented in blue and gold, respectively, active site amino acids are depicted in white. For the purpose of clarity, hydrogen atoms are omitted from the presentation. The graphic was generated using the UCSF Chimera software (Resource for Biocomputing, Visualization, and Informatics (RBVI), University of California, San Francisco, CA, USA).

Both the training set or the test set pesticides were submitted to the herein Vina-based proposed SB alignment protocols, that have been validated by reproducing the bioactive conformations of PDB-available inhibitors in complex with AChE (Supplementary Materials Tables S9 and S10, and Figure S2) and applied in situ in predicting the bioactive conformations of ACh (Figure 3).

As no experimental structures of ACh in complex with either wild-type *m*AChE or *h*AChE are available in the Protein Data Bank, its bioactive conformation within both enzymes was predicted by means of Vina, as the best performing SB alignment assessment tool (Figure 3a,b, see Supplementary Materials Alignment Rules Assessment section, Tables S9 and S10). The stability of either ACh-*m*AChE or ACh-*h*AChE complex was confirmed using one step molecular dynamics (MD) runs, upon the inspection of Cα RMSD values (1.2 ns simulation length, Figures S7a and S8a), RMSF values (Figures S7b and S8b), and trajectories energy terms (data not shown but available from the authors upon request). The short-termed MD protocol was sufficient to produce stable complexes. The two ACh poses (in *m*AChE or *h*AChE) were conformationally very close, displaying a RMSD value of 0.043 Å.

As expected, the positive quaternary ACh amine group was located in the anionic sub-site, where the orientation of particular group is supported by strong hydrophobic interactions with a *m*(*h*)Trp86 methyl group, as well as with *m*(*h*)Tyr124, *m*(*h*)Tyr337, and *m*(*h*)Tyr341 aromatic moieties (Figures S7c and S8c). The positively charged amine was electrostatically attracted by the electronic cloud of the *m*(*h*)Trp86 aromatic group. The acetyl group occupied the esterase sub-site: the carbonyl oxygen was involved in the electrostatic interactions with *m*(*h*)Tyr337 side chain hydroxyl group and *m*(*h*)His447 imidazole ring, while the carbonyl oxygen was oriented towards the side chain of *m*(*h*)Tyr133. Regarding the mouse enzyme, during the MD run, the carbonyl moiety was observed to be involved in the hydrogen bonding with *m*Tyr133 for 29% of simulation time (Figure S8c, *d*_HB_ = 1.943–2.337 Å) whilst for human enzyme the carbonyl group was mainly involved in the electrostatic interactions with *h*Ser203 and *h*His447 (Figure S8c). The analysis of docking and MD results agreed with the hydrolysis reaction pathway of ACh (Figure 1a) that was also externally confirmed by QM/MM studies elsewhere [82]. In fact, the ACh molecule had been approaching *m*(*h*)Ser203 during the simulation for the 57% of the time, where the range of distances between the acetyl group and the catalytic residue was 1.473–1.894 Å (Figures S7c and S8c).

#### 2.5.3. The Cross-Docking and Molecular Dynamics Studies of Pesticides 2-chloro-1,3,5-triazine Group

**Figure 4:**
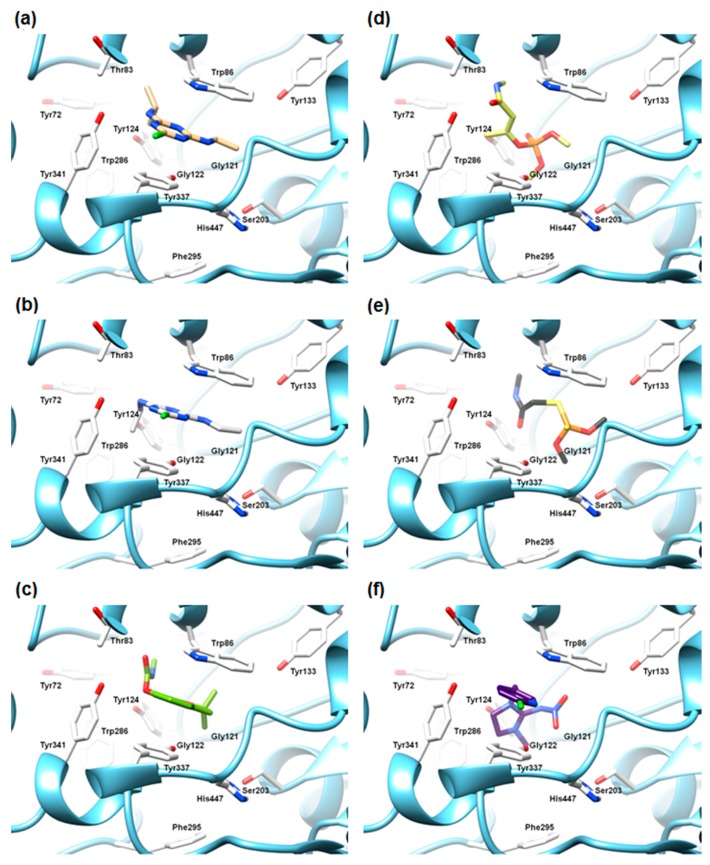
The SB alignment of atrazine (**a**); simazine (**b**); carbofuran (**c**); monocrotophos (**d**); dimethoate (**e**); imidacloprid (**f**) into the *m*AChE active site. The enzyme ribbons are presented in blue, the active site amino acids are depicted in white. For the purpose to clarify, the hydrogen atoms are omitted. The graphic was generated using the UCSF Chimera software (Resource for Biocomputing, Visualization, and Informatics (RBVI), University of California, San Francisco, CA, USA).

**Figure 5:**
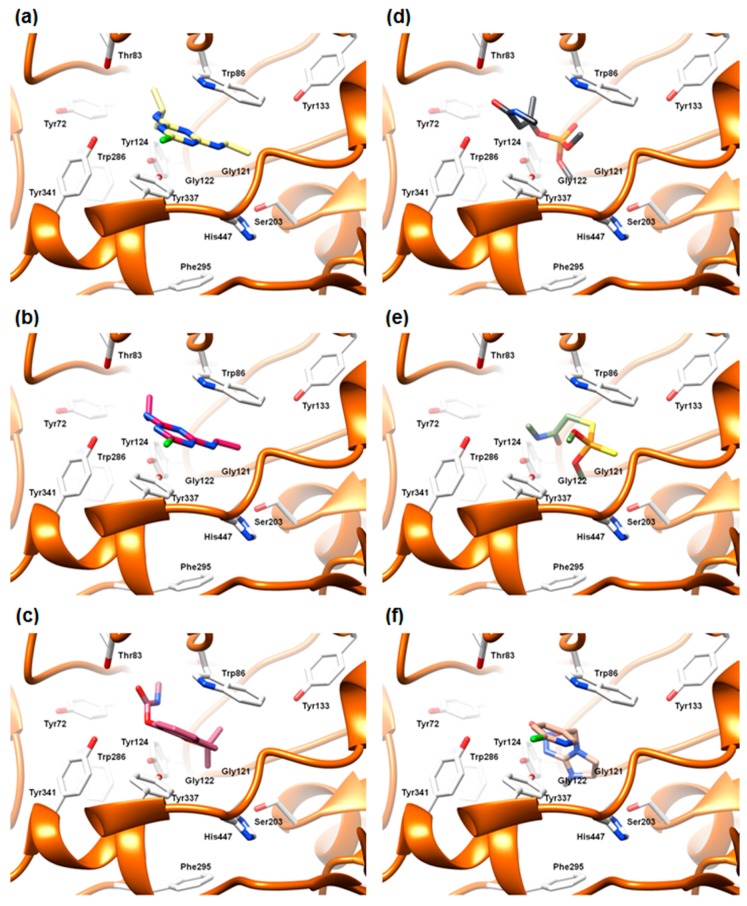
The SB alignment of atrazine (**a**); simazine (**b**); carbofuran (**c**); monocrotophos (**d**); dimethoate (**e**); imidacloprid (**f**) into the *h*AChE active site. The enzyme ribbons are presented in gold; the active site amino acids are depicted in white. For the purpose to clarify, the hydrogen atoms are omitted from the presentation. The graphic was generated using the UCSF Chimera software (Resource for Biocomputing, Visualization, and Informatics (RBVI), University of California, San Francisco, CA, USA).

Atrazine’s, simazine’s and propazine’s acute toxicity (Table 1) can be explained by means of their docked conformations, within either *m*AChE or *h*AChE (Figure 4 and Figure 5, respectively). The graphical interpretation of the results of the SB and MD studies are herein shown for atrazine (Figure 4a and Figure 5a, Figure S9 and Figure S10, respectively) and simazine (Figure 4b and Figure 5b, Figure S13 and Figure S14, respectively), whereas the corresponding illustrations considering the molecular modelling studies of propazine are reported as Supplementary Materials (Figure S3a, Figure S5a, Figure S11, Figure S12, respectively).

The best-docked poses of the triazine-based pesticides were found to occupy the ACh binding cavities of both the *m*AChE and *h*AChE. The main differences were not related to the predicted pesticides bioactive conformations, but were mainly ascribed to enzymes active site residue Thr83: the side chain methyl group of *m*AChE Thr83 pointed toward the anionic sub-site, whereas in the human enzyme the corresponding group is flipped out vertically from the active site. The latter caused the differences in the stabilization period during the MD runs: atrazine-*m*AChE, propazine-*m*AChE, and simazine-*m*AChE complexes were respectively of high stability after 0.100 ns, 0.120 and 0.610 ns (Figures S9a, S11a and S13a), whereas the complexes of human analogues equilibrated approximately 20 ps afterwards (Figures S10a, S12a and S14a). 

Nevertheless, within either mice or human bioactive conformations, the 2-chloro-1,3,5-triazines were parallel to *m*(*h*)Trp86 and *m*(*h*)Tyr337, trapped in the hydrophobic cage between two residues. The individual contribution of these interactions was respectively −37.34 and −19.9 kcal/mol, according to the Free-Energy Decomposition Analysis, FEDA (Table S11). Atrazine bioactive conformations were nearly identical in both enzymes. On the other hand, comparing simazine docked structures (Figure 4b and Figure 5b), the one found in *h*AChE (Figure 5b) was in-plane rotated for about 30°. The triazine nitrogen atom, *p*-positioned in relation to chlorine, was electrostatically stabilized by both the hydroxyl group of *m*(*h*)Tyr337 and the indole nitrogen of *m*(*h*)Trp86. The individual contribution of these interactions was respectively −34.26 and −15.56 kcal/mol, according to FEDA (Table S11). Within the *m*AChE, the *m*-*N*’ atoms, holding the isopropyl function of atrazine (Figure 4a), and one of the ethyl groups within simazine (Figure 4b), were oriented toward the *m*Thr83 methyl group and *m*Tyr341 phenolic moiety. All the described interactions were confirmed during the MD simulation (Figure S9c and Figure S13c, respectively). The atrazine *m*-*N*’ atom was involved in a strong hydrogen bonding (HB) (*d*_HB_ = 2.253–2.976 Å) with the *h*Thr83 hydroxyl group (Figure S10c) in 56% of MD run time. A similar interaction was not detected during the atrazine-*m*AChE complex MD run (Figure S9c), suggesting that the *h*Thr83 conformational flip and the existence of *h*Thr86-*m*-*N*′ HB may even increase the acute toxicity of atrazine to humans. Regarding the simazine, as a consequence of the in-plane rotation, its *m*-*N*’ atom created HB with *h*Tyr341 (*d*_HB_ = 2.341–3.127 Å, 76% of the time of simulation, Figure S14c). Furthermore, the functional groups that substitute the remaining *m*-*N*″ atoms were directed towards the *h*Tyr133, *h*Ser203, and *h*His447, and HB-attracted by *h*His447 (simazine established the strongest HB, *d*_HB_ = 2.379–3.242 Å, 94% of MD time, Figure S14c). Since the precise hydrogen bonds were not observed during the MD simulation within the *m*AChE, they may represent some indicators that the atrazine and simazine could exert even higher acute toxicity against humans than against mice. Moreover, the described docking/MD interactions were all confirmed by the FEDA application (Table S11).

Regarding propazine, the *m*-*N*′ atoms, holding the isopropyl units or propazin (Figure S5a) were oriented within the *m*AChE towards the side chain methyl group of *m*Thr83 and a phenolic moiety of *m*Tyr341. Similarly to atrazine and simazine, propazine may be even more toxic to humans than to mice, as it exerts the toxicity against humans by establishing the hydrogen bonds with *h*Tyr341 (*d*_HB_ = 2.286–3.114 Å) and *h*His447 (*d*_HB_ = 2.121–3.331 Å) in the 45% and the 95% of time, respectively (Figure S12c). Attracted to *m*Thr83, propazine was limited with the ability to make hydrogen bonds with *m*Tyr341 or *m*His447, as confirmed by MD studies (Figure S11c). 

#### 2.5.4. The Cross-Docking and Molecular Dynamics Studies of Pesticides Amide Group

Among the amide-based pesticides, carbofuran and monocrotophos were of the highest acute toxicity (Table 1). Therefore, the results of carbofuran (Figure 4c and Figure 5c, Figure S15 and Figure S16, respectively) and monocrotophos (Figure 4d and Figure 5d, Figures S17 and Figure S18, respectively) docking and MD studies are herein reported and discussed, whereas the corresponding graphical interpretations for lower toxic dimethoate (Figure 4e, Figure 5e, Figure S19, Figure S20, respectively), carbaryl and tebufenozide are reported as a Supplementary Materials (Figure S4, Figure S5, Figures S21–S24, respectively).

Carbofuran was the most toxic against *Mus musculus* (Table 1). According to the literature, this particular pesticide is, as a carbamate derivative, proclaimed to be the reversible AChE inhibitor [73]. The results of herein conducted SB studies are in full agreement with the proposed/confirmed reversible inhibition of AChE by carbofuran. Hence, within either *m*AChE or *h*AChE active site, the compound’s methyl methylcarbamate scaffold contributed as follows: the *N*-methyl group was positioned between the methyl group of *m*(*h*)Thr83 and *m*(*h*)Trp86 (Figure 4c and Figure 5c), a carbonyl group interfered with *m*(*h*)Tyr341 while the ether link was electrostatically stabilized by hydroxyl groups of *m*(*h*)Tyr124, *m*(*h*)Tyr337, and *m*(*h*)Tyr341, respectively. However, during the MD simulations, methylcarbamate displayed rotation towards the *m*(*h*)Tyr124 (Figures S15c and S16c) upon which the amide carbonyl group was involved in strong HB with *m*(*h*)Tyr124 for the 40% of time (*d*_HB_ = 2.478−2.783 Å, Figure S15c and Figure S16c, respectively), indicating a possible reason for carbofuran acute toxicity. The other HB was formed between the secondary amine and *m*(*h*)Trp86 (*d*_HB_ = 2.784−3.226 Å, Figure S15c and Figure S16c, respectively). Furthermore, the 2,2-dimethyl-2,3-dihydrobenzofuran part of carbofuran was located beneath the *m*(*h*)Trp86 and established hydrophobic interactions with *m*(*h*)Tyr337 due to the π-π stacking, as well as the electrostatic interactions with *m*(*h*)Tyr124 via 2,2-dimethyltetrahydrofuran oxygen atom. Moreover, all the described docking/MD interactions were confirmed upon conducting the FEDA procedure (Table S11).

The covalent mode of inhibition [73] and the high acute toxicity of organophosphorus-based pesticides monocrotophos and dimethoate against *Mus musculus* and *Homo sapiens* were primarily conditioned by the interactions of their amide groups with AChE. Hence, in the best docking pose of monocrotophos within *m*AChE, the amide carbonyl was oriented toward the hydroxyl group of *m*Tyr341 (Figure 4d). Such interaction was a result of hydrophobic stabilization between the pesticide’s allyl methyl group and the *m*Tyr341 phenyl ring, as well of the *N*-methyl group and *m*Thr83. Still, within the human enzyme (Figure 5d), corresponding amide carbonyl group approached the *h*Thr83 hydroxyl group, confirming that the orientation of hThr83 makes a difference in SB alignment in *h*AChE. The consequent important monocrotophos interactions were those generated by the dimethylated phosphoric acid. Thus, the phosphoric acid P=O group established electrostatic interferences with *m*AChE Tyr124 (Figure 4d); the precise oxygen atom served as an HB-acceptor to *m*Tyr124 during the MD simulation (*d*_HB_ = 2.227–2.946 Å, Figure S17c). For the purpose of *h*AChE inhibition, the corresponding monocrotophos carbonyl group was, due to the strong hydrophobic attraction between the phosphoric acid methoxy groups and *h*Trp86, only partially directed towards the *h*Tyr124 (Figure 5d). However, monocrotophos adapted its conformation/binding upon 0.718 ns of MD simulations with *h*AChE (Figure S18a), and, contrarily to *m*AChE, *h*Tyr124 was involved in 96% of the time in HB with the amide carbonyl (*d*_HB_ = 1.984–2.117 Å, Figure S18c). Moreover, one of the phosphoric acid oxygen atoms made HB with *h*Tyr337 (35% of simulation time, *d*_HB_ = 2.449–2.688 Å, Figure S18c). Still, inhibiting *m*AChE or *h*AChE, the monocrotophos phosphoric acid moiety remained situated in the proximity of *m*(*h*)Ser203 and *m*(*h*)His447 (the average distances: 1.25–1.783 Å for *m*AChE, 1.375–1.651 Å for *h*AChE, Figure S17c and Figure S18c, respectively), suggesting that the covalent modification of *m*Ser203 or *h*Ser203 will occur in vivo [74]. Moreover, all the described docking/MD interactions were confirmed upon conducting the FEDA (Table S11).

The monocrotophos structural analogue, dimethoate, is, as *Mus musculus* and *Homo sapiens* AChE inhibitor, limited with the capacity to interfere with *m*(*h*)Tyr341 by means of steric interactions, inasmuch as it does not contain the allyl methyl group (Figure 4e and Figure 5e, respectively). Hence, although dimethoate is in both active sites superimposed to monocrotophos, by means of backbone orientation (Figure 4d and Figure 5d, respectively), its amide carbonyl is oriented towards *m*(*h*)Tyr124 and *m*(*h*)Tyr337. The bioisosteric replacement of the phosphoric acid carbonyl group with the thione one influenced that the sulfur atom is within the mouse enzyme directed to the hydroxyl group of *m*(*h*)Tyr337, while within the human one the thione moiety is in reach of the *m*(*h*)Ser203 and *m*(*h*)His447.

**Figure 6:**
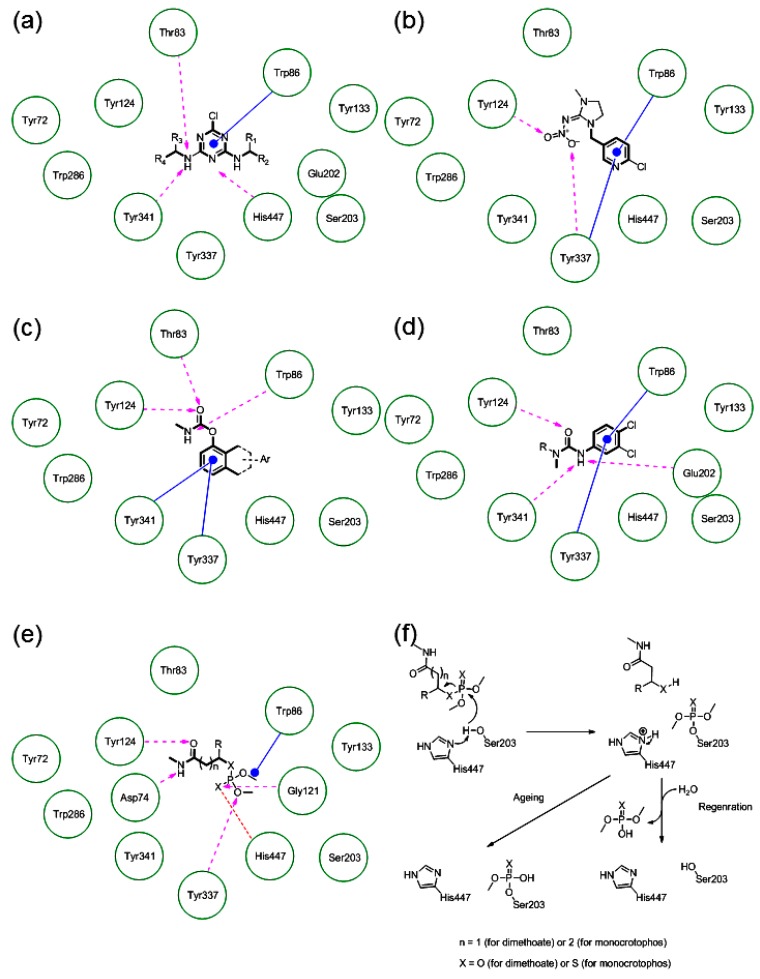
Schematic view of pesticides binding interactions within the *h*AChE active site: atrazine, simazine or propazine (**a**); carbofuran or carbaryl (**b**); monocrotophos or dimethoate (**c**) imidacloprid or acetamiprid (**d**); diuron, monuron, or linuron (**e**). The hydrogen bonds are presented with pink dashed half arrows, the hydrophobic interactions with a blue dot-line marker, the ionic interactions with the red dashed lines. The proposed mechanism of monocrotophos or dimethoate acute toxicity against *h*AChE (**f**).

However, surprisingly, there was a little consistency between the best-docked structure of dimethoate within the mouse active site (Figure 4e) and the molecular dynamics solutions (Figure S19). Thus, pesticide’s amide carbonyl established no interactions, while the amide nitrogen was periodically involved in hydrogen bonding with *m*Tyr341 time (*d*_HB_ = 2.971–3.557 Å, Figure S19c). Thione functional group was HB-acceptor to *m*His447 (25% of the time, *d*_HB_ = 2.894–3.431 Å), while one of the methoxy functions was in the HB-vicinity to *m*Gly121 (27% of the time, *d*_HB_ = 2.723–3.338 Å). Those interactions allegedly provide the opportunity for *m*Ser203 to perform the electrophilic attack on pesticide (Figure 6f). On the other side, there was a high level of agreement between the dimethoate rigid docking (Figure 5e) and molecular dynamics within the human enzyme, by means of the amide carbonyl group interactions, given that this pharmacophore was for the 95% of time included in hydrogen bonding with *h*Tyr124 (*d*_HB_ = 1.643–2.128 Å, Figure S20c). Moreover, one of the phosphoric acid oxygens was, as for monocrotophos, used to make a hydrogen bond with *h*Tyr337 (35% of simulation time, *d*_HB_ = 2.449–2.688 Å, Figure S20c). In general, considering the overall orientation of the molecule, dimethoate would most likely behave as a covalent inhibitor of human AChE (Figure 6f), as well. 

Carbaryl reached the comparable bioactive conformation to carbofuran (Figures S3b and S5b), within both species AChE active sites, with the difference that the 2,2-dimethyltetrahydrofuran scaffold is in the structure of particular pesticide replaced with another benzene ring. Such replacement has a direct consequence in a diminished toxicity of carbaryl in comparison with carbofuran, against either mice or humans, since carbaryl cannot establish additional electrostatic interactions with *m*(*h*)Tyr124.

Regarding carbaryl, it is proclaimed (Table 1) that this pesticide is more than fifty times less toxic than carbofuran when administered orally to *Mus musculus*, and MD revealed the reason. Hence, within the *m*AChE, carbaryl’s amide carbonyl group forms a hydrogen bond, not with the *m*Tyr124, but with the HB-network made of ordered water molecules and *m*Asn87 (Figure S21c). The precise function also establishes the pure HB with *m*Ser125 (75% of simulation time, *d*_HB_ = 2.335–2.412 Å, Figure S21c). However, according to the MD study performed on the carbaryl-*h*AChE complex, there is a 36% of probability that particular carbonyl group is oriented towards HB with *h*Tyr124 (*d*_HB_ = 2.374–2.894 Å, Figure S22c), thus anticipating the high toxicity of this pesticide to humans.

The lowest active amide-based pesticide is tebufenozide (Table 1). Almost symmetrical as a compound, this pesticide can be divided in two scaffolds by which it establishes the interactions within the active site of either *m*AChE or *h*AChE: the 4-ethylbenzamide and the *N*-*tert*-butyl-3,5-dimethylbenzamide (Figure S3c and Figure S5c, respectively). The RMDS value between the mouse and human docked conformations amounts 0.472 Å, implying the high similarity between the obtained conformations. Thus, the ethylbenzene scaffold of 4-ethylbenzamide was oriented parallel to benzene core of *m*(*h*)Tyr337, establishing hydrophobic interactions. The carbonyl group that completes the 4-ethylbenzamide was electrostatically attracted by three active site residues: *m*(*h*)Tyr124, *m*(*h*)Tyr337 and *m*(*h*)Tyr341. The hydrophobic part of *m*(*h*)Tyr124 additionally stabilized the *tert*-butyl group, incorporated in the second part of pesticide. The carbonyl group belonging to the *tert*-butyl-3,5-dimethylbenzamide moiety was geared towards the amide group of *m*(*h*)Gln71 and the hydroxyl group of *m*(*h*)Tyr124, for the purpose of forming the electrostatic interactions. The remaining part of the distinct subsection, in the form of 3,5-dimethylbenzene, is stabilized with the hydrophobic parts of *m*(*h*)Trp86 and *m*(*h*)Phe295. Similarly to other amide-based pesticides, this pesticide exerts the toxicity after creating the strong hydrogen bond in-between the *tert*-butyl-3,5-dimethylbenzamide moiety and either *m*Tyr124 or *h*Tyr124 (95% of simulation time, *d*_HB_ = 1.273–1.588 Å, Figure S23c; 94% of simulation time, *d*_HB_ = 1.268–1.575 Å, Figure S24c). Beside those beneficial interferences, the pesticide owns its stability and consequent toxicity to numerous hydrophobic interactions (Figure S23c and Figure S24c, respectively).

#### 2.5.5. The Cross-Docking and Molecular Dynamics Studies of Pesticides (6-Chloropyridin-3-Yl)Methanamine and 1-(4-Chlorophenyl)-3-methylurea Groups

The pesticides comprising the two remaining groups exerted the lowest acute toxicities to *Mus musculus* (Table 1). The results of SB and MD studies are herein shown for imidacloprid (Figure 4f and Figure 5f, Figure S25 and Figure S26, respectively), while the corresponding exemplifications considering the molecular modelling studies for lower toxic acetamiprid, diuron, monuron, and linuron are available as Supplementary Material (Figures S3–S6 and Figures S27–S34, respectively).

Regarding the best docking poses of imidacloprid within either *m*AChE or *h*AChE, these indicated that the 2-chloro-5-methylpyridine moiety was sterically stabilized by *m*(*h*)Trp86 and *m*(*h*)Tyr337 (Figure 4f and Figure 5f, respectively). The difference in the alignment occurred with the *N*-(imidazolidine-2-ylidene)nitramide scaffold, whose nitro group was oriented towards *m*Ser203, while the same function in human enzyme was in the proximity of *h*Tyr124. Even though the imidacloprid-*m*AChE complex was more stable in comparison to the human one (Figure S25a), interactions generated within the complex were inconsistent to those that imidacloprid established with *h*AChE. Thus, while interacting with *m*AChE (Figure S25c), the nitro group was involved in the hydrogen bonding with *m*Gly122 (64% of MD time, *d*_HB_ = 2.483–2.788 Å) and *m*Ala204 (59% of MD time, *d*_HB_ = 2.771–3.132 Å). The corresponding functional group in *h*AChE (Figure S26c) was a HB acceptor for *h*Tyr124 (82% of MD time, *d*_HB_ = 1.664–1.738 Å) and *h*Tyr337 (68% of MD time, *d*_HB_ = 1.825–1.931 Å), and further confirmed those residues as essential for pesticides acute toxicity in humans. Moreover, all the described docking/MD interactions were confirmed by FEDA (Table S11).

The important interactions anticipated by the molecular docking and molecular dynamics (Figure 6), provided the basis for the understanding of the general mechanism of acute toxicity on the reversible and reversible-like inhibitors level. Even though the covalent AChE inhibitors are highly efficient as pesticides, the future pesticides production must be based on the reversible-like interactions, avoiding the pesticide-AChE complex regeneration or ageing [73]. The obtained reversible-like docking and MD poses for covalent AChE inhibitors clearly confirmed the potency of selected computational tools (Vina/Desmond programmes pair) to predict the covalent binding mode of any compound in future considered as covalent AChE inhibitor (i.e., the covalent pesticide). Therefore, there was no point to conduct the covalent docking that would be reduced just to a conformational search of the bonded molecules. Hereby applied docking/MD application was also aimed to assess the docking/MD software real utility to investigate the covalent AChE inhibitors. It must be emphasized that any inhibitor, even covalent, must undergo equilibrium between the free and the bonded stage. Therefore, the reversible-like (pre-covalent) pose is of extreme utility to predict the ligand conformation that will proceed to make a covalent bond. This information could be used in future works to design new reversible inhibitors (pesticide).

Considering the docking poses of acetamiprid (Figure S3d and Figure S4d, respectively), pesticide’s 2-chloro-5-methylpyridine moieties were aligned to each other in both enzymes and located in-between the *m*(*h*)Trp86 and *m*(*h*)Tyr337. The toxicity of particular pesticide may be additionally seen through the interactions of (*E*)-*N*-ethyl-*N*′-ethynyl-*N*-methylacetimidamide function, linked directly to the 2-chloropyridine ring and located in the spatial pocket made of *m*(*h*)Trp86, *m*(*h*)Gly121, *m*(*h*)Tyr133, *m*(*h*)Ser203, and *m*(*h*)His 447. However, according to the molecular dynamics results (Figure S27c and Figure S28c, respectively), this part of the pesticide has not achieved significant interactions with the specified site of amino acids, and the low toxicity of acetamiprid may be attributed only to the hydrophobic interactions established between the 2-chloro-5-methylpyridine and *m*(*h*)Trp86 and *m*(*h*)Tyr337. 

Concerning the remaining compounds, diuron, linuron, and monuron, the basic scaffold they share is the 1-(4-chlorophenyl)-3-methylurea. In the structure of diuron, the terminal amine is dimethyl substituted while the aromatic moiety bears additional *m*-Cl substituent. Matching the conformations of diuron generated in *Mus musculus* and *Homo sapiens* AChE (Figure S4a and Figure S6a, respectively), conclusion can be made that the structures were almost perpendicular to each other, by means of aromatic rings positioning: the aromatic scaffold in mouse enzyme active site was perpendicular to *m*Trp86 and *m*Tyr337, while in the human environment this scaffold adopted parallel orientation related to *h*Trp86 and *h*Tyr337 (analogously to previous compounds). Considering the *m*AChE, the only reasonable explanation for such alignment of diuron is the interaction of *m*Thr83 methyl group with the pesticide aromatic scaffold; the *m*Thr83 side chain was positioned inside the enzyme active site and not out of it, like in humans (Figure S4a). Because of the different alignment, the carbonyl group of diuron′s 1,1-dimethylurea scaffold was oriented towards the *m*Tyr124, *m*Ser203, *m*Tyr337, and *m*His447 (Figure S4a). In the human environment (Figure S6a), the exact carbonyl group was directed towards the *h*Trp86 and *h*Tyr133. The MD simulation of mouse complex confirmed the weak hydrogen bonding between the urea carbonyl group and *m*Tyr124 (31% of simulation time, *d*_HB_ = 2.872–3.132 Å, Figure S29c), while the corresponding interaction could not be established in human surroundings (Figure S30c). The low intensity of precise hydrogen bond may be the reason of low toxicity of diuron, latter on concluded for linuron, and monuron, too.

Speaking of the remaining derivatives, the aromatic scaffolds of monuron (Figure S4b and Figure S6b, respectively), and linuron (Figure S4c and Figure S6c, respectively) were in a sandwich between the *m*(*h*)Trp86 and *m*(*h*)Tyr337, in either mouse or human enzyme active site. The monuron carbonyl group was oriented towards the *m*(*h*)Tyr133 and *m*(*h*)Ser203, whereas the particular group in linuron is headed to *m*(*h*)Tyr 124, *m*(*h*)Tyr133 and *m*(*h*)Ser203. The nature of monuron and linuron carbonyl groups interactions with specified amino acid had been revealed after the MD studies. Thus, for either mouse or human AChE-based bioactive conformations, the carbonyl group of monuron interfered with *m*(*h*)Tyr124 in a manner of moderate strength hydrogen bonding (within the mouse enzyme active site during the 62% of simulation time, *d*_HB_ = 2.548–3.012 Å, Figure S31c; within the human enzyme active site during the 56% of simulation time via the ordered water molecule, *d*_HB_ = 2.936–3.326 Å, Figure S32c). Similar interactions were observed for linuron as well (within the mouse enzyme active site during the 56% of simulation time via the ordered water molecule, *d*_HB_ = 2.985–3.455 Å, Figure S33c; within the human enzyme active site during the 90% of simulation time, *d*_HB_ = 2.847–3.278 Å, Figure S34c). 

#### 2.5.6. The Cross-Docking of Externally Evaluated Pesticides

The structure-based studies by means of reversible and reversible-like bioactive conformations generation, recommended Vina as a tool for pesticides cheminformatics treating. Therefore, Vina was used to predict the global minimum conformation of test set pesticides (Table 2) as well. 

The results of molecular docking are presented as a Supplementary Materials (Figures S35–S42). The generated conformations were used further on for the external validation of the training set results. 

### 2.6. The Pesticides Acute Toxicity Anticipation through the Bioactive Conformations

Upon conducting the reversible and reversible-like molecular docking and molecular dynamics studies, for either training or test set pesticides, the acute toxicity was considered with regard to the established bioactive conformations. Therefore, similarly as above reported for global minima energies, monoparametric QSAR model was derived to initially describe the acute toxicity against *Mus musculus* by enumerating the acute toxicities through the free energies of formation of reversible and reversible-like binding for all the training set pesticides (model 5, Equation (6), Table S12):

(6) $$\;\;\;\;pLD_{50\;}=-0.7885\Delta G_{\mathrm{binding}}-2.44\;\left(r^{2}=0.95\right),$$

**Figure 7:**
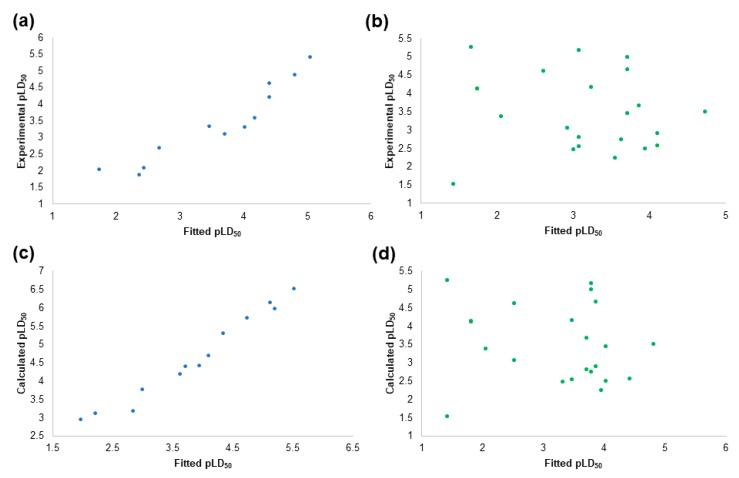
The plot of experimental vs. fitted *pLD*_50_ values calculated with QSAR model 5 for training set pesticides (**a**); test set pesticides (**b**) against *Mus musculus*. The plot of calculated vs. fitted *pLD*_50_ values calculated with QSAR model 5 for training set pesticides (**c**); test set pesticides (**d**) against *Homo sapiens*.

A satisfactory model was obtained by means of the whole training set and was used to recalculate the *pLD*_50_ values for acute toxicity against *Mus musculus*, for both the training and test sets (Figure 7a,b, Tables S12 and S13). Afterwards, the model was used to predict the *pLD*_50_ values for acute toxicity against humans on the basis of *∆G*_binding_ values for *Homo sapiens* AChE (Figure 7c,d, Tables S14 and S15). 

Thus, the QSAR model 5 described the acute toxicities against *Mus musculus*, by means of the free energies of binding of reversible and reversible-like bioactive conformations for either non-covalent or covalent pesticides, respectively. The model is also proven to be a tool for the anticipation of acute toxicities against humans. In the end, the conduced structure-based studies unequivocally supported *pLD*_50_/∆*G*_binding_ interrelation obtained by QSAR model 5. In that sense, the SB approach, conducted through Vina as the cheminformatics engine, was proven to be the correct one for the anticipation of acute toxicities of targeted pesticides. Consequently, the Vina-based ∆*G*_binding_ values can be considered in future design as valuable indicators to anticipate the pesticides LD_50_ values against any model organism. 

### 2.7. The Quantum Mechanical Studies of Acetylcholinesterase Inhibition by 2-Chloro-1,3,5-triazine-based Pesticides

The performed molecular docking and molecular dynamics studies agreed with the previously reported mode of action of amide-based, 6-chloropyridine-3-yl)methanamine-based, and 1-(4-chlorophenyl)-3-methylurea-based pesticides [73]. The quantum mechanical (QM) calculations were only performed on *h*AChE, with the aim to reveal the pharmacology of 2-chloro-1,3,5-triazine-based pesticides and the origin for the acute toxicity, given that their pharmacology is the least understood. 

As molecular docking and molecular dynamics studies indicated the formation of hydrogen bonds network between the atrazine, propazine, and simazine and *h*AChE, the HB-bearing pesticides-*h*AChE MD poses were extracted from pesticides-*h*AChE complexes and subjected to quantum mechanical (QM) DFT mechanistic studies. Thus, the DFT-based calculations were performed on two different levels. The first level included the extraction of MD geometries where favourable hydrogen bonds between the 2-chloro-1,3,5-triazine-based pesticides and *h*Thr83, *h*Tyr341, *h*Tyr124, and *h*His447, respectively, were found. Subsequently, the extracted pesticide-*h*Thr83, pesticide-*h*Tyr124, pesticide-*h*Tyr341, and pesticide-*h*His447 HB forming geometries were QM optimized in order to locate the transition states (TSs) and/or the intermediary states (ISs) by which the protons transfers occur in the particular segment of biochemical reaction. The second level of calculation attempted to find the identical TSs and/or ISs upon the manual adjustment of starting pesticide-*h*Thr83, pesticide-*h*Tyr124, pesticide-*h*Tyr341, and pesticide-*h*His447 geometries, by means of the TS-generating hydrogen atom position between the pesticide and the regarded AChE residue, in a kind of validation process. Each of the reaction pathways assumed the contemplation by means of Intrinsic Reaction Coordinate (IRC) analysis. Remarkably, the independent calculations gave similar/identical results, confirming that each of the obtained TSs and/or ISs is not formed by chance.

**Figure 8:**
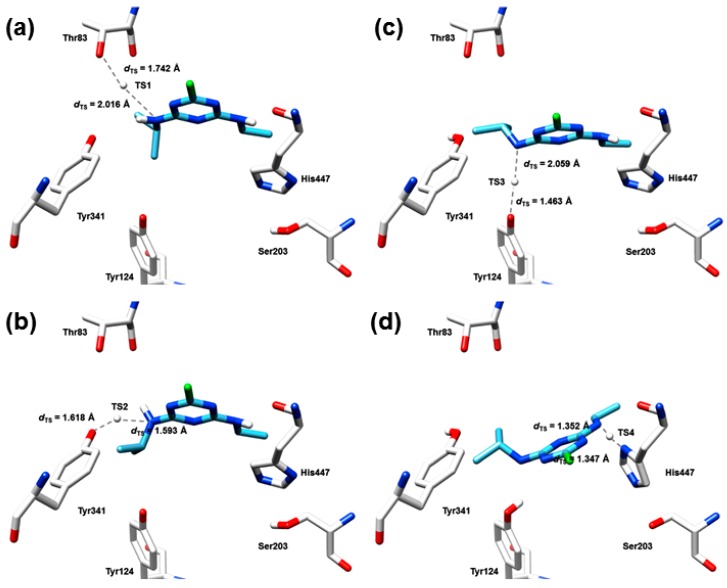
The quantum chemical mechanism of *Homo sapiens* AChE inhibition by atrazine. The extracted geometry of TS1 (**a**); TS2 (**b**); TS3 (**c**); TS4 (**d**).

**Scheme 1:**
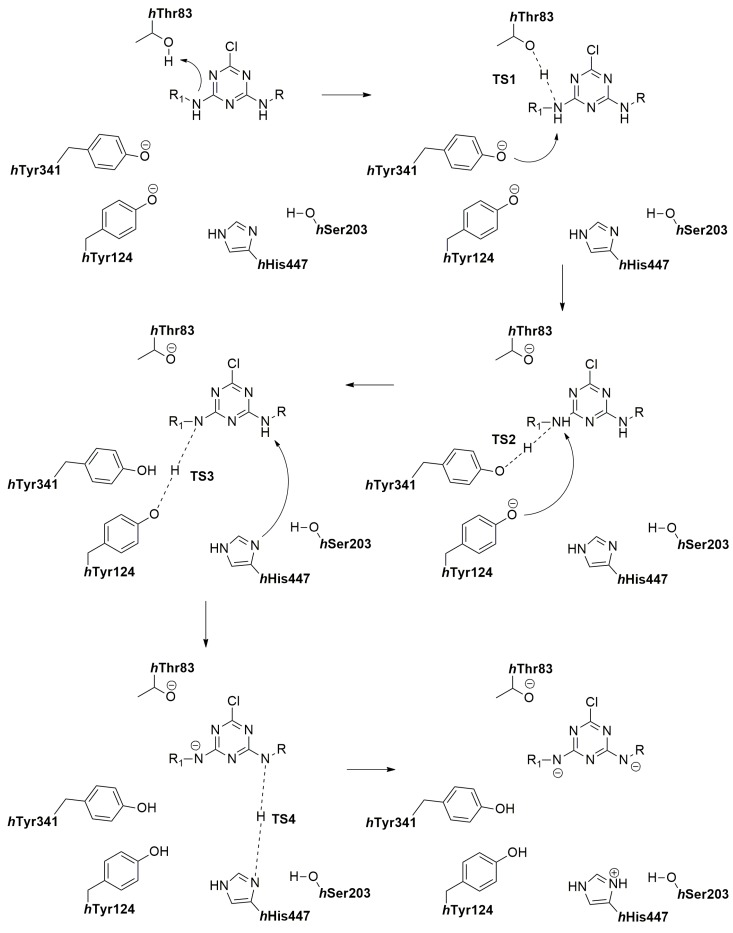
The mechanism of *Homo sapiens* AChE inhibition by atrazine, propazine, and simazine, verified by QM studies.

**Figure 9:**
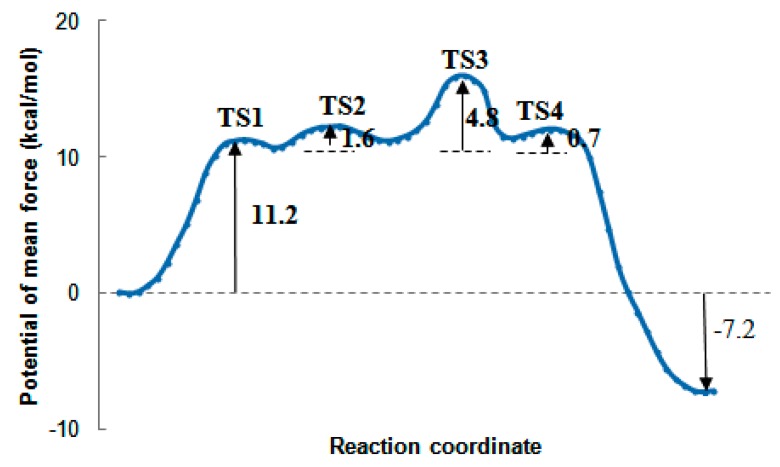
Free energy profile for *Homo sapiens* AChE inhibition by atrazine determined by B3LYP (6-31G*) QM simulations.

Thus, the optimization of atrazine-*h*Thr83, atrazine-*h*Tyr124, atrazine-*h*Tyr341, and atrazine-*h*His447 HB geometries confirmed that the proton transfer, within each of the geometries, occurs via the adequate transition states. Hence, the inhibition of *h*AChE starts with the nucleophilic attack of atrazine’s *m*-*N′* atom hydrogen towards *h*Thr83 (Figure 8a). The distance between the *h*Thr83 hydroxyl group oxygen atom as a HB acceptor and *m*-*N′* atom as a HB donor amounts 3.758 Å, characterizing particular HB as weak, almost electrostatic by character. This is, certainly, the expected increase in atrazine-*h*Thr83 HB distance, arisen following the QM optimization, in comparison to the one recorded during the MD simulations (*d*_HB_ = 2.253−2.976 Å). Nevertheless, even the weak HB is enough to increase the acute toxicity of atrazine to humans, in comparison to the propazine and simazine, inasmuch as the latter pesticides are, according to the QM studies, unable to donate proton for the formation of described TS1. Instead, the propazine-*h*Thr83 and simazine-*h*Thr83 interactions (Figure S43a and Figure S44a, respectively) were characterized by the IS1; the optimized QM distance between the *h*Thr83 hydroxyl group oxygen atom and propazine and simazine *m*-*N′* atom was 4.259 Å and 4.281 Å, respectively, suggesting the electrostatic nature of the established bond instead of the HB one. Back to the atrazine, upon the formation of atrazine-*h*Thr83 TS1 (Figure 8a and Scheme 1), the deprotonation of *h*Thr83 side chain hydroxyl group occurs, yielding the negatively charged oxygen atom, whereas at the same time *m*-*N′* atom becomes positively charged. Identical TS1 conformation and reaction pathway was obtained upon the manual setup and the optimization of the transition state geometry. The calculated activation energy barrier for the generated TS1 is 11.2 kcal/mol and this initial reaction is the rate-limiting step for the *h*AChE inhibition by atrazine (Figure 8a and Figure 9). The Free energy profile of all of the described reaction pathways was presented similarly to the study describing the catalytic reaction mechanism of AChE on the ACh level [82]. The manual setup of TS1 coordinates and its optimization resulted in the fact that the 47% of IRC described the proton transfer from TS1 to *h*Thr83, whereas the 53% or IRC quantified the proton transfer from TS1 to atrazine *m*-*N′* atom. This result further supported the proposed nucleophilic attack; the proton transfer is concerted, and the characterized transition state is meaningful. The precise atrazine-*h*Thr83 TS1/hydrogen bond is formed and is stable at all transition and intermediate states during the inhibition process.

The atrazine-based *h*AChE inhibition further flows towards the deprotonation of *m*-*N′* atom (Figure 8b and Scheme 1) where *h*Tyr341 phenoxy anion serves as a HB-acceptor. The deprotonation concerted via the TS2, in which the distance between the electronegative atoms amounts 3.211 Å. The similar transition states (this time the TS1s) were also observed for propazine and simazine (Figure S43b and Figure S44b, respectively), and for peculiar pesticides this was the rate-limited reaction (Figure S45a,b). The calculated activation energy barriers for the generation of propazine-*h*Thr83 and simazine-*h*Thr83 TS1 complexes were 8.5 and 8.9 kcal/mol, respectively (Figure S45a and Figure S45b, respectively). To adopt the atrazine-*h*Tyr341 TS2 geometry (Figure 8b and Scheme 1), atrazine is slightly in-plane rotated towards *h*Tyr341, whereas propazine and simazine suffered more severe longitudinal movement (Figure S43b and Figure S44b, respectively). Considering that the energy difference between the atrazine-*h*Tyr341 reactant state and TS2 is only 1.6 kcal/mol (Figure 9), an extra stabilization comes with the formation of atrazine-*h*Tyr341 TS2. The length of atrazine-*h*Tyr124 HB characterizes it as a moderate one. The second level of QM optimization also confirmed the meaningfulness of TS2. As previous, the meaningfulness of TS2 is confirmed since the 56% of IRC described the proton gliding from TS3 to *h*Tyr341, whereas the 44% or IRC illustrated the proton transfer form TS2 for atrazine *m*-*N′* atom. Similar trends were noticed for propazine-*h*Tyr341 and simazine-*h*Tyr341 TS1 (Figure S43b and Figure S44b, respectively). Nevertheless, during the rotation of atrazine (the movement of propazine or simazine), the TS1 (IS1) is sustained (Figure S43b and Figure S44b, respectively).

In the third step of the reaction pathway, the electrophilic nature of the re-established secondary *m*-*N′* amine is expressed by the release of proton towards the *h*Tyr124 hydroxyl group anion. The proton transfer takes place via the TS3, in which the distance between the electronegative atoms amounts 3.522 Å (Figure 8c and Scheme 1). For the purpose of TS3 generation, atrazine is forced to move longitudinally in order to form the tetrahedral structure with the HB-involved residues. Therefore, this step is slightly energetically more expensive, with the free energy difference of 4.8 kcal/mol (Figure 9). The characteristic of the tetrahedral TS3 structure is that it holds the negatively charged secondary *m*-*N′* nitrogen atom. The distinct state is supported by the aromatic nature of the atrazine’s 1,3,5-triazine ring, which can accept the *m*-*N′* amine negative charge by the intramolecular resonance stabilization. As mentioned above, the meaningfulness of the TS3 is confirmed since 57% of IRC described the proton gliding from TS3 to *h*Tyr124, whereas the 43% or the IRC illustrated the proton transfer from TS3 to atrazine’s *m*-*N′* atom. On the other hand, the propazine-*h*Tyr124 and the simazine-*h*Tyr124 interactions are characterized by the IS2 (Figure S43c and Figure S44c, respectively), in which the QM optimized distances between the *h*Tyr124 and the propazine and simazine were 4.882 and 3.862 Å, respectively. 

Moreover, the formation of the atrazine-*h*AChE tetrahedral TS3 structure appears to be the pre-condition for *h*His447 to be jointly arranged with the atrazine (Figure 8d and Scheme 1). In this last step of the reaction pathway, the atrazine’s *m*-*N″* amine suffers the nucleophilic attack from *h*His447 imidazole ring, in which the proton transfer is carried out from the pesticide towards the amino acid via the TS4 (the HB distance was 2.699 Å). Consequently, there is a localization of the negative charge around the *m*-*N″* amine as well, which can be once again efficiently delocalized by the atrazine′s 1,3,5-triazine ring. The similar pathway is observed for the propazine-*h*His447 and simazine-*h*Tyr447 interaction, with the transition state labelled as TS2 (the HB distances were 2.583, and 2.540 Å, respectively). In comparison with the TS4 (TS2) optimized MD snapshot, the orientation of *h*His447 needs to be adjusted very slightly, following which there is a proton transfer from the atrazine/propazine/simazine *m*-*N″* amine to the residue accompanied with the spontaneous break of the scissile *m*-*N″*-H bond (Figure 8d, Figure S43d and Figure S44d, respectively). As a result, a short and low-barrier hydrogen bond (LBHB) is formed between the *h*His447 and the atrazine’s/propazine’s/simazine’s *m*-*N″* amine (Figure 8d, Figure S43d and Figure S44d, respectively), and the willing proton transfer is thus observed. Energetically, this is the most favourable reaction since the free energy differences between the reactant and transition states were 0.70, 0.68, and 0.62 kcal/mol, respectively. Alongside with the TS1, the TS4 (TS2) is by far the most important transition state as it interrupts the *h*His447 to serve as a base for *h*AChE catalytic triad. By all means, the meaningfulness of TS4 is confirmed since the 51% of IRC described the proton gliding from TS3 for *h*His447, whereas the 49% or IRC illustrated the proton transfer form TS4 for atrazine *m*-*N′* atom (Figure 8d and Scheme 1). Similar trends were also noticed for propazine-*h*Tyr341 and simazine-*h*Tyr341 TS2 (Figure S43d and Figure S44d, respectively). Nevertheless, during the formation of the atrazine-*h*His447 TS4 (propazine-*h*His447 and simazine-*h*Tyr447 TS2), the TS1 (IS1), TS2 (TS1), and TS3 (IS2) were sustained.

The quantum mechanical studies do not provide the basis for the *h*Ser203 covalent modification by atrazine’s, propazine’s or simazine’s *m*-*N″* atom substituent, despite the fact that in metabolic pathways pesticides may release the particular functional group. The literature survey supports particular findings since the metabolites from atrazine, simazine and propazines are not able to establish the interactions with acetylcholinesterase [83]. This statement was further confirmed in the concentration dependent kinetic studies.

### 2.8. The Concentration-Dependent Kinetics of Human Acetylcholinesterase Inhibition by 2-Chloro-1,3,5-triazine-based Pesticides

**Figure 10:**
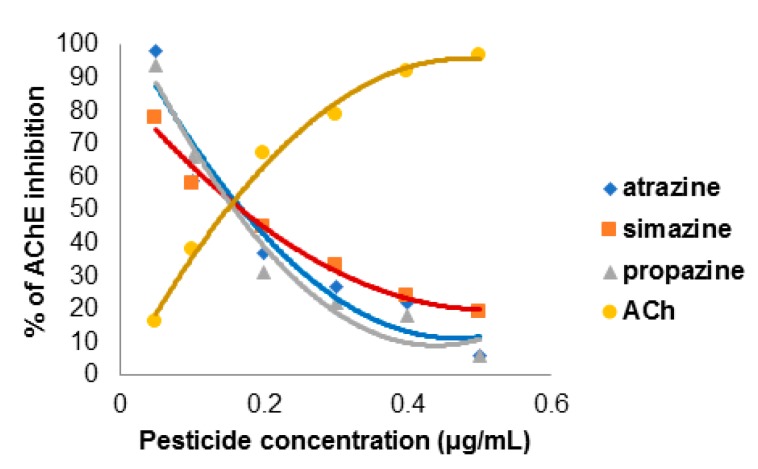
Human AChE inhibition by 2-chloro-1,3,5-triazine-based pesticides.

There are numerous evidences that organophosphates are irreversible AChE inhibitors whereas the carbamates acts as reversible AChE inhibitors [73]. However, studies show that atrazine decreases the AChE catalytic activity in *Chironomus tentans* [84] and *Carassius auratus*, applied synergistically with organophosphate insecticides, [80]. Nevertheless, the data describing the atrazine’s and the related compounds individually influence the *h*AChE catalytic activity is limited. Therefore, the 2-chloro-1,3,5-triazine-based pesticides are herein evaluated as *h*AChE inhibitors, in the concentrations comparable to the calculated LD_50_ values for *Homo sapiens* (Figure 10).

The results show that all of the examined 2-chloro-1,3,5-triazine-based pesticides inhibit *Homo sapiens* AChE activity in a concentration-dependent manner (Figure 10). The inhibition curves analysis implies that the aging reaction, in which the *h*AChE releases itself from covalently bound pesticide, occurs in the presence of atrazine, propazine, and simazine at a high rate, and that the regarded pesticides most likely do no inhibit the *h*AChE covalently [85]. Therefore, the protocol by which the *Homo sapiens* LD_50_ values were calculated, as well as all the performed molecular modelling studies of 2-chloro-1,3,5-triazine-based pesticides, were experimentally validated. 

## 3. Materials and Methods 

### 3.1. Materials

Acetylcholine chloride (CAS: 60-31-1), acetylcholinesterase from human erythrocytes (CAS: 9000-81-1), and DTNB (5,5′-dithiobis(2-nitrobenzoic acid)) (CAS: 69-78-3), atrazine (CAS: 1912-24-9), propazine (CAS: 139-40-2), simazine (CAS: 122-34-9), and PBS (MDL: MFCD00131855) were purchased from Sigma Aldrich (St. Louis, Mo., USA).

### 3.2. The Generation of Pesticides Structures

The crystal structures of simazine [9], monocrotophos [10], dimethoate [11], and acetamiprid [12] were retrieved from The Cambridge Crystallographic Data Centre (CCDC) (CSD IDs: KUYXIM, ULEJIF, IPCPYB, HANBAA). The remaining pesticides structures were generated by drawing using the MacroModel version 8 software (Schrodinger, Cambridge, MA, USA) [75].

### 3.3. The Conformational Analysis

The conformational analyses were performed by using the MacroModel version 8 software [75] via the Monte Carlo/multiple minimum (MC/MM) approach [76]. The solute was described using five different potentials (MM3, AMBER94, MMFF, MMFFs, OPLSAA) [77,85,86,87,88]. The effect of solvent was incorporated into the MC/MM calculations using the generalized Born/surface area (GB/SA) continuum solvent model [89]. Cut-offs of 12.0 Å and 7.0 Å were employed for electrostatic and van der Waals non-bonded interactions, respectively [90]. The MC simulation involved 104 steps at 310.15 K, applied to all the rotatable bonds, with random torsional rotations of up to ±180°. This was combined with 103 steps of energy minimization. All the conformational calculations were performed using the MacroModel 8.0 and BatchMin suite of programs [91].

### 3.4. The Mus musculus and Homo sapiens AChE Sequences Alignment

The alignment of *m*AChE and *h*AChE sequences retrieved from the UniProt database (entries P221836 and P22303, respectively, Figure S1) was performed by means of ClustalW module [92] using the standard setup.

### 3.5. The Mus musculus and Homo sapiens AChE Complexes Preparation

To the best of our knowledge, there are no crystal structures containing co-crystallized structures of pesticides listed in Table 1, in complex with either *Mus musculus* or *Homo sapiens* AChE. Therefore, for the purpose of targeted pesticides binding mode definition into the active sites of *Mus musculus* and *Homo sapiens* AChEs, respectively, 59 acetylcholine esterase-inhibitor complexes (51 containing mice inhibitors and 8 saturated with human inhibitors) were collected from Protein Data Bank and submitted to structure-based alignment assessment protocols (see Tables S9 and S10). Initially, complexes were prepared for molecular modelling after being loaded into the UCSF Chimera v1.10.1 software [93] for Linux 64 bit architecture and visually inspected. The downloaded complexes were homodimers. For the experimental purposes preparation, complexes were initially arbitrarily superimposed using human crystal under the code name **4EY5 [94]** as a template (the best-resolved complex with the resolution of 2.3 Å) using the Matchmaker module and then separated in chains using the command line implementation of the Chimera split command. Upon the thorough examination, the B chain of each complex was retained for further manipulation. Compared to chains A containing complexes, those with chains B were more complete by means of inhibitors presence. Inhibitors were extracted from each chain B complexes after which either proteins or ligands were improved by assigning the hydrogen atoms at pH of 7.4 and Amber parameters [95] using Antechamber semi-empirical QM method. Complexes were the regenerated and energy minimized as follows: through the leap module they were solvated with water molecules (TIP3P model, SOLVATEOCT Leap command) in a box extending 10 Å in all directions, neutralized with either Na^+^ or Cl^−^ ions, and refined by a single point minimization using the Sander module with maximum 1000 steps of the steepest-descent energy minimization followed by maximum 4000 steps of conjugate-gradient energy minimization, with a non-bonded cutoff of 5 Å. Minimized complexes were re-aligned (**4EY5** as a template), after which all of the ligands were extracted to compose the SB aligned training set ready to be utilized for the subsequent structure-based alignment assessment. 

### 3.6. The Structure-Based Alignment Assessment

The SB alignment assessment for either *Mus musculus* or *Homo sapiens* AChE inhibitors was performed by means of the four step standard protocol [78,96], by means of either AutoDock [79], AutoDock Vina [80], and DOCK [81] docking algorithms/scoring functions.

Experimental conformation re-docking (ECRD): a procedure in which the experimental conformations (EC) are flexibly docked back into the corresponding protein, evaluating the program for its ability to reproduce the observed bound conformations.

Randomized conformation re-docking (RCRD): similar assessment to ECRD with a difference that the active site of the protein is virtually occupied by conformations initially obtained from computational random optimization of corresponding co-crystallized molecules coordinates and positions. Here the programs are evaluated for their ability to find the experimental pose starting by a randomized minimized conformation.

Experimental conformation cross-docking (ECCD): comparable to ECRD, but the molecular docking was performed on all the training set proteins except for the corresponding natives. Here the programs are evaluated to find the binding mode of ligand in a similar but different from the native complex active site at the same time mimicking discrete protein flexibility.

Randomized conformation cross-docking (RCCD): same as the ECCD but using RCs as starting docking conformations. This is the highest level of difficulty since the program is demanded to dock any given molecule into an ensemble of protein conformations not containing the native one. The outcome is considered as the most important ability of docking program as the most accurate scoring function is applied to any test set molecules of which the experimental binding mode is unknown. The related docking accuracy (DA) [97] is a direct function of the program’s probability to find a correct binding mode for an active molecule.

#### 3.6.1. The AutoDock Settings

The prepared protein structures were imported into AutoDockTools graphical user interface. Nonpolar hydrogen atoms were removed while Gasteiger charges and solvent parameters were added. All of the tested compounds were used as ligands for docking. The rigid root and rotatable bonds were defined using AutoDockTools. The docking was performed with AutoDock 4.2. The xyz coordinates (in Ångströms) of the cuboid grid box used for the computation were: for mouse inhibitors Xmin/Xmax = 1.420/31.800, Ymin/Ymax = 108.000/152.500, Zmin/Zmax = 8.200/43.200; for human inhibitors Xmin/Xmax = 3.200/29.500, Ymin/Ymax = 109.200/153.800, Zmin/Zmax = 8.800/42.100, with a purpose to embrace all the minimized inhibitors spanning 10 Å in all three dimensions. The Lamarckian Genetic Algorithm was used to generate orientations or conformations of ligands within the binding site. The procedure of the global optimization started with a sample of 200 randomly positioned individuals, a maximum of 1.0 × 10^6^ energy evaluations and a maximum of 27,000 generations. A total of 100 runs were performed with RMS Cluster Tolerance of 0.5 Å.

#### 3.6.2. The Autodock Vina Settings

The docking simulations were carried out in the same grid space with an energy range of 10 kcal/mol and exhaustiveness of 100 with RMS Cluster Tolerance of 0.5 Å. The output comprised 20 different conformations for every receptor considered. 

#### 3.6.3. The DOCK6 Settings

During the docking simulations with DOCK program, the proteins were rigid while the inhibitors flexible and subjected to an energy minimization process. The solvent accessible surface of each enzyme without hydrogen atoms was calculated using the DMS program [98], using a probe radius of 1.4 Å. The orientation of ligands was described using the SPHGEN module and by sphere selector. A box around the binding site is constructed with the accessory program SHOWBOX. The steric and electrostatic environment of the pocket was evaluated with the program Grid using a 0.3 Å of grid spacing. Selected spheres were within 8 Å from ligand heavy atoms of the crystal structure and for computing the energy grids an 8 Å box margin and 6–9 VDW exponents were used.

### 3.7. The Ligand-Based and Structure-Based Alignment Accuracy

The alignment fitness for LB or SB approach was quantified by evaluating the RMSD values. The alignment accuracy was initially quantified after reproducing the known crystal structures of simazine [9], monocrotophos [10], dimethoate [11], and acetamiprid [12], with the available force fields, to obtain the conformational alignment accuracy (CAA). Following, the superposition of pesticides structures obtained after the conformational analysis was performed with the purpose to compare the force fields performances, i.e., the force filed-dependent alignment accuracy, FFDAA. Finally, superimposition of docking poses provided the docking accuracy, DA. CAA, FFDAA or DA can be used to test how the conformational alignment can predict the ligand pose as close as possible to the experimentally observed one [97]. The alignment accuracy values can be calculated by the following Equation (7):

(7) $$xA=f_{rmsd,}\;\leq a+0.5\left(f_{rmsd}\;\leq b-f_{rmsd}\;\leq a\right)$$

In particular: *xA* = CAA or FFDAA, in the case of LB alignment accuracy, whereas the *xA* = DA, in the case of SB accuracy. The *f_rmsd_* ≤ *a* and *f_rmsd_* ≤ *b* are fractions of aligned ligands showing an RMSD value less than or equal to 2 Å (*a* coefficient) and 3 Å (*b* coefficient), respectively. The widely accepted standard is that the correctly aligned conformations are those with RMSD less than 2 Å on all the heavy atoms between the generated and crystallographic structure, when considering the CAA and FFDAA, while the correctly docked structures are those with RMSD less than 2 Å on all the heavy atoms between docked and co-crystallized ligand, when considering the DA. Structures with the RMSD values larger than 4 Å were considered incorrectly aligned/docked. Structures with RMSD between 2 and 3 were considered partially aligned/docked, whereas those with RMSD higher than 3 and were misaligned/mis-docked and thus not considered in the FFDAA/DA calculation.

### 3.8. The Structure-Based Alignment of Pesticides

The molecular docking of pesticides within either *m*AChE and *h*AChE was conducted by the AutoDock Vina [80]. The molecular docking studies were carried out in the cuboid grid box with the following xyz coordinates (in Ångströms): for *m*AChE Xmin/Xmax = 1.420/31.800, Ymin/Ymax = 108.000/152.500, Zmin/Zmax = 8.200/43.200; *h*AChE Xmin/Xmax = 3.200/29.500, Ymin/Ymax = 109.200/153.800, Zmin/Zmax = 8.800/42.100, with the energy range of 10 kcal/mol and exhaustiveness of 100 with RMS Cluster Tolerance of 0.5 Å. The output comprised 20 different conformations for every receptor considered.

### 3.9. The Pesticide-AChE Complexes Molecular Dynamics Simulations

The pesticide-AChE complexes formed after molecular docking were used to perform the explicit solvent molecular dynamics (MD) simulations. The parallelized Desmond Molecular Dynamics System [99] and the associated analysis tools, available within the Schrödinger suite 2009 [75] were used for this purpose. The models were prepared using the ‘system builder’ utility. The MMFF force field parameters were assigned to all the simulation systems. Each inhibitors-enzyme complex was placed in the centre of an orthorhombic box ensuring 10 Å solvent buffers between the solute and the boundary of the simulation box in each direction. The TIP3P model was used to describe the water molecules. Moreover, Na^+^ and Cl^−^ ions were added at a physiological concentration of 0.15 M. The model systems were relaxed to 0.5 Å using the single step minimization protocol and were subjected to the production phase, with the NPT ensemble and the periodic boundary conditions for 1.2 ns. The pressure control was used to maintain the pressure of the cell (1.013 bars) with the isotropic coupling method. The Nose-Hoover thermostat method was used to control the temperature at 310.15 K. The Heavy atom-hydrogen covalent bonds were constrained using the SHAKE algorithm which allowed 2 fs integration step to be used. The integration of the motion equation using the RESPA multiple time step scheme was used in the calculation. The Long-range electrostatic forces were obtained using the smooth Particle-Mesh Ewald (PME) method. In order to calculate the short-range electrostatics and the Lennard-Jones interaction, the cut-off distance was set to 9.0 Å, and the trajectories and the energies were recorded at every 4.8 ps. The simulation quality analysis tool was used to analyse the trajectories; RMSD and RMSF values, the hydrogen bond distances, the angles, and the van der Waals interactions were obtained over the simulation trajectories.

### 3.10. The MM-GBSA Calculations and Free Energy Decomposition

The binding free energy [100] of each system was calculated using the MM-GBSA calculations according to the following Equation (8):

(8) $$\;\;\;\;\;\;\;\;\;\Delta G_{\mathrm{bind}\;}=\Delta E_{\mathrm{ele}}+\Delta E_{\mathrm{vdW}}+\Delta G_{\mathrm{solv}}+T\Delta S,\;$$

where Δ*E*_ele_ corresponds to the electrostatic energy difference between the receptor-ligand bound and the calculated unbound states using the OPLS_2005 force field, Δ*E*_vdW_ corresponds to the van der Waals contribution, while Δ*G*_solv_ is the corresponding solvation free energy contribution of the binding which was calculated using the GB/SA continuum model. The Embrace package implemented in MacroModel was used for the Δ*E*_ele_, Δ*E*_vdW_, and Δ*G*_solv_ calculations. The entropy change Δ*S* was calculated using the Rigid Rotor Harmonic Oscillator (RRHO) calculations. Having used this algorithm, the change in vibrational, rotational, and translational (VRT) entropy of the ligands on binding was estimated. For the RRHO calculations, the representative complexes were pre-minimized using the Desmond with the explicit solvent retained; a 2000 steps LBFGS minimization (first 10 steps steepest descent) with the residues beyond 15 Å of ligands restrained and a convergence criterion of 0.05 kcal mol^−1^ Å^−1^ was used.

### 3.11. The Quantum Mechanical Mechanistic Studies

The DFT-based calculations were performed on the MD-generated pesticide-*h*AChE complexes on three different levels. The first level included the extraction of hydrogen bond-forming geometries of MD equilibrated pesticide-*h*Thr83, pesticide-*h*Tyr124, pesticide-*h*Tyr341, and pesticide-*h*His447 sub-complexes; subsequently, the extracted geometries were QM optimized to locate the transition states (TSs) and/or intermediary states (ISs), by which the protons transfers occur on a particular segment of a biochemical reaction. The second level of the calculation attempted to find the identical TSs and/or ISs following the manual adjustment of the TS-generating hydrogen atom position, between the pesticide and the regarded AChE residue, within the pesticide-*h*Thr83, pesticide-*h*Tyr124, pesticide-*h*Tyr341, and pesticide-*h*His447 geometries. Each of the reaction pathways assumed the contemplation by means of Intrinsic Reaction Coordinate (IRC) analysis. As water molecules are not involved in HBs generation, they were all discarded from the system. The initiation or the manual setup of geometries was performed by means of GaussView6 package [101] following which the B3LYP method, as implemented in Gaussian 09 (Gaussian Inc., Wallingford, CT, USA) [102] was used to explore the geometry of the reactants, transition states, intermediary states and products. The single point calculations were carried out on all the geometries at the B3LYP/6-31G* level of theory [82]. The saddle points and transition states were quantified by means of frequency calculations.

### 3.12. The Assay of the AChE Activity 

The AChE catalytic activity was measured at 22 °C by the Ellman method [103]. The assay mixture contained the 0.25 mM ACh, 0.25 mM DTNB, and 50 mM sodium phosphate buffer. The remaining assay conditions have been reported previously. For the selection of a suitable concentration of AChE, at which the relationship between the initial velocity and the total enzyme concentration should be linear, 0.20–3.20 mg/mL of AChE was assayed in vitro from 2 to 20 min under the same conditions of the temperature and the pH. The rate of change of the substrate cleavage, determined by measuring the absorbance of the reaction product at a wavelength of 412 nm, was performed at different time intervals. The product was calculated for each [AChE] at 4-min incubation time intervals. To study the effect of atrazine, simazine, or propazine on AChE substrate cleavage, the enzyme was preincubated with each pesticide at different concentrations ranging 0–6 μg/mL (concentrations corresponding to the LD_50_ values) for 10 min prior to the addition of the substrate.

## 4. Conclusions

The present report describes the application of the molecular modelling techniques to shed light on the pharmacology of the commonly used pesticides atrazine, simazine, propazine, carbofuran, monocrotophos, dimethoate, carbaryl, tebufenozide, imidacloprid, acetamiprid, diuron, monuron, and linuron as AChE inhibitors The pesticides’ putative pre-binding conformations in the inter-synaptic environment were predicted by means of the conformational analysis where the determined global minima values and the ability to reproduce the pesticides experimental conformations indicated MMFF as the best performing FF. Having known the pesticides pre-bound conformations, their acute toxicities were interrelated to the *E*_glob_min_ values through the QSAR studies: for the majority of pesticides, a clear inverse correlation could be observed between the *E*_glob_min_ values and the high acute toxicities. However, the chemometric analysis indicated that the *E*_glob_min_ values could not be used as proper indicators for high or low LD_50_ values against *Mus musculus* or *Homo sapiens*.

Therefore, the pesticides acute toxicity was further on regarded trough the pesticides bound conformations, upon the molecular docking and molecular dynamics-driven interactions with either *Mus musculus* or *Homo sapiens* AChE. The conducted studies confirmed the binding modes and the pharmacology of evaluated pesticides as reversible and reversible-like (i.e., future covalent) AChE inhibitors and the determined pesticides pharmacodynamics origin of acute toxicity. The superposed chemometric investigation revealed that for all the pesticides, a clear interrelationship could be observed between the *∆G*_binding_ values and high acute toxicity, indicating that the *∆G*_binding_ values can be used as indexes for high or low LD_50_ values against *Mus musculus*. Moreover, the obtained chemometric guideline, in the form of the QSAR model *pLD*_50_ = −0.7885∆*G*_binding_ − 2.44, is, by all means, suitable for the prognosis of LD_50_ values against *Homo sapiens* and can be further used as a universal tool for the acute toxicities administration of novel pesticides. Further studies which consider the inclusion of additional parameters and machine learning algorithm application are in due course in order to generate quantitative structure-activity relationships models able to include all considered pesticides in a comprehensive mathematical model. 

Nevertheless, it is interesting that the global minima or free energy of formation values may indicate the level of acute toxicity; the calculations may discard the potential new pesticides even before the actual application in agriculture. Therefore, the correlations between the pLD_50_ and *E*_glob_min_ or ∆*G*_binding_ are hereby presented for the very first time as an unprecedented way to study pesticides and to predict their acute toxicity.

Moreover, the subsequent structure-based quantum mechanical mechanistic studies for 2-chloro-1,3,5-triazine-based pesticides, as the least understood group by means of pharmacology, revealed pathways by which considered pesticides poison *Homo sapiens* AChE in a reversible fashion manner. To summarize, all the noticed interactions could further be used in the discovery of novel pesticides with the desirable lower acute toxicity against humans. It is important to emphasize that the conducted QM studies were performed to provide the structural basis for the pesticides’ human AChE toxicity, not the general toxicity. The pesticide toxicity is mainly carried out by means of mechanism; general toxicity is normally due to off target interactions or to active metabolites.

## Figures and Tables

**Table 1:** Acetylcholine and training set pesticides structures as well as their oral acute toxicities (LD_50_ values) against *Mus musculus* (upper values); training set pesticides calculated oral acute toxicities (LD_50_ values) against *Homo sapiens* (lower values, bold and italic) [32].

Pesticide	Structure	LD_50_ (mg/kg)	Ref.	Pesticide	Structure	LD_50_ (mg/kg)	Ref.
**atrazine**	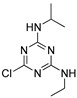	0.850 ***0.0689***	[20]	**tebufenozide**	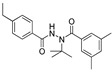	5000 ***405.40***	[29]
**propazine**	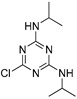	3.18 ***0.258***	[21]	**imidacloprid**	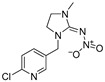	131 ***10.62***	[30]
**simazine**	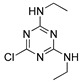	5 ***0.405***	[24]	**acetamiprid**	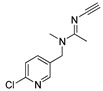	18 ***14.92***	[31]
**carbofuran**	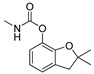	2 ***0.162***	[25]	**diuron**	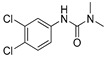	500 ***40.54***	[22]
**monocrotophos**	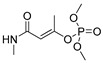	14 ***1.135***	[26]	**monuron**	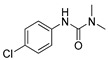	1700 ***437.84***	[23]
**dimethoate**	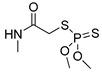	60 ***4.86***	[27]	**linuron**	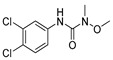	2400 ***194.59***	[19]
**carbaryl**	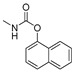	100 ***8.11***	[28]	**acetylcholine**	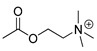		

**Table 2:** Test set pesticides structures and their oral acute toxicities (LD_50_ values) against *Mus musculus* (upper values); test set pesticides calculated oral acute toxicities (LD_50_ values) against *Homo sapiens* (lower values, bold and italic) [32].

Pesticide	Structure	LD_50_ (mg/kg)	Ref.	Pesticide	Structure	LD_50_ (mg/kg)	Ref.
**azamethiphos**	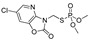	1040 ***84.324***	[44]	**phosmet**	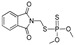	113 ***9.162***	[53]
**azinphos-methyl**	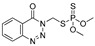	7 ***0.567***	[54]	**TCVP**	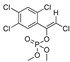	465 ***37.702***	[55]
**chlorpyrifos**	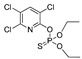	2000 ***162.162***	[56]	**terbufos**	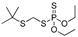	1.6 ***0.129***	[57]
**DDVP**	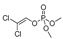	17 ***1.378***	[58]	**methiocarb**	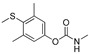	350 ***28.378***	[59]
**diazinon**	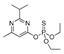	66 ***5.351***	[60]	**methomyl**	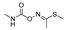	12 ***0.972***	[61]
**fenitrothion**	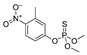	50 ***40.540***	[62]	**oxamyl**	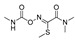	5.4 ***0.437***	[63]
**glyphosate**	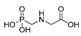	5000 ***405.405***	[64]	**DDT**	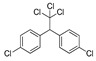	113 ***9.162***	[14]
**malathion**	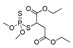	290 ***23.513***	[65]	**2,4-D**	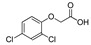	639 ***51.810***	[66]
**methyl parathion**	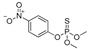	18 ***1.459***	[67]	**dicamba**	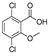	75 ***61.378***	[68]
**naled (dibrom)**	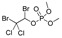	160 ***12.972***	[69]	**DEET**	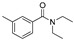	1.95 ***0.158***	[70]
**parathion**	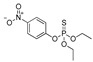	2 ***0.162***	[71]	**sulfoxaflor**	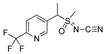	750 ***60.810***	[72]

**Table 3:** Training set pesticides with the known crystal structures: chemical structures, conformational analysis, superposition of the experimental conformation and generated global minima using the best performing force fields.

Pesticide	FF ^a^	*E*_glob_min_^b^	NGMS ^c^	NF ^d^	Pesticide	CAA ^f^
		(kJ/mol)			Alignment ^e^	RMSD (Å) ^g^
**simazine**	MM3	NA ^h^	NA	NA	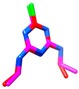	EC/MMFF
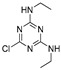	AMBER94	NA	NA	NA		0.667
	MMFF	−1022.18	136	2782		
	MMFFs	NA	NA	NA		
	OPLSAA	−793.61	158	2306		
**monocrotophos**	MM3	NA	NA	NA	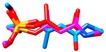	EC/MMFF
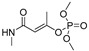	AMBER94	NA	NA	NA		0.620
	MMFF	−307.36	241	62		EC/MMFFs
	MMFFs	−310.26	218	61		3.521
	OPLSAA	NA	NA	NA		
**dimethoate**	MM3	NA	NA	NA	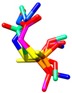	EC/MMFF
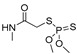	AMBER94	NA	NA	NA		0.148
	MMFF	−383.68	340	44		EC/MMFFs
	MMFFs	−386.71	215	40		3.227
	OPLSAA	−228.66	998	15		EC/OPLSAA
						2.746
**acetamiprid**	MM3	NA	NA	NA	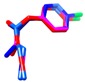	EC/MMFF
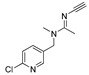	AMBER94	NA	NA	NA		0.783
	MMFF	−31.45	1916	14		EC/MMFFs
	MMFFs	−31.29	1816	6		0.823
	OPLSAA	NA	NA	NA		

^a^ Force field; ^b^ Energy of the global minimum; ^c^ Number of times that a single global minimum structure was found in 10,000 processed structures; ^d^ Number of families i.e., different conformations found in 10,000 processed structures; ^e^ Red—experimental conformation, Violet—MMFF conformation, Blue—MMFFs conformation, Green—OPLSAA conformation; ^f^ Conformation dependent alignment accuracy; ^g^ RMSD measured between the heavy atoms of pesticides of experimental and best performing force field conformations; ^h^ Not available.

**Table 4:** Training set pesticides of the highest toxicity with the unknown crystal structures: chemical structures, conformational analysis, superposition of the experimental conformation and generated global minima using the best performing force fields.

Pesticide	FF ^a^	*E_glob_min_* ^b^	NGMS ^c^	NF ^d^	Pesticide	FFDAA ^f^
		(kJ/mol)			Alignment ^e^	RMSD (Å) ^g^
**atrazine**	MM3	−1161.48	445	23	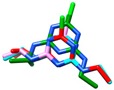	MM3/MMFF
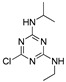	AMBER94	NA ^h^	NA	NA		0.892
	MMFF	−1007.32	147	62		MM3/MMFFs
	MMFFs	−992.63	509	20		0.931
	OPLSAA	−783.57	364	19		MMFF/MMFF_S_
						0.343
**carbofuran**	MM3	29.60	3617	2	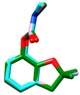	MMFF/MMFF_S_
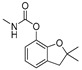	AMBER94	NA	NA	NA		0.075
	MMFF	20.30	3307	2		MMFF/OPLSAA
	MMFFs	15.92	3384	2		0.216
	OPLSAA	73.27	5621	75		MMFFs/OPLSAA
						0.239

^a^ Force field; ^b^ Energy of the global minimum; ^c^ Number of times that a single global minimum structure was found in 10,000 processed structures; ^d^ Number of families i.e., different conformations found in 10,000 processed structures; ^e^ Red—experimental conformation, Violet—MMFF conformation, Blue—MMFFs conformation, Green—OPLSAA conformation; ^f^ Force field dependent alignment accuracy; ^g^ RMSD measured between the heavy atoms of pesticides experimental and best performing force field conformations; ^h^ Not available.

## Supplementary Materials

The Supplementary Materials are available online.
