# Supplementary material for: The Targeted Pesticides as Acetylcholinesterase Inhibitors: Comprehensive Cross-Organism Molecular Modelling Studies Performed to Anticipate the Pharmacology of Harmfulness to Humans In Vitro
Source: Molecules. 2018 Aug 30;23(9):2192. doi: 10.3390/molecules23092192 (PMC6225315; doi:10.3390/molecules23092192)
Supplement: Supplementary file 1 [file molecules-23-02192-s001.pdf]

molecules

MDPI

Supplementary material

The targeted pesticides as Acetylcholinesterase inhibitors: comprehensive cross-organism molecular modelling studies performed to anticipate the pharmacology of harmfulness to humans *in vitro*

Milan Mladenović[1,*], Biljana B. Arsić[2,3], Nevena Stanković[1], Nezrina Mihović[1], Rino Ragno[4,5], Andrew Regan[6], Jelena S. Milićević[7], Tatjana M. Trtić-Petrović[7], Ružica Micić[8]

1 Kragujevac Center for Computational Biochemistry, Faculty of Science, University of Kragujevac, Radoja Domanovića 12, 34000 Kragujevac, P.O. Box 60, Republic of Serbia
2 Department of Mathematics, Faculty of Sciences and Mathematics, University of Niš, Višegradska 33, 18000 Niš, Republic of Serbia
3 Division of Pharmacy and Optometry, University of Manchester, Oxford Road, M13 9PT, Manchester, United Kingdom
4 Rome Center for Molecular Design, Department of Drug Chemistry and Technologies, Faculty of Pharmacy and Medicine, Sapienza Rome University, P.le A. Moro 5, 00185, Rome, Italy
5 Alchemical Dynamics srl 00125 Rome, Italy
6 School of Chemistry, University of Manchester, Oxford road, M13 9PL, Manchester, United Kingdom
7 Vinča Institute of Nuclear Sciences, University of Belgrade, PO Box 522, 11001 Belgrade, Republic of Serbia
8 Faculty of Sciences and Mathematics, University of Priština, Lole Ribara 29, 38220 Kosovska Mitrovica, Republic of Serbia
* Correspondence: mmladenovic@kg.ac.rs; Tel.: +381-34-336-223, ext. 255

CONTENTS

Figure S1. The sequence alignment between the *Mus musculus* and *Homo sapiens* AChE.

Figure S2. Vina-based The SB alignment assessment of (**a**) co-crystallized *m*AChE inhibitor (PDB ID: 4A16), EC pink, ECRD yellow, RCRD green, ECCD black, RCCD red, and (**b**) co-crystallized *h*AChE inhibitor (PDB ID: 4BDT), EC pink, ECRD yellow, RCRD green, ECCD black, RCCD red.

Figure S3. The SB alignment of propazine (**a**), carbaryl (**b**), tebufenozide (**c**), and acetamiprid (**d**) into the *m*AChE active site. The enzyme ribbons are presented in blue, active site amino acids are depicted in white. For the clarity purpose, hydrogen atoms are omitted from presentation.

Figure S4. The SB alignment of diuron (**a**), monuron (**b**), and linuron (**c**), into the *m*AChE active site. The enzyme ribbons are presented in blue, active site amino acids are depicted in white. For the clarity purpose, hydrogen atoms are omitted from presentation.

Figure S5. The SB alignment of propazine (**a**), carbaryl (**b**), tebufenozide (**c**), and acetamiprid (**d**) into the *h*AChE active site. The enzyme ribbons are presented in blue, active site amino acids are depicted in white. For the clarity purpose, hydrogen atoms are omitted from presentation.

Figure S6. The SB alignment of diuron (**a**), monuron (**b**), and linuron (**c**), into the *h*AChE active site. The enzyme ribbons are presented in blue, active site amino acids are depicted in white. For the clarity purpose, hydrogen atoms are omitted from presentation.

Figure S7. RMSDs (**a**), RMSFs (**b**), and Interaction diagrams (**c**) of *m*AChE in complex with ACh. The green bars present hydrogen bonds, the the purple bars show hydrophobic interactions, the pink bars show ionic interaction, the the blue bars present water bridges.

Figure S8. RMSDs (**a**), RMSFs (**b**), and Interaction diagrams (**c**) of *h*AChE in complex with ACh. The green bars present hydrogen bonds, the purple bars show hydrophobic interactions, the pink bars show ionic interaction, the blue bars present water bridges.

Figure S9. RMSDs (**a**), RMSFs (**b**), and Interaction diagrams (**c**) of *m*AChE in complex with atrazine. The green bars present hydrogen bonds, the purple bars show hydrophobic interactions, the pink bars show ionic interaction, the blue bars present water bridges.

Figure S10. RMSDs (**a**), RMSFs (**b**), and Interaction diagrams (**c**) of *h*AChE in complex with atrazine. The green bars present hydrogen bonds, the purple bars show hydrophobic interactions, the pink bars show ionic interaction, the blue bars present water bridges.

Figure S11. RMSDs (**a**), RMSFs (**b**), and Interaction diagrams (**c**) of *m*AChE in complex with propazine. The green bars present hydrogen bonds, the purple bars show hydrophobic interactions, the pink bars show ionic interaction, the blue bars present water bridges.

Figure S12. RMSDs (**a**), RMSFs (**b**), and Interaction diagrams (**c**) of *h*AChE in complex with propazine. The green bars present hydrogen bonds, the purple bars show hydrophobic interactions, the pink bars show ionic interaction, the blue bars present water bridges.

Figure S13. RMSDs (**a**), RMSFs (**b**), and Interaction diagrams (**c**) of *m*AChE in complex with simazine. The green bars present hydrogen bonds, the purple bars show hydrophobic interactions, the pink bars show ionic interaction, the blue bars present water bridges.

Figure S14. RMSDs (**a**), RMSFs (**b**), and Interaction diagrams (**c**) of *h*AChE in complex with simazine. The green bars present hydrogen bonds, the purple bars show hydrophobic interactions, the pink bars show ionic interaction, the blue bars present water bridges.

Figure S15. RMSDs (**a**), RMSFs (**b**), and Interaction diagrams (**c**) of *m*AChE in complex with carbofuran. The green bars present hydrogen bonds, the purple bars show hydrophobic interactions, the pink bars show ionic interaction, the blue bars present water bridges.

Figure S16. RMSDs (**a**), RMSFs (**b**), and Interaction diagrams (**c**) of *h*AChE in complex with carbofuran. The green bars present hydrogen bonds, the purple bars show hydrophobic interactions, the pink bars show ionic interaction, the blue bars present water bridges.

Figure S17. RMSDs (**a**), RMSFs (**b**), and Interaction diagrams (**c**) of *m*AChE in complex with monocrotophos. The green bars present hydrogen bonds, the purple bars show hydrophobic interactions, the pink bars show ionic interaction, the blue bars present water bridges.

Figure S18. RMSDs (**a**), RMSFs (**b**), and Interaction diagrams (**c**) of *h*AChE in complex with monocrotophos. The green bars present hydrogen bonds, the purple bars show hydrophobic interactions, the pink bars show ionic interaction, the blue bars present water bridges.

Figure S19. RMSDs (**a**), RMSFs (**b**), and Interaction diagrams (**c**) of *m*AChE in complex with dimethoate. The green bars present hydrogen bonds, the purple bars show hydrophobic interactions, the pink bars show ionic interaction, the blue bars present water bridges.

Figure S20. RMSDs (**a**), RMSFs (**b**), and Interaction diagrams (**c**) of *h*AChE in complex with dimethoate. The green bars present hydrogen bonds, the purple bars show hydrophobic interactions, the pink bars show ionic interaction, the blue bars present water bridges.

Figure S21. RMSDs (**a**), RMSFs (**b**), and Interaction diagrams (**c**) of *m*AChE in complex with carbaryl. The green bars present hydrogen bonds, the purple bars show hydrophobic interactions, the pink bars show ionic interaction, the blue bars present water bridges.

Figure S22. RMSDs (**a**), RMSFs (**b**), and Interaction diagrams (**c**) of *h*AChE in complex with carbaryl. The green bars present hydrogen bonds, the purple bars show hydrophobic interactions, the pink bars show ionic interaction, the blue bars present water bridges.

Figure S23. RMSDs (**a**), RMSFs (**b**), and Interaction diagrams (**c**) of *m*AChE in complex with tebufenozide. The green bars present hydrogen bonds, the purple bars show hydrophobic interactions, the pink bars show ionic interaction, the blue bars present water bridges.

Figure S24. RMSDs (**a**), RMSFs (**b**), and Interaction diagrams (**c**) of *h*AChE in complex with tebufenozide. The green bars present hydrogen bonds, the purple bars show hydrophobic interactions, the pink bars show ionic interaction, the blue bars present water bridges.

Figure S25. RMSDs (**a**), RMSFs (**b**), and Interaction diagrams (**c**) of *m*AChE in complex with imidacloprid. The green bars present hydrogen bonds, the purple bars show hydrophobic interactions, the pink bars show ionic interaction, the blue bars present water bridges.

Figure S26. RMSDs (**a**), RMSFs (**b**), Interaction diagrams (**c**) of *h*AChE in complex with imidacloprid. The green bars present hydrogen bonds, the purple bars show hydrophobic interactions, the pink bars show ionic interaction, the blue bars present water bridges.

Figure S27. RMSDs (**a**), RMSFs (**b**), and Interaction diagrams (**c**) of *m*AChE in complex with acetamiprid. The green bars present hydrogen bonds, the purple bars show hydrophobic interactions, the pink bars show ionic interaction, the blue bars present water bridges.

Figure S28. RMSDs (**a**), RMSFs (**b**), and Interaction diagrams (**c**) of *h*AChE in complex with acetamiprid. The green bars present hydrogen bonds, the purple bars show hydrophobic interactions, the pink bars show ionic interaction, the blue bars present water bridges.

Figure S29. RMSDs (**a**), RMSFs (**b**), and Interaction diagrams (**c**) of *m*AChE in complex with diuron. The green bars present hydrogen bonds, the purple bars show hydrophobic interactions, the pink bars show ionic interaction, the blue bars present water bridges.

Figure S30. RMSDs (**a**), RMSFs (**b**), and Interaction diagrams (**c**) of *h*AChE in complex with diuron. The green bars present hydrogen bonds, the purple bars show hydrophobic interactions, the pink bars show ionic interaction, the blue bars present water bridges.

Figure S31. RMSDs (**a**), RMSFs (**b**), and Interaction diagrams (**c**) of *m*AChE in complex with monuron. The green bars present hydrogen bonds, the purple bars show hydrophobic interactions, the pink bars show ionic interaction, the blue bars present water bridges.

Figure S32. RMSDs (**a**), RMSFs (**b**), and Interaction diagrams (**c**) of *h*AChE in complex with monuron. The green bars present hydrogen bonds, the purple bars show hydrophobic interactions, the pink bars show ionic interaction, the blue bars present water bridges.

Figure S33. RMSDs (**a**), RMSFs (**b**), and Interaction diagrams (**c**) of *m*AChE in complex with linuron. The green bars present hydrogen bonds, the purple bars show hydrophobic interactions, the pink bars show ionic interaction, the blue bars present water bridges.

Figure S34. RMSDs (**a**), RMSFs (**b**), and Interaction diagrams (**c**) of *m*AChE in complex with linuron. The green bars present hydrogen bonds, the purple bars show hydrophobic interactions, the pink bars show ionic interaction, the blue bars present water bridges.

Figure S35. The SB alignment of azamethiphos (**a**), azinphos-methyl (**b**), chlorpyrifos (**c**), DDVP (**d**), diazinon (**e**), and fenitrothion (**f**) into the *m*AChE active site. The enzyme ribbons are presented in blue, active site amino acids are depicted in white. For the clarity purpose, hydrogen atoms are omitted from presentation.

Figure S36. The SB alignment of glyphosate (**a**), malathion (**b**), methyl parathion (**c**), naled (dibrom) (**d**), parathion (**e**), and phosmet (**f**) into the *m*AChE active site. The enzyme ribbons are presented in blue, active site amino acids are depicted in white. For the clarity purpose, hydrogen atoms are omitted from presentation.

Figure S37. The SB alignment of TCVP (**a**), terbufos (**b**), methiocarb (**c**), methomyl (**d**), oxamyl (**e**), and DDT (**f**) into the *m*AChE active site. The enzyme ribbons are presented in blue, active site amino acids are depicted in white. For the clarity purpose, hydrogen atoms are omitted from presentation.

Figure S38. The SB alignment of 2,4-D (**a**), dicamba (**b**), DEET (**c**), and sulfoxaflor (**d**) into the *m*AChE active site. The enzyme ribbons are presented in blue, active site amino acids are depicted in white. For the clarity purpose, hydrogen atoms are omitted from presentation.

Figure S39. The SB alignment of azamethiphos (**a**), azinphos-methyl (**b**), chlorpyrifos (**c**), DDVP (**d**), diazinon (**e**), and fenitrothion (**f**) into the *h*AChE active site. The enzyme ribbons are presented in orange, active site amino acids are depicted in white. For the clarity purpose, hydrogen atoms are omitted from presentation.

Figure S40. The SB alignment of glyphosate (**a**), malathion (**b**), methyl parathion (**c**), naled (dibrom) (**d**), parathion (**e**), and phosmet (**f**) into the *h*AChE active site. The enzyme ribbons are presented in orange, active site amino acids are depicted in white. For the clarity purpose, hydrogen atoms are omitted from presentation.

Figure S41. The SB alignment of TCVP (**a**), terbufos (**b**), methiocarb (**c**), methomyl (**d**), oxamyl (**e**), and DDT (**f**) into the *h*AChE active site. The enzyme ribbons are presented in blue, active site amino acids are depicted in white. For the clarity purpose, hydrogen atoms are omitted from presentation.

Figure S42. The SB alignment of 2,4-D (**a**), dicamba (**b**), DEET (**c**), and sulfoxaflor (**d**) into the *h*AChE active site. The enzyme ribbons are presented in orange, active site amino acids are depicted in white. For the clarity purpose, hydrogen atoms are omitted from presentation.

Figure S43. The quantum chemical mechanism of *Homo sapiens* acetylcholinesterase inhibition by propazine. The extracted geometry of IS1 (a); TS1 (b); IS2 (c); TS2 (d).

Figure S44. The quantum chemical mechanism of *Homo sapiens* acetylcholinesterase inhibition by simazine. The extracted geometry of IS1 (a); TS1 (b); IS2 (c); TS2 (d).

Figure S45. Free energy profile for *Homo sapiens* acetylcholinesterase inhibition by propazine (**a**), and simazine (**b**) by means of B3LYP (6-31G*) QM simulations.

Table S1. Training set pesticides chemical structures, conformational analysis, superposition of generated global minima using various force fields.

Table S2. Training set pesticides chemical structures, conformational analysis, superposition of generated global minima using various force fields.

Table S3. Training set pesticides chemical structures, conformational analysis, superposition of generated global minima using various force fields.

Table S4. Test set pesticides chemical structures and obtained global minima using MMFF as the best performing force field.

Table S5. Linear regression parameters for QSAR model 2 (acute toxicity against *Mus musculus*).

Table S6. External validation of QSAR model 2 (acute toxicity against *Mus musculus*).

Table S7. Linear regression parameters for QSAR model 4 (acute toxicity against *Homo sapiens*).

Table S8. External validation of QSAR model 4 (acute toxicity against *Homo sapiens*).

Table S9. Structure-based alignment assessment for wild type *Mus musculus* AChE inhibitors.

Table S10. Structure-based alignment assessment for wild type *Homo sapiens* AChE inhibitors.

Table S11. Binding free energies and individual energy terms of *Mus Musculus* (upper part) and *Homo sapiens* (lower part) AChE in complex with acetylcholine and various targeted pesticides inhibitors.

Supplementary material references (SRs)

```
sp|P22303|ACES_HUMAN      MRPPQCLLHTPSLASPLLLLLLWLLGGGVGAEGREDAELLVTVRGGRLRGIRLKTPGGPV
sp|P21836|ACES_MOUSE      MRPPWYPLHTPSLAFPLLFLLLSLLGGGARAEGREDPQLLVRVRGGQLRGIRLKAPGGPV
                          ****   ******* ***:*** *****. ****** :*** ****:*******:*****

sp|P22303|ACES_HUMAN      SAFLGIPFAEPPMGPRRFLPPEPKQPWSGVVDATTFQSVCYQYVDTLYPGFEGTEMWNPN
sp|P21836|ACES_MOUSE      SAFLGIPFAEPPVGSRRFMPPEPKRPWSGVLDATTFQNVCYQYVDTLYPGFEGTEMWNPN
                          ************:* ***:*****:*****:******.**********************

sp|P22303|ACES_HUMAN      RELSEDCLYLNVWTPYPRPTSPTPVLVWIYGGGFYSGASSLDVYDGRFLVQAERTVLVSM
sp|P21836|ACES_MOUSE      RELSEDCLYLNVWTPYPRPASPTPVLIWIYGGGFYSGAASLDVYDGRFLAQVEGAVLVSM
                          *******************:******:***********:**********.*.* :*****

sp|P22303|ACES_HUMAN      NYRVGAFGFLALPGSREAPGNVGLLDQRLALQWVQENVAAFGGDPTSVTLFGESAGAASV
sp|P21836|ACES_MOUSE      NYRVGTFGFLALPGSREAPGNVGLLDQRLALQWVQENIAAFGGDPMSVTLFGESAGAASV
                          *****:*******************************:******* **************

sp|P22303|ACES_HUMAN      GMHLLSPPSRGLFHRAVLQSGAPNGPWATVGMGEARRRATQLAHLVGCPPGGTGGNDTEL
sp|P21836|ACES_MOUSE      GMHILSLPSRSLFHRAVLQSGTPNGPWATVSAGEARRRATLLARLVGCPPGGAGGNDTEL
                          ***:** ***.**********:********. ******** **:********:*******

sp|P22303|ACES_HUMAN      VACLRTRPAQVLVNHEWHVLPQESVFRFSFVPVVDGDFLSDTPEALINAGDFHGLQVLVG
sp|P21836|ACES_MOUSE      IACLRTRPAQDLVDHEWHVLPQESIFRFSFVPVVDGDFLSDTPEALINTGDFQDLQVLVG
                          :********* **:**********:***********************:***:.******

sp|P22303|ACES_HUMAN      VVKDEGSYFLVYGAPGFSKDNESLISRAEFLAGVRVGVPQVSDLAAEAVVLHYTDWLHPE
sp|P21836|ACES_MOUSE      VVKDEGSYFLVYGVPGFSKDNESLISRAQFLAGVRIGVPQASDLAAEAVVLHYTDWLHPE
                          *************.**************:******:****.*******************

sp|P22303|ACES_HUMAN      DPARLREALSDVVGDHNVVCPVAQLAGRLAAQGARVYAYVFEHRASTLSWPLWMGVPHGY
sp|P21836|ACES_MOUSE      DPTHLRDAMSAVVGDHNVVCPVAQLAGRLAAQGARVYAYIFEHRASTLTWPLWMGVPHGY
                          **::**:*:* ****************************:********:***********

sp|P22303|ACES_HUMAN      EIEFIFGIPLDPSRNYTAEEKIFAQRLMRYWANFARTGDPNEPRDPKAPQWPPYTAGAQQ
sp|P21836|ACES_MOUSE      EIEFIFGLPLDPSLNYTTEERIFAQRLMKYWTNFARTGDPNDPRDSKSPQWPPYTTAAQQ
                          *******:***** ***:**:*******:**:*********:*** *:*******:.***

sp|P22303|ACES_HUMAN      YVSLDLRPLEVRRGLRAQACAFWNRFLPKLLSATDTLDEAERQWKAEFHRWSSYMVHWKN
sp|P21836|ACES_MOUSE      YVSLNLKPLEVRRGLRAQTCAFWNRFLPKLLSATDTLDEAERQWKAEFHRWSSYMVHWKN
                          ****:*:***********:*****************************************

sp|P22303|ACES_HUMAN      QFDHYSKQDRCSDL
sp|P21836|ACES_MOUSE      QFDHYSKQERCSDL
                          ********:*****
```

Figure S1. The sequence alignment between the *Mus musculus* and *Homo sapiens* AChE.

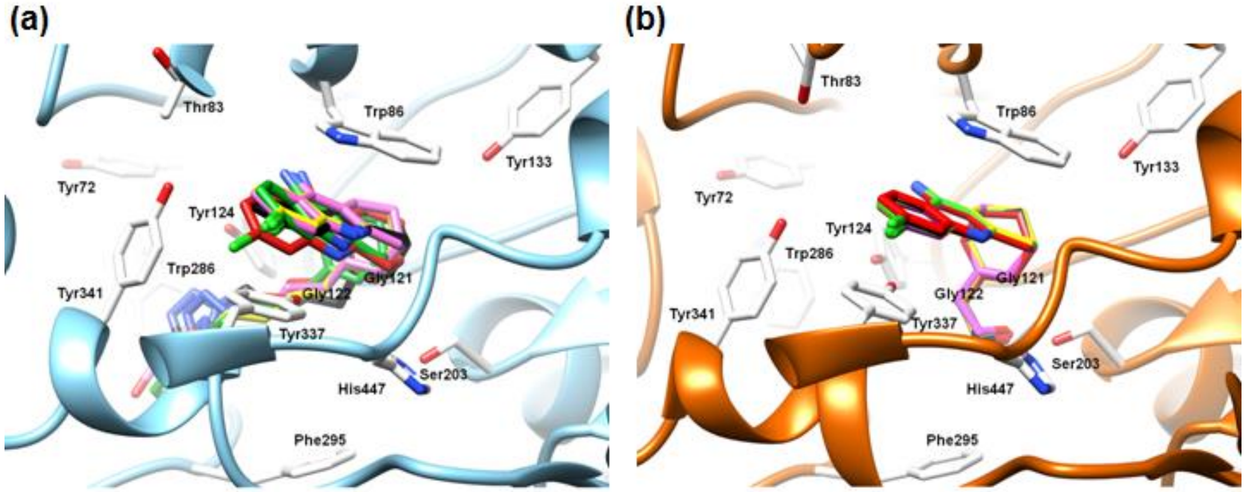

Figure S2. The Vina-based The SB alignment assessment of (**a**) co-crystallized *m*AChE inhibitor (PDB ID: 4A16), EC pink, ECRD yellow, RCRD green, ECCD black, RCCD red, and (**b**) co-crystallized *h*AChE inhibitor (PDB ID: 4BDT), EC pink, ECRD yellow, RCRD green, ECCD black, RCCD red.

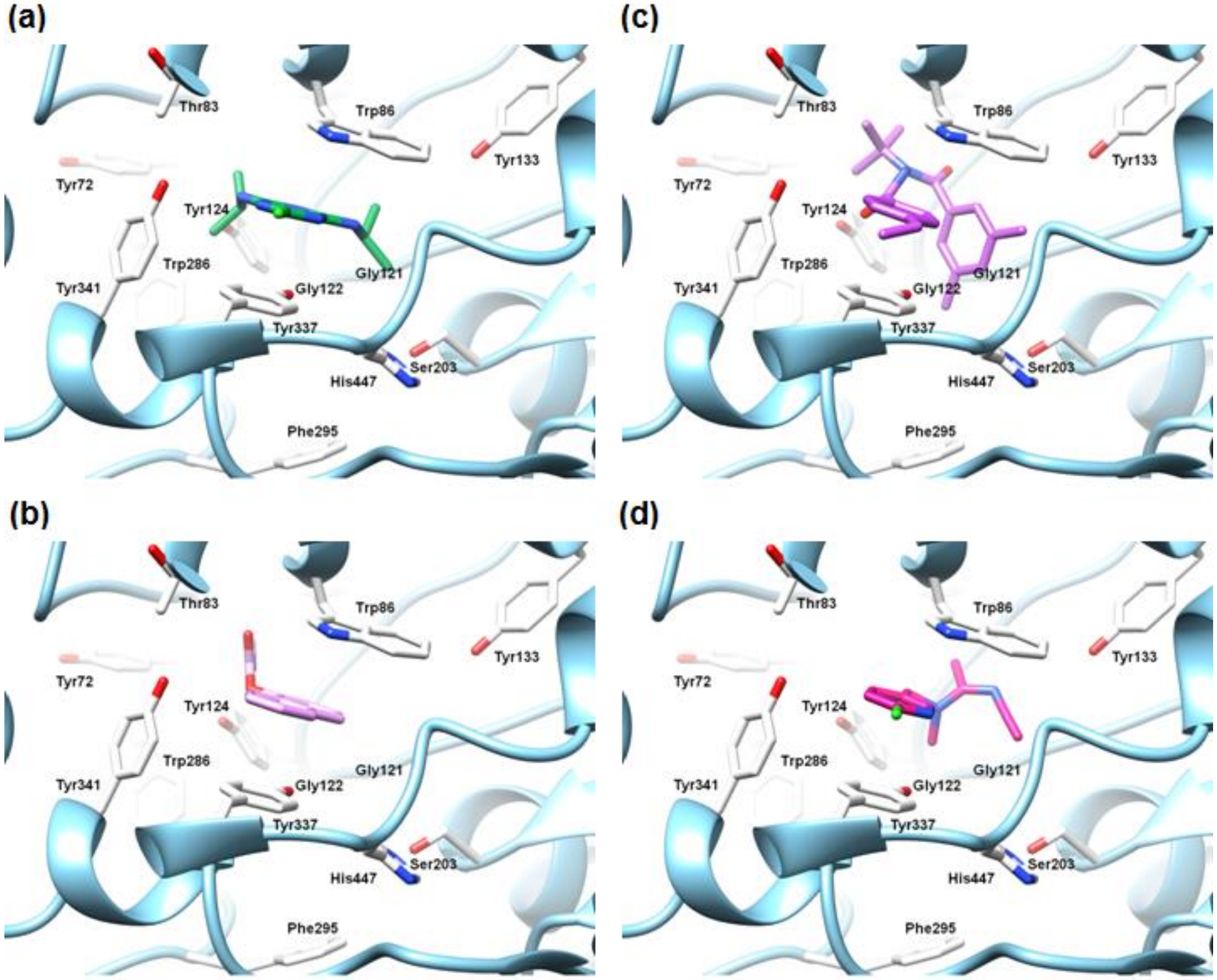

Figure S3. The The SB alignment of propazine (**a**), carbaryl (**b**), tebufenozide (**c**), and acetamiprid (**d**) into the *m*AChE active site. The The enzyme ribbons are presented in blue, active site amino acids are depicted in white. For the clarity purpose, hydrogen atoms are omitted from presentation.

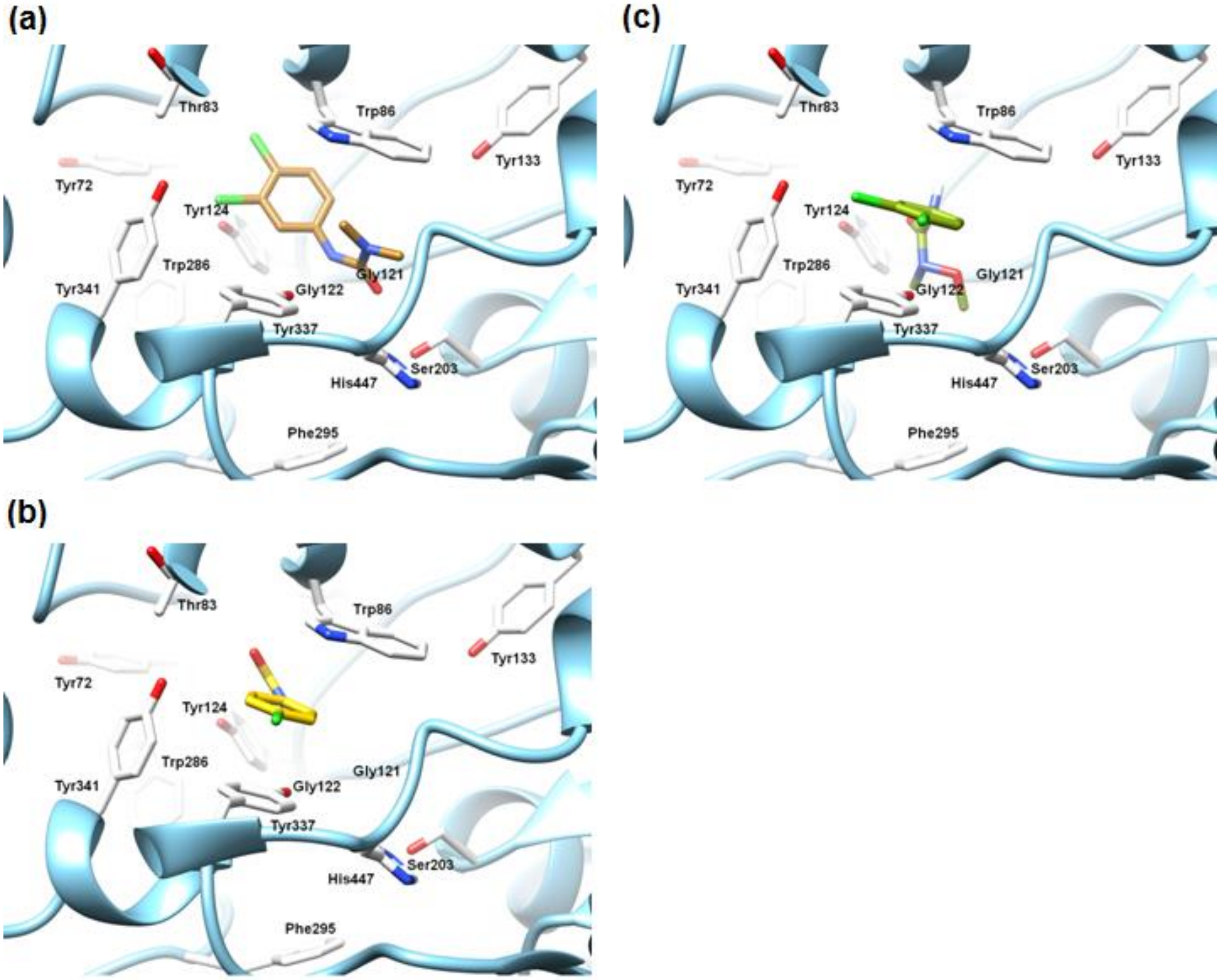

Figure S4. The The SB alignment of diuron (**a**), monuron (**b**), and linuron (**c**) into the *m*AChE active site. The The enzyme ribbons are presented in blue, active site amino acids are depicted in white. For the clarity purpose, hydrogen atoms are omitted from presentation.

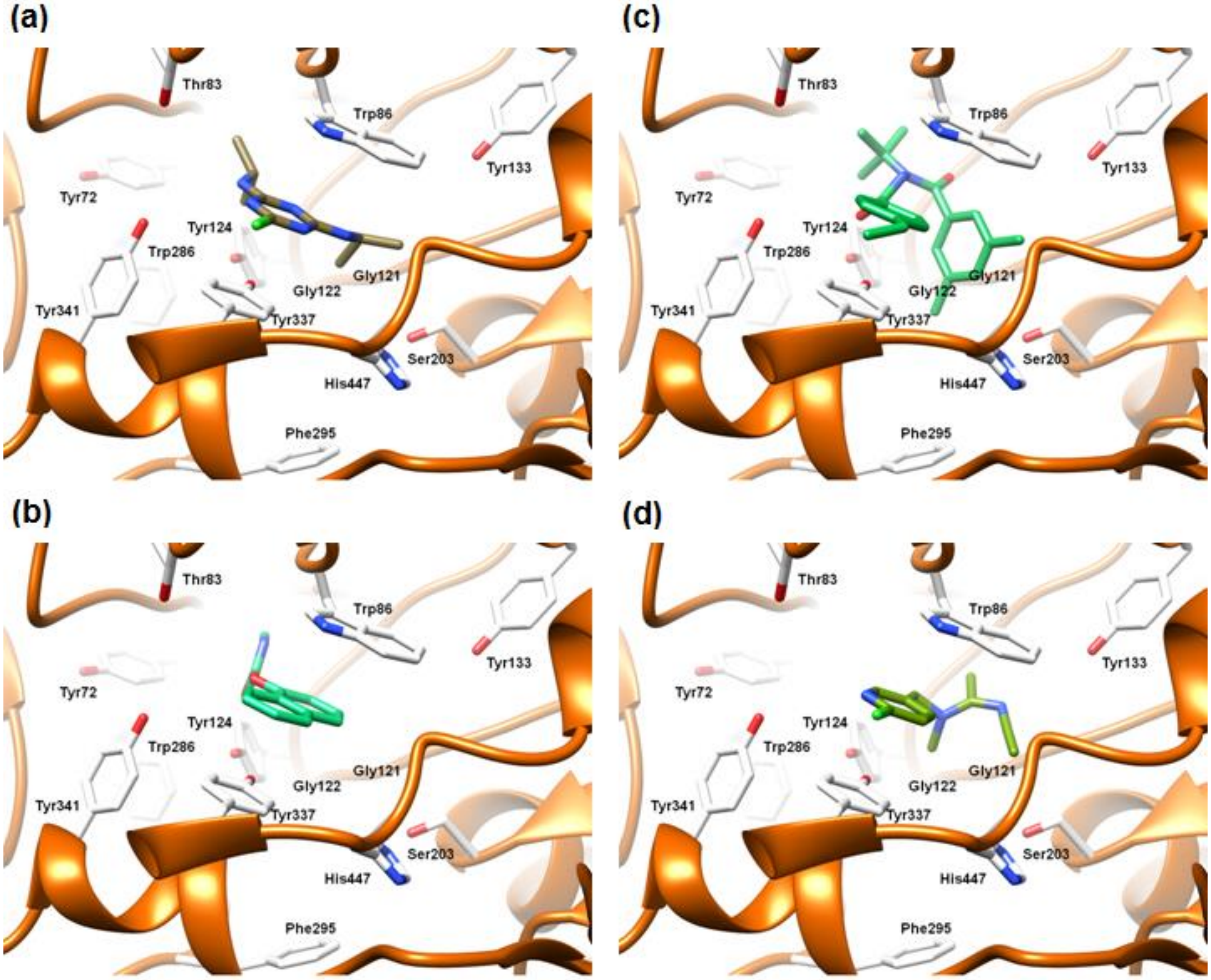

Figure S5. The The SB alignment of propazine (**a**), carbaryl (**b**), tebufenozide (**c**), and acetamiprid (**d**) into the *h*AChE active site. The The enzyme ribbons are presented in blue, active site amino acids are depicted in white. For the clarity purpose, hydrogen atoms are omitted from presentation.

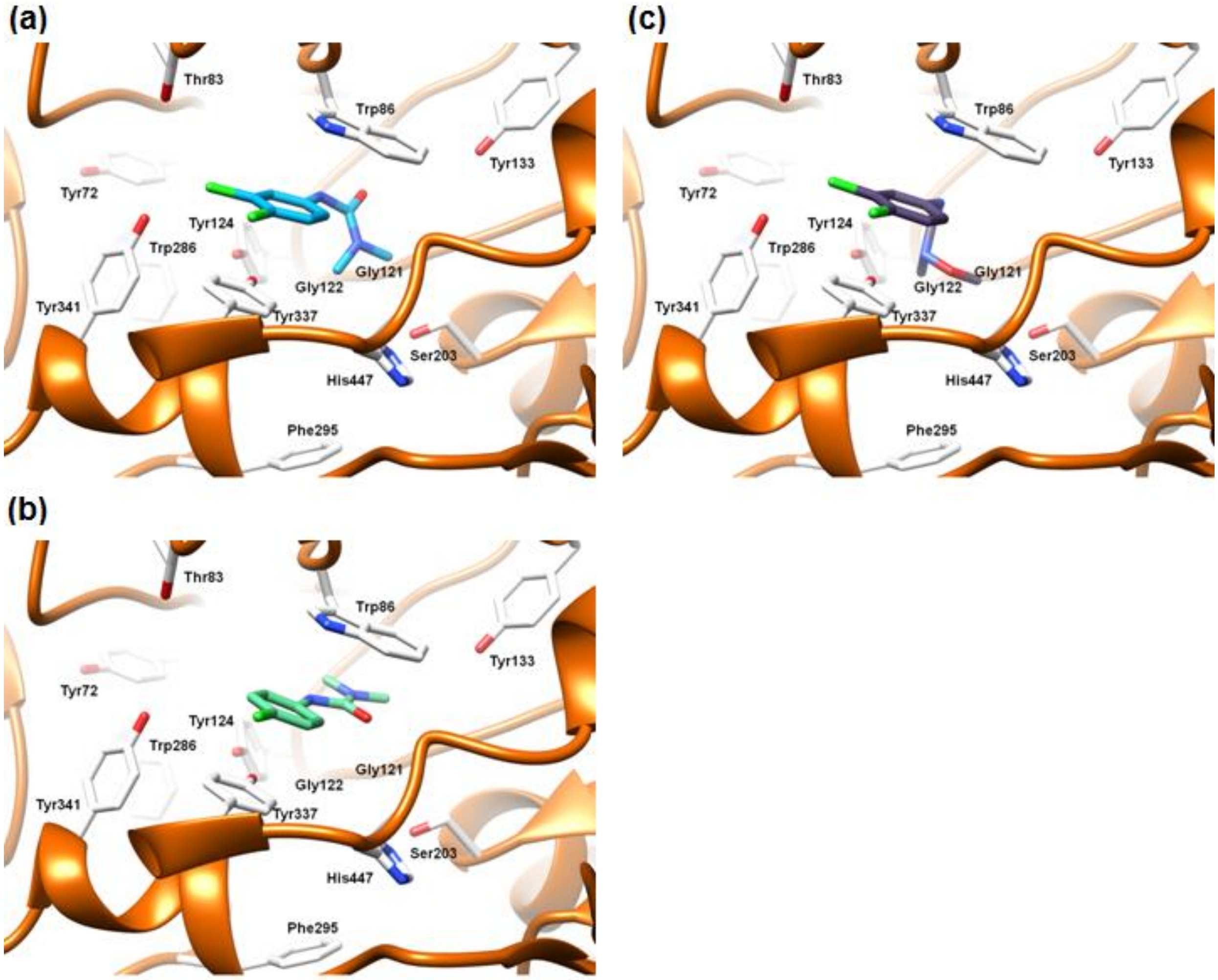

Figure S6. The The SB alignment of diuron (**a**), monuron (**b**), and linuron (**c**) into the *h*AChE active site. The The enzyme ribbons are presented in blue, active site amino acids are depicted in white. For the clarity purpose, hydrogen atoms are omitted from presentation.

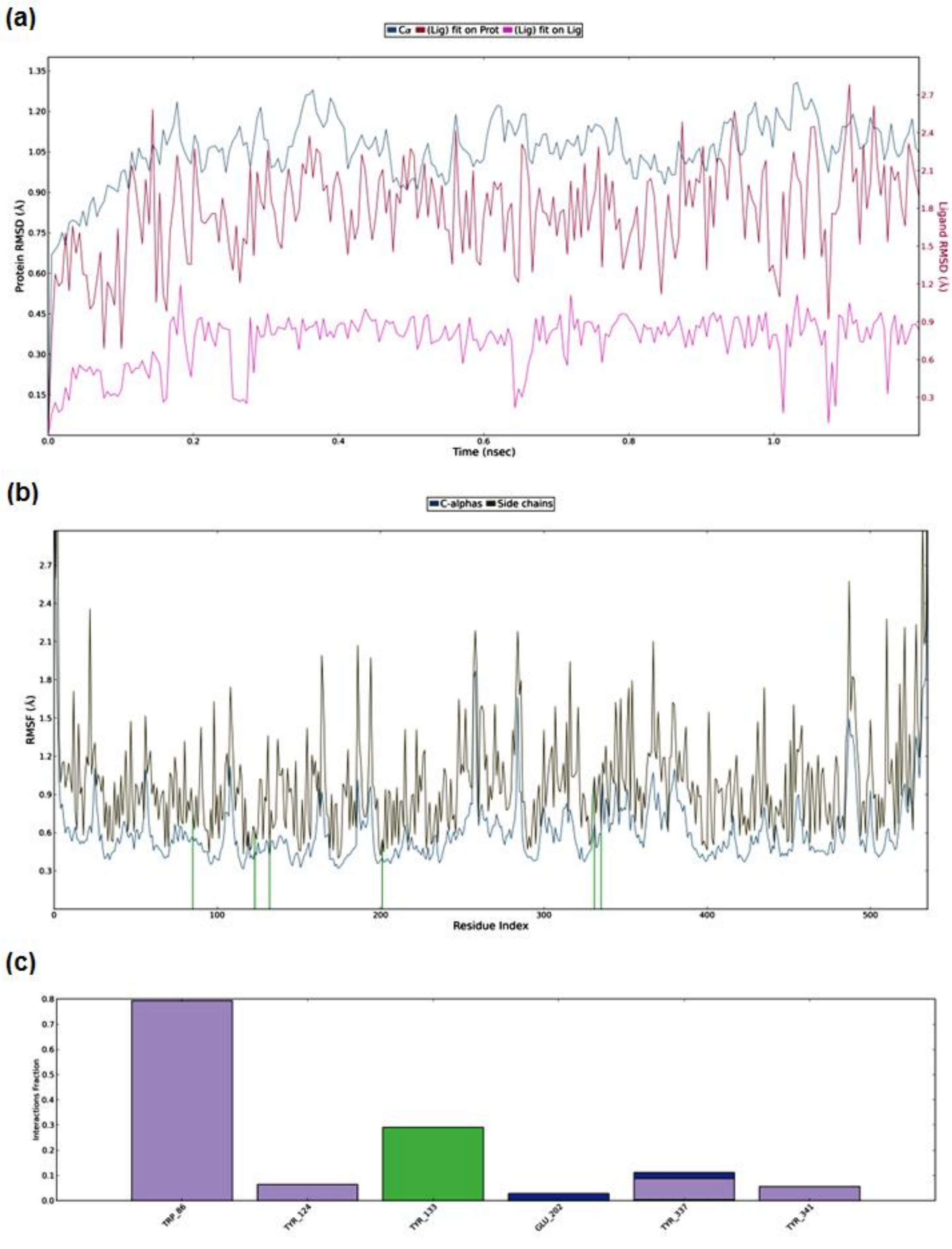

Figure S7. RMSDs (**a**), RMSFs (**b**), and Interaction diagrams (**c**) of *m*AChE in complex with ACh. The green bars present hydrogen bonds, the purple bars show hydrophobic interactions, the pink bars show ionic interaction, the blue bars present water bridges.

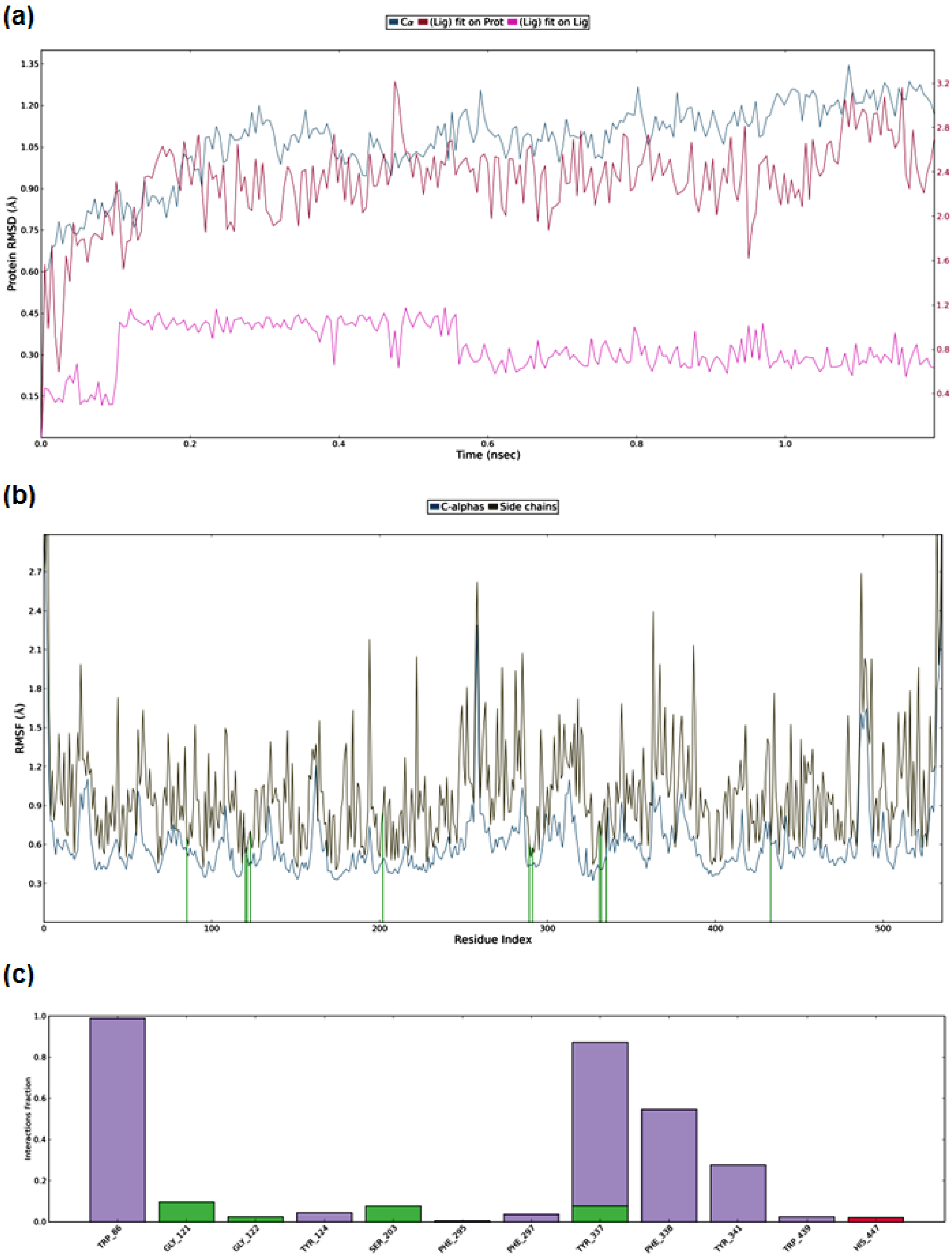

Figure S8. RMSDs (**a**), RMSFs (**b**), and Interaction diagrams (**c**) of *h*AChE in complex with ACh. The green bars present hydrogen bonds, the purple bars show hydrophobic interactions, the pink bars show ionic interaction, the blue bars present water bridges.

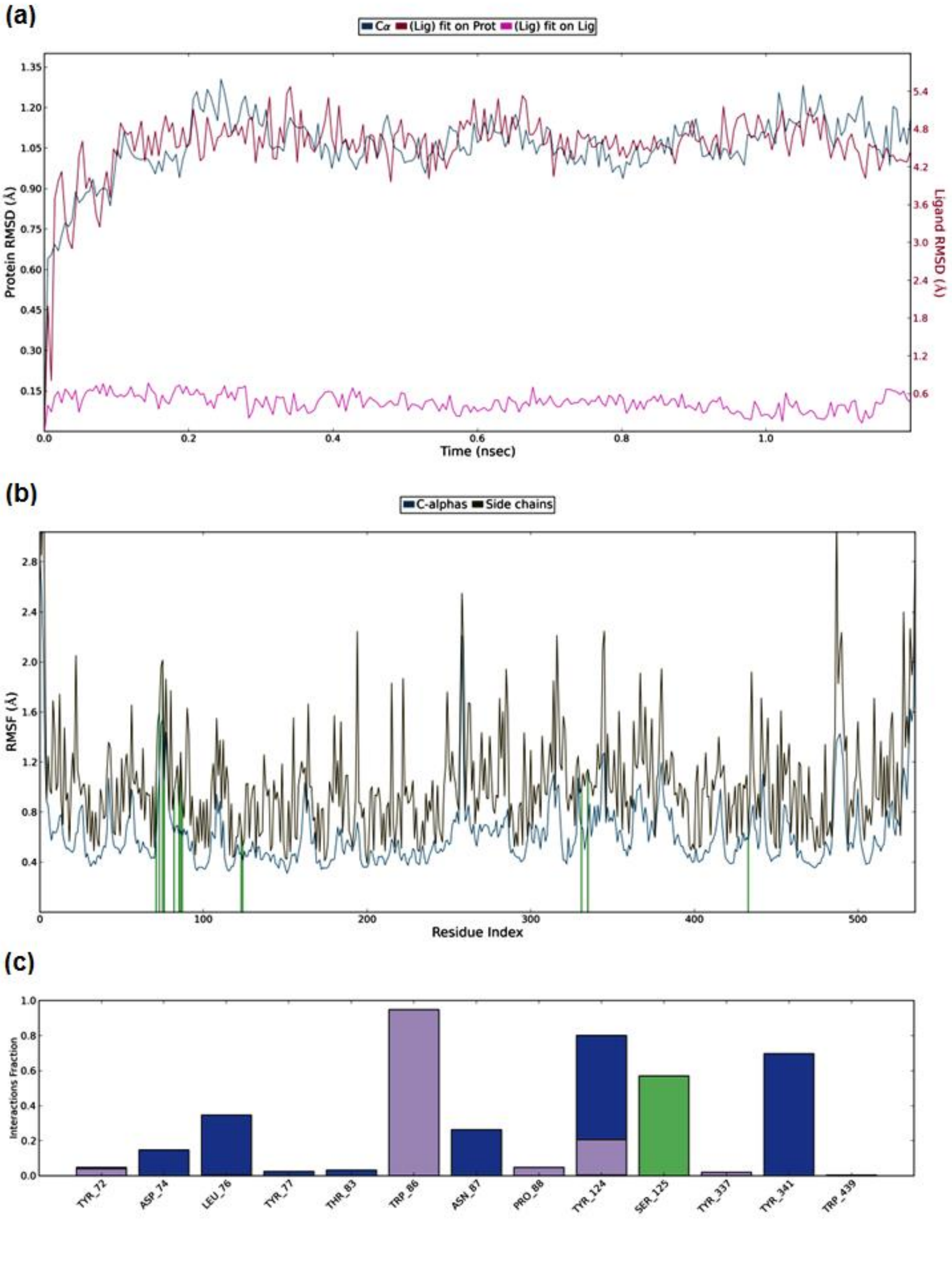

Figure S9. RMSDs (**a**), RMSFs (**b**), and Interaction diagrams (**c**) of *m*AChE in complex with atrazine. The green bars present hydrogen bonds, the purple bars show hydrophobic interactions, the pink bars show ionic interaction, the blue bars present water bridges.

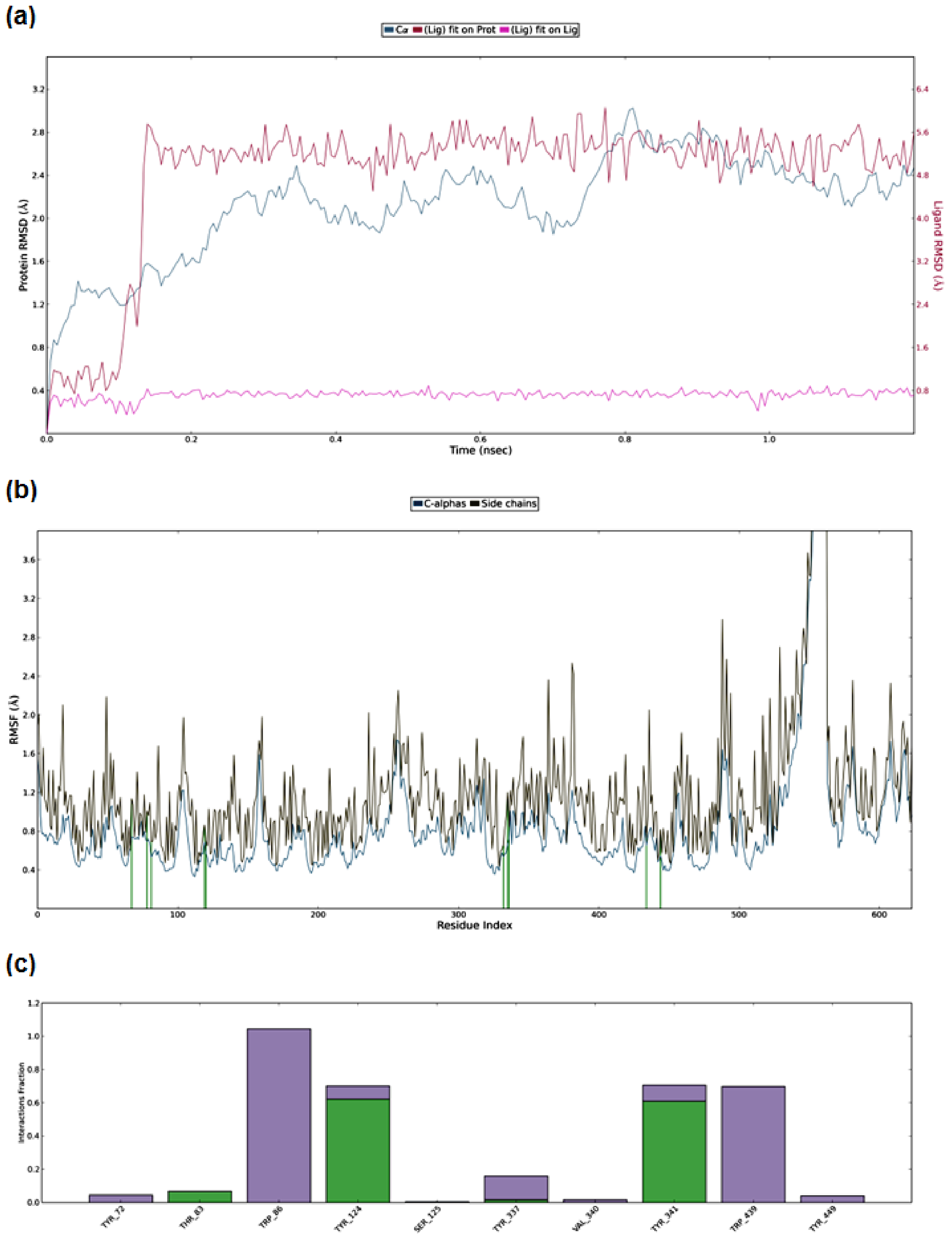

Figure S10. RMSDs (**a**), RMSFs (**b**), and Interaction diagrams (**c**) of *h*AChE in complex with atrazine. The green bars present hydrogen bonds, the purple bars show hydrophobic interactions, the pink bars show ionic interaction, the blue bars present water bridges.

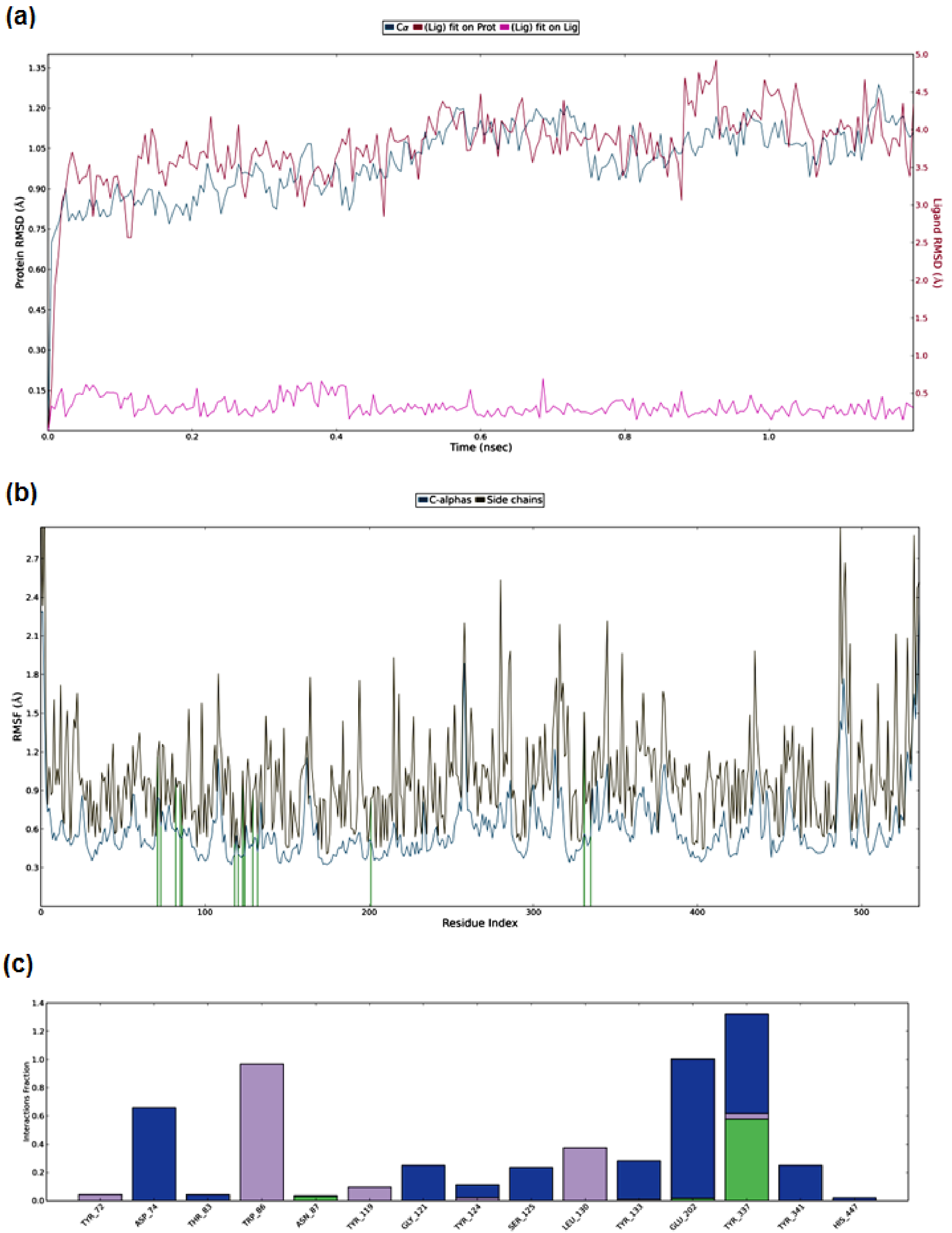

Figure S11. RMSDs (**a**), RMSFs (**b**), and Interaction diagrams (**c**) of *m*AChE in complex with propazine. The green bars present hydrogen bonds, the purple bars show hydrophobic interactions, the pink bars show ionic interaction, the blue bars present water bridges.

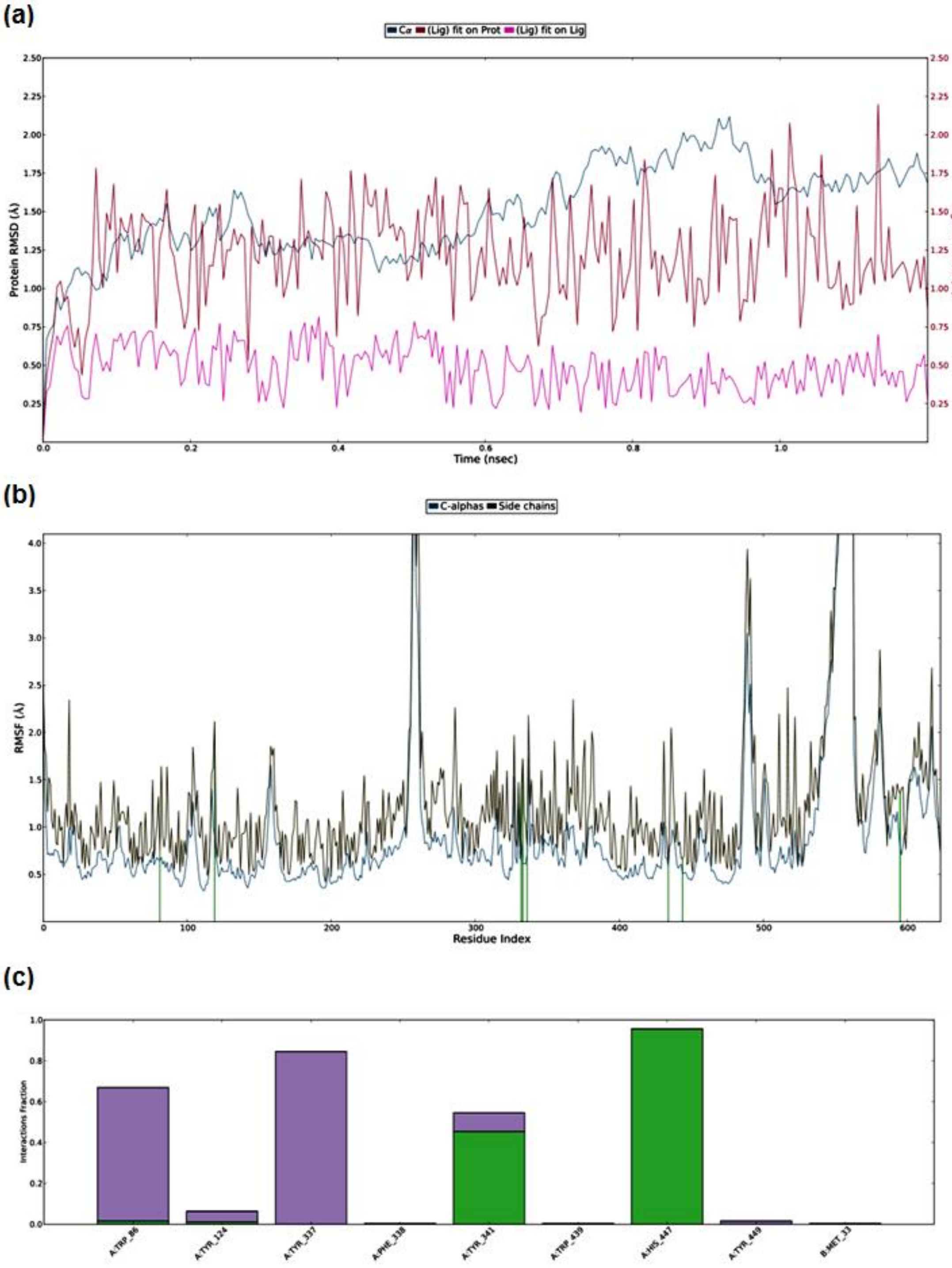

Figure S12. RMSDs (**a**), RMSFs (**b**), and Interaction diagrams (**c**) of *h*AChE in complex with propazine. The green bars present hydrogen bonds, the purple bars show hydrophobic interactions, the pink bars show ionic interaction, the blue bars present water bridges.

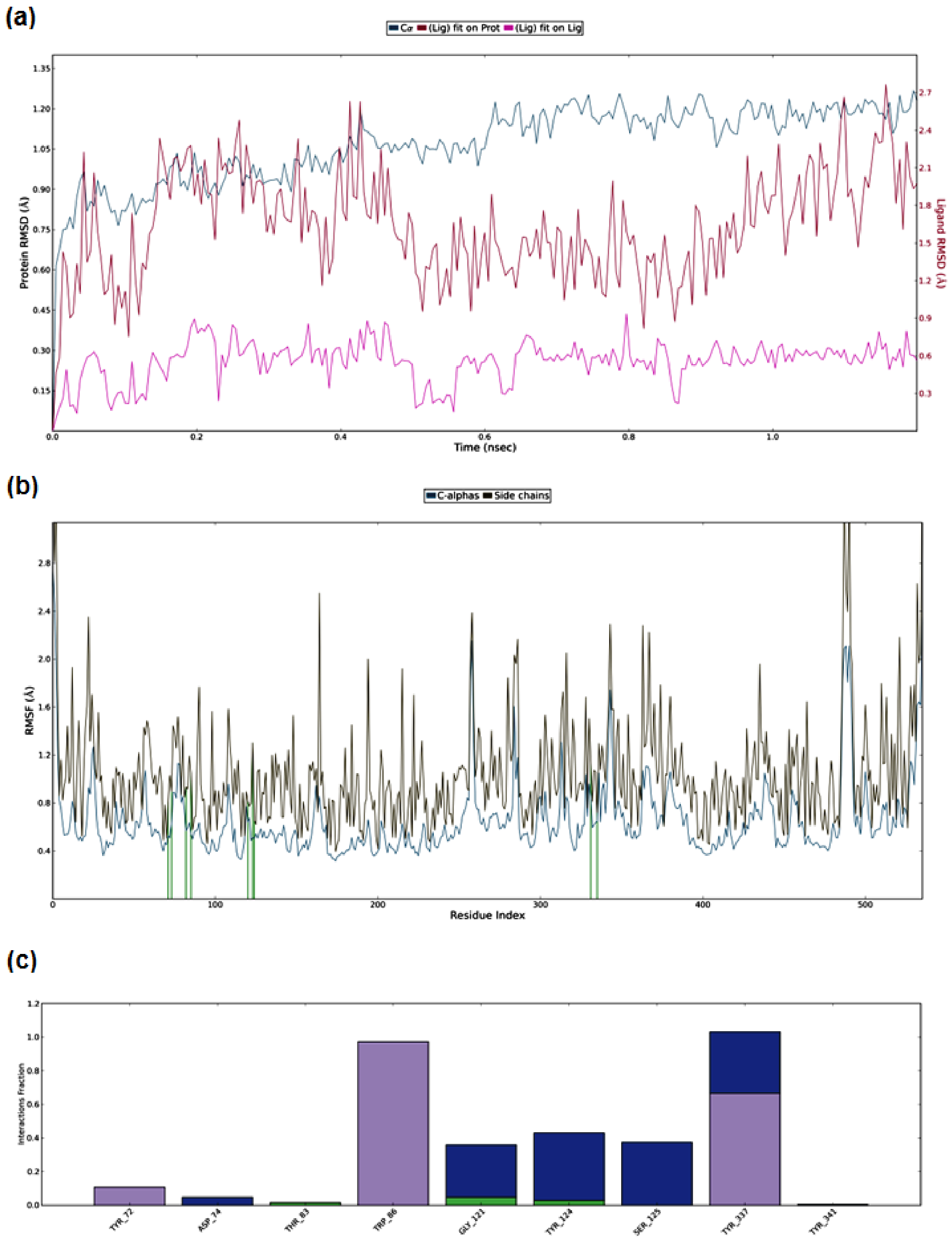

Figure S13. RMSDs (**a**), RMSFs (**b**), and Interaction diagrams (**c**) of *m*AChE in complex with simazine. The green bars present hydrogen bonds, the purple bars show hydrophobic interactions, the pink bars show ionic interaction, the blue bars present water bridges.

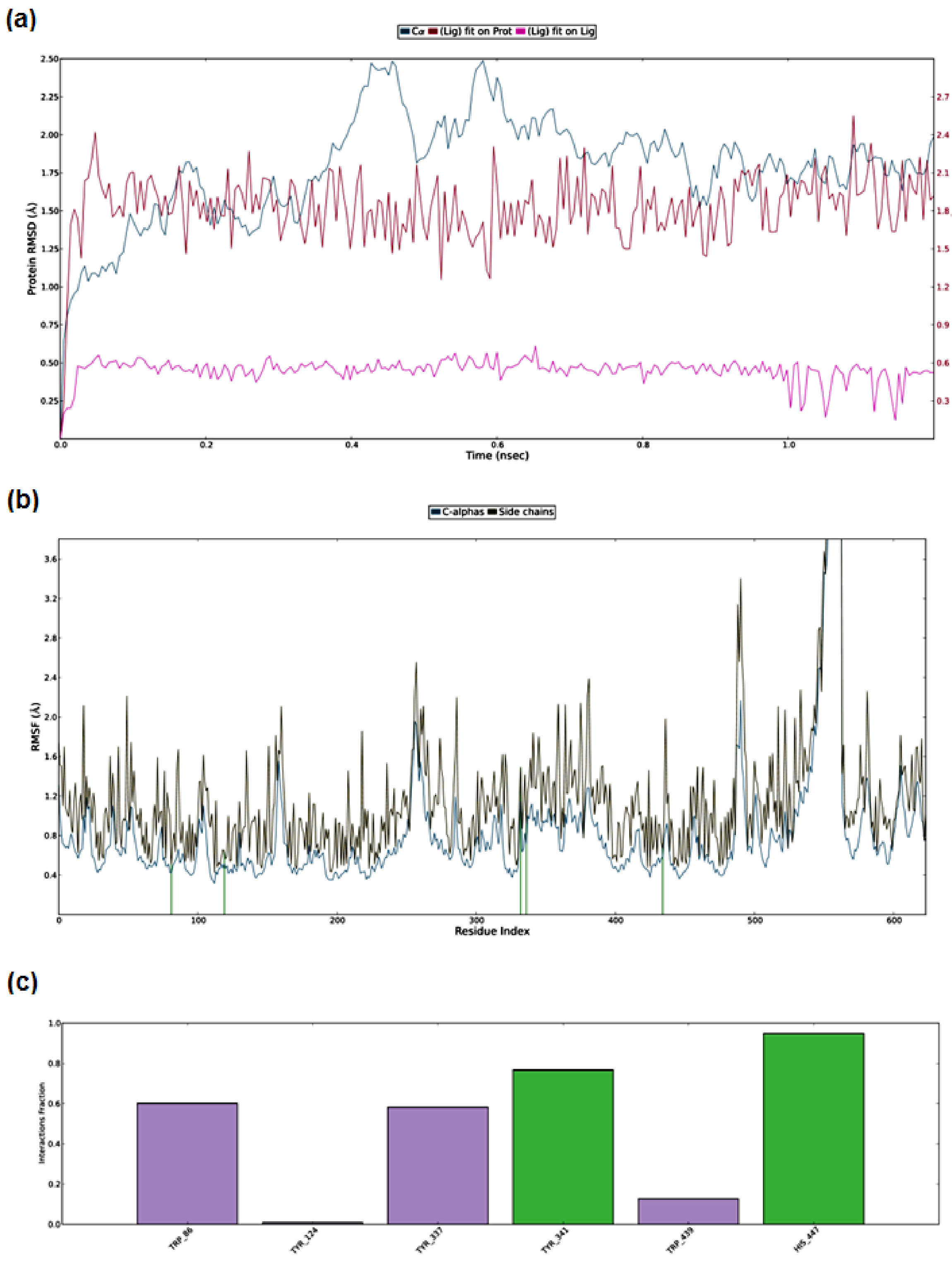

Figure S14. RMSDs (**a**), RMSFs (**b**), and Interaction diagrams (**c**) of *h*AChE in complex with simazine. The green bars present hydrogen bonds, the purple bars show hydrophobic interactions, the pink bars show ionic interaction, the blue bars present water bridges.

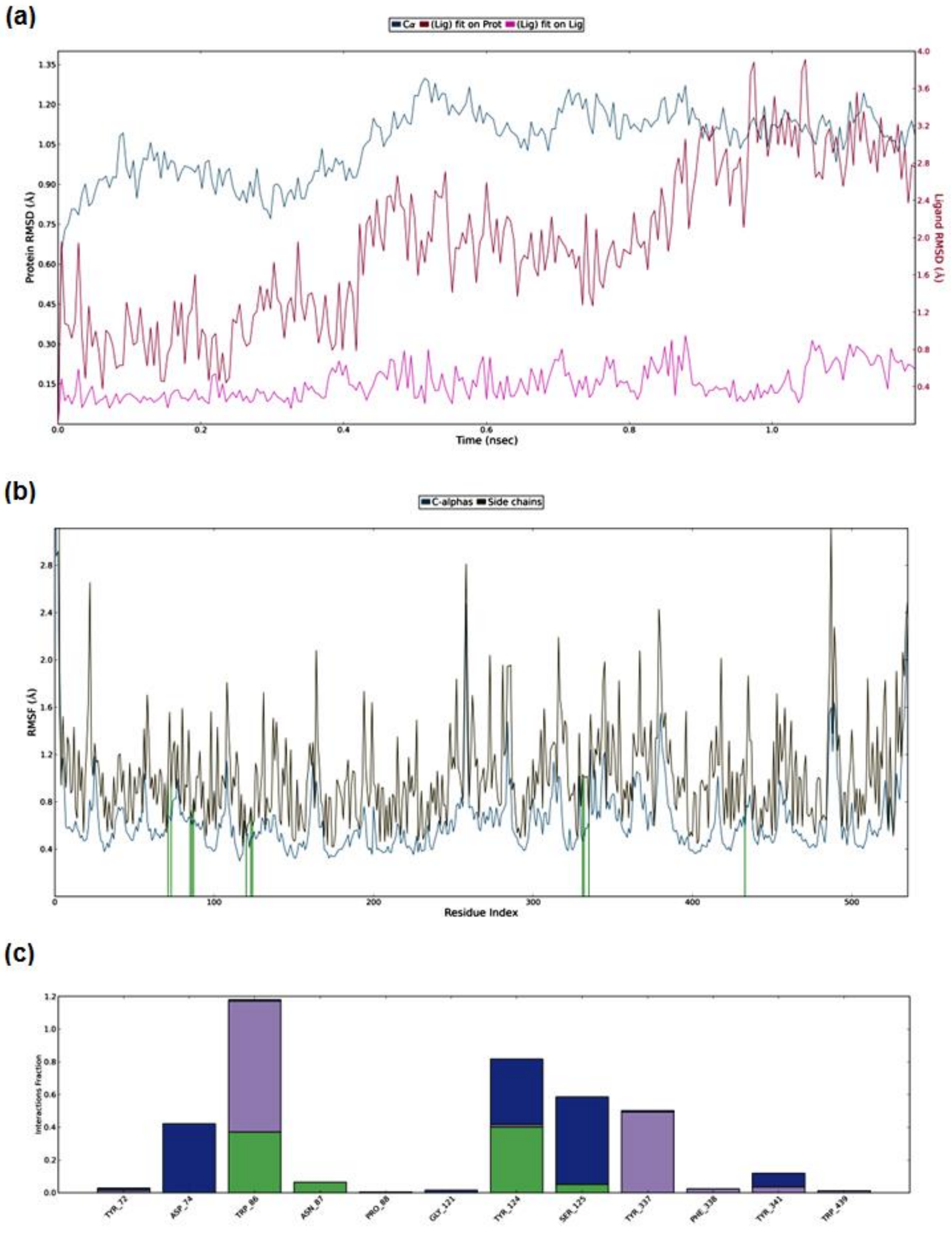

Figure S15. RMSDs (**a**), RMSFs (**b**), and Interaction diagrams (**c**) of *m*AChE in complex with carbofuran. The green bars present hydrogen bonds, the purple bars show hydrophobic interactions, the pink bars show ionic interaction, the blue bars present water bridges.

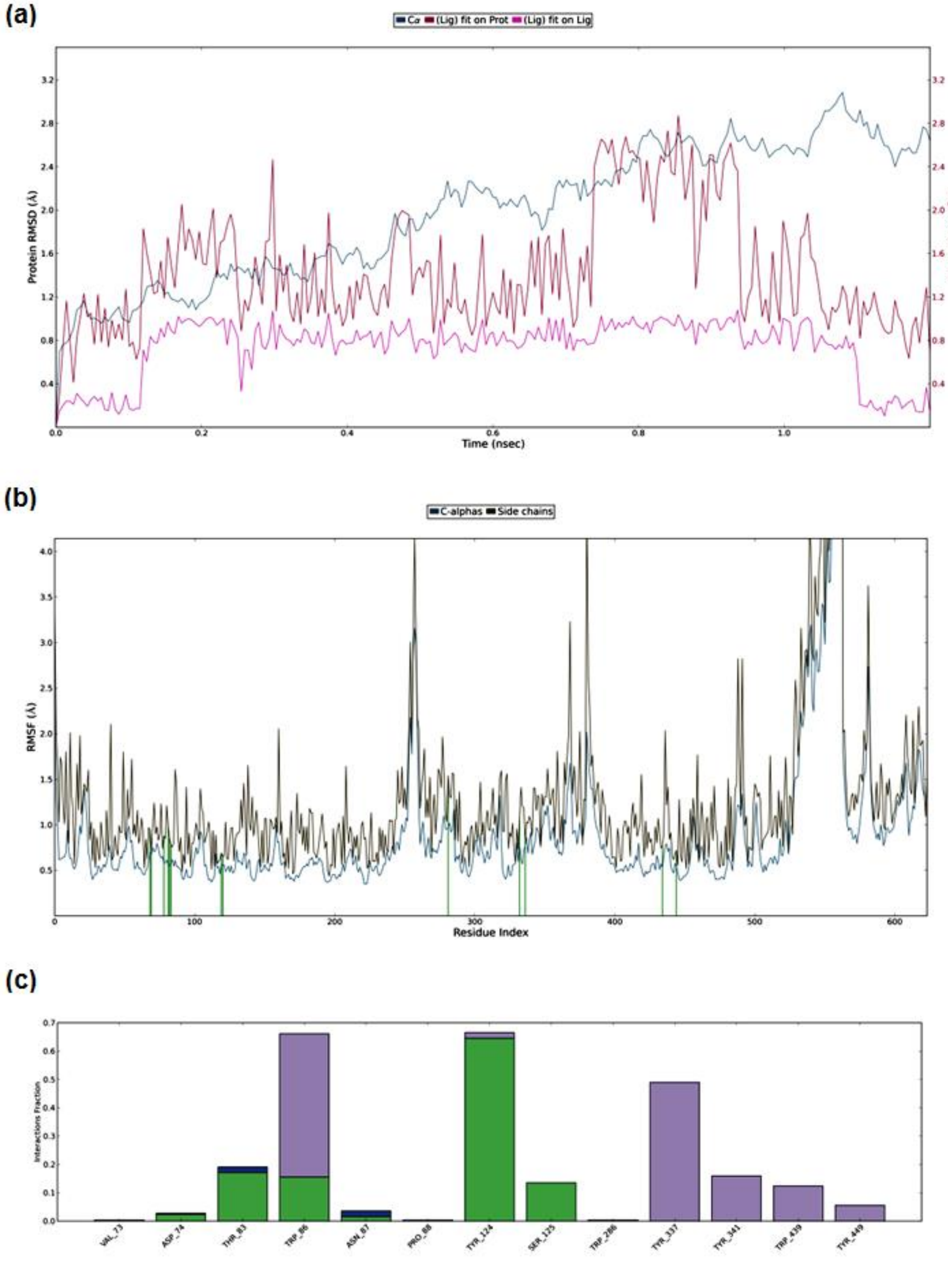

Figure S16. RMSDs (**a**), RMSFs (**b**), and Interaction diagrams (**c**) of *h*AChE in complex with carbofuran. The green bars present hydrogen bonds, the purple bars show hydrophobic interactions, the pink bars show ionic interaction, the blue bars present water bridges.

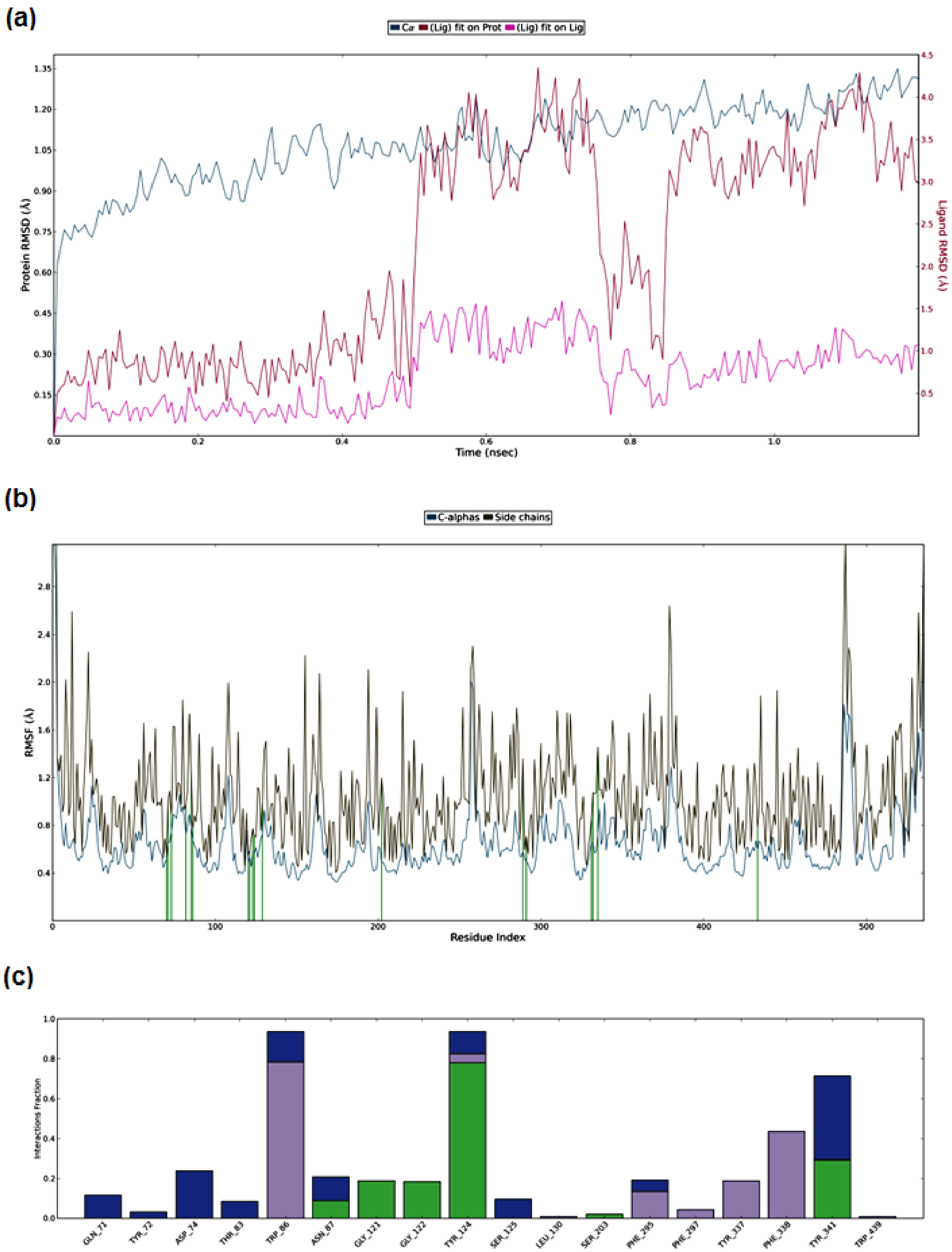

Figure S17. RMSDs (**a**), RMSFs (**b**), and Interaction diagrams (**c**) of *m*AChE in complex with monocrotophos. The green bars present hydrogen bonds, the purple bars show hydrophobic interactions, the pink bars show ionic interaction, the blue bars present water bridges.

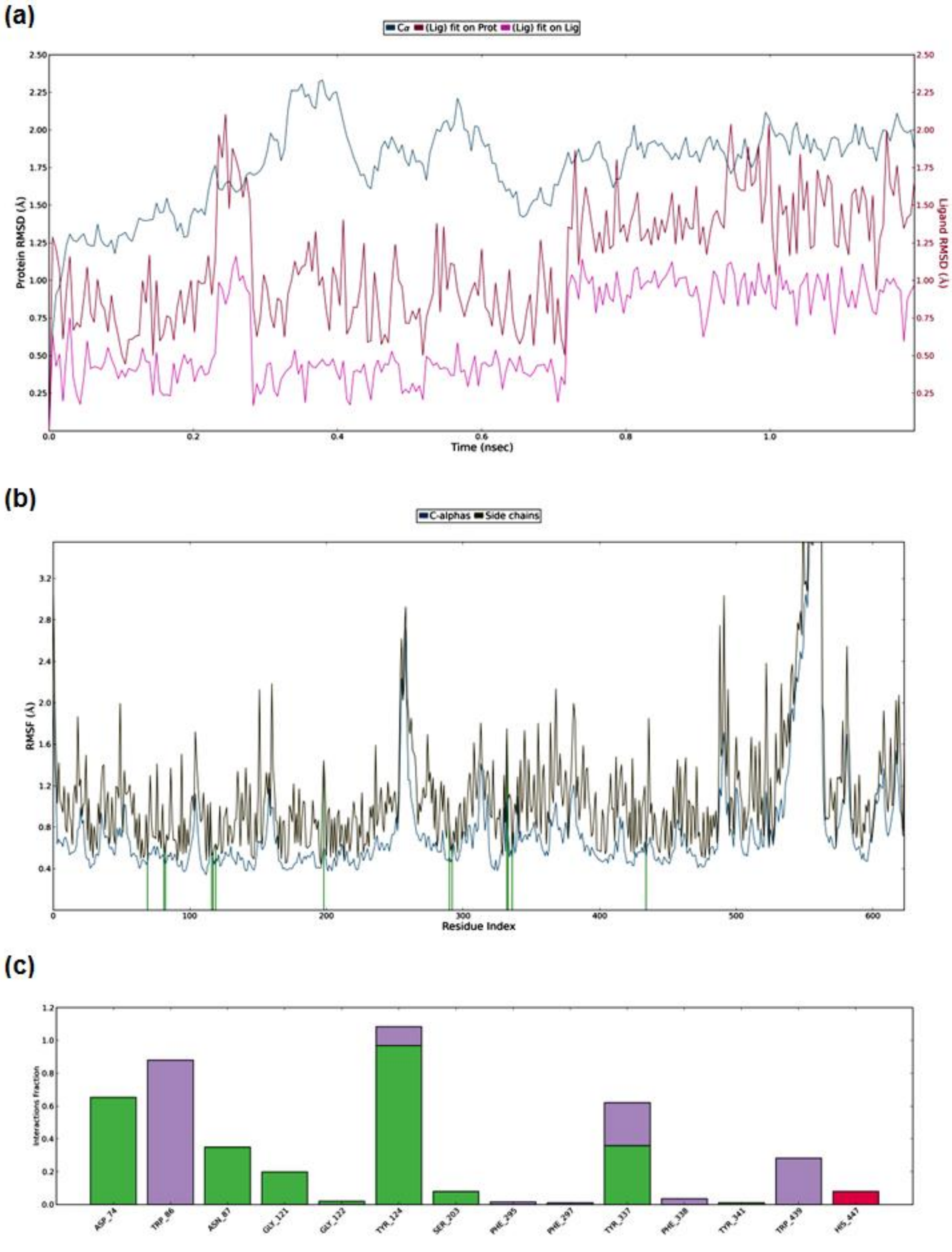

Figure S18. RMSDs (**a**), RMSFs (**b**), and Interaction diagrams (**c**) of *h*AChE in complex with monocrotophos. The green bars present hydrogen bonds, the purple bars show hydrophobic interactions, the pink bars show ionic interaction, the blue bars present water bridges.

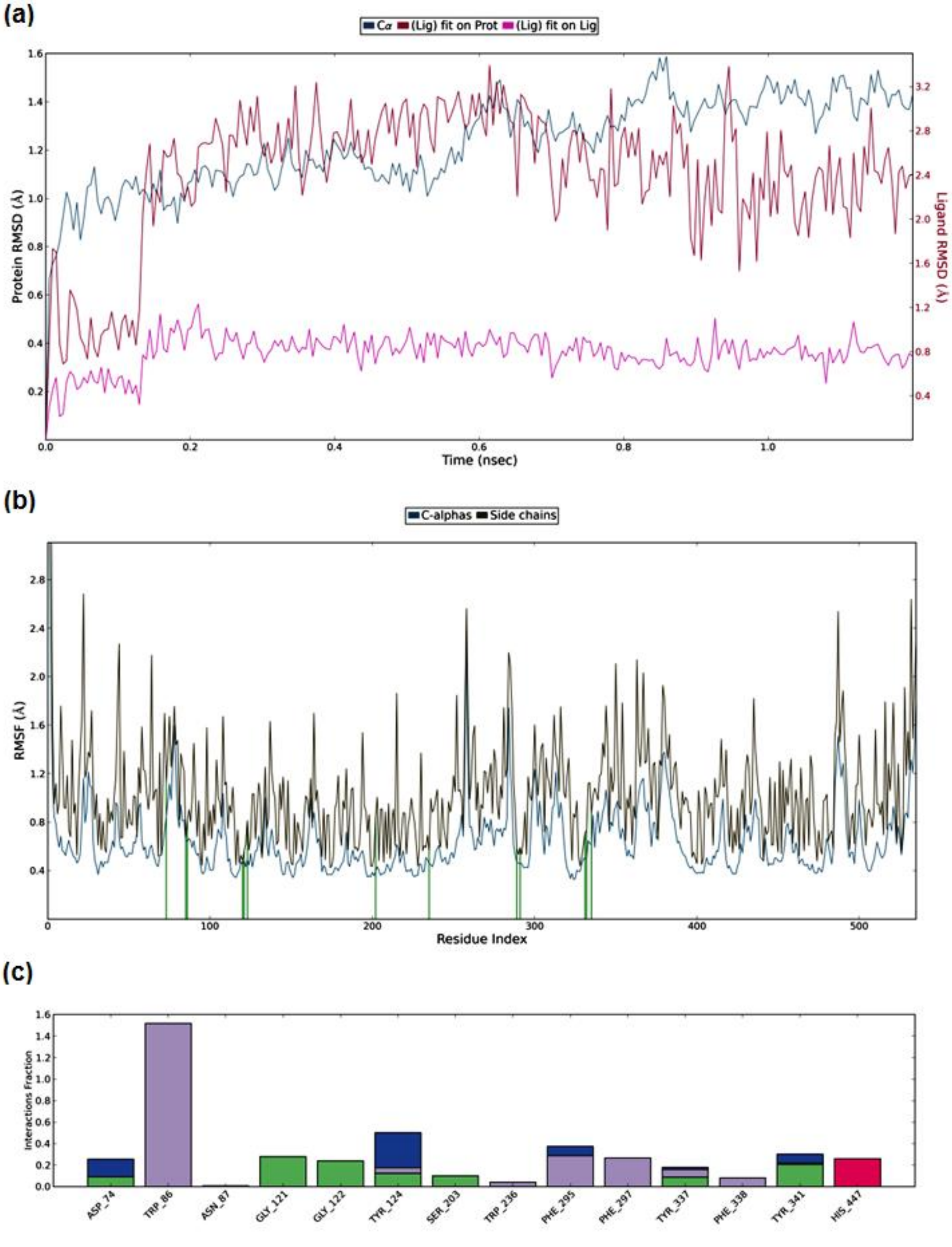

Figure S19. RMSDs (**a**), RMSFs (**b**), and Interaction diagrams (**c**) of *m*AChE in complex with dimethoate. The green bars present hydrogen bonds, the purple bars show hydrophobic interactions, the pink bars show ionic interaction, the blue bars present water bridges.

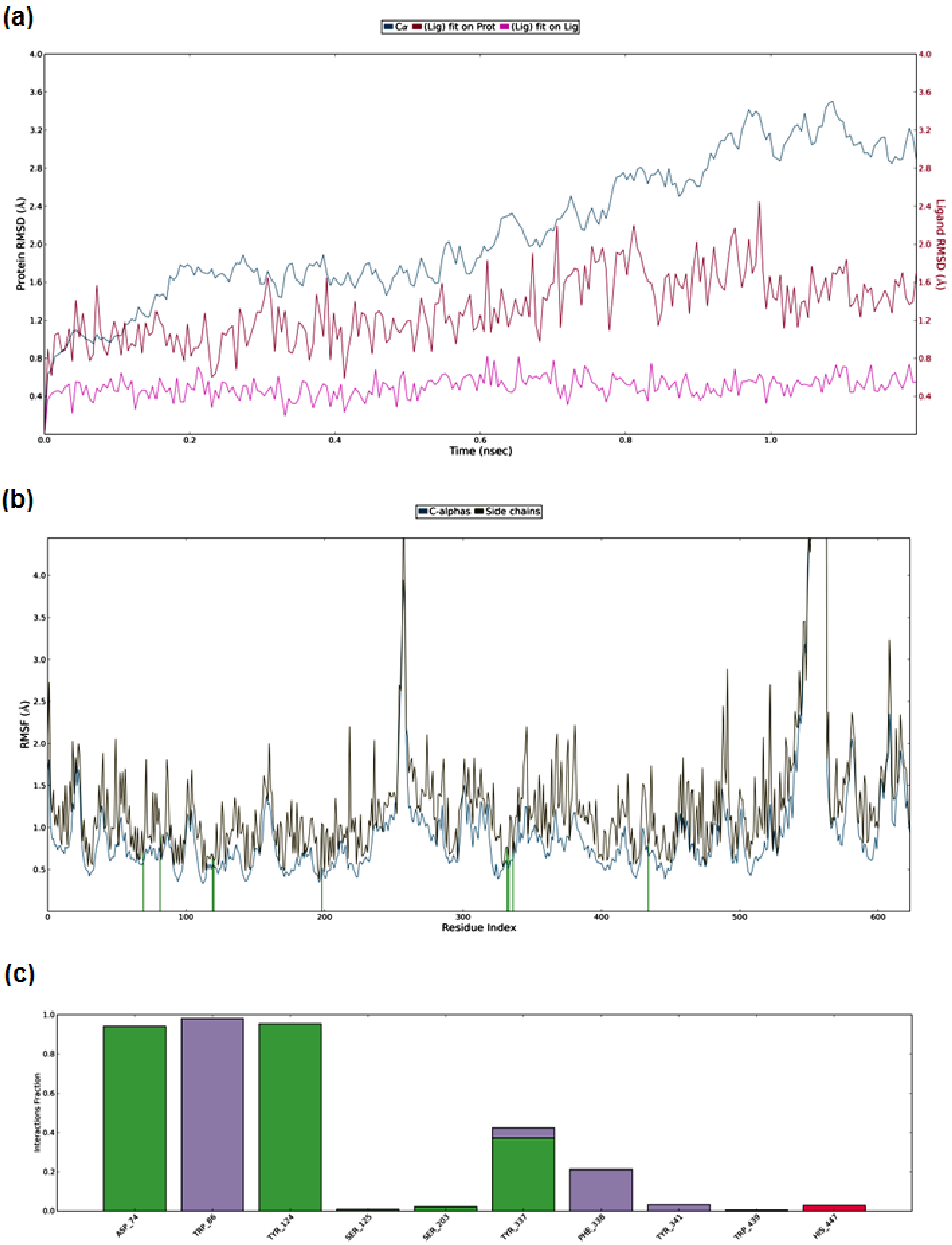

Figure S20. RMSDs (**a**), RMSFs (**b**), and Interaction diagrams (**c**) of *h*AChE in complex with dimethoate. The green bars present hydrogen bonds, the purple bars show hydrophobic interactions, the pink bars show ionic interaction, the blue bars present water bridges.

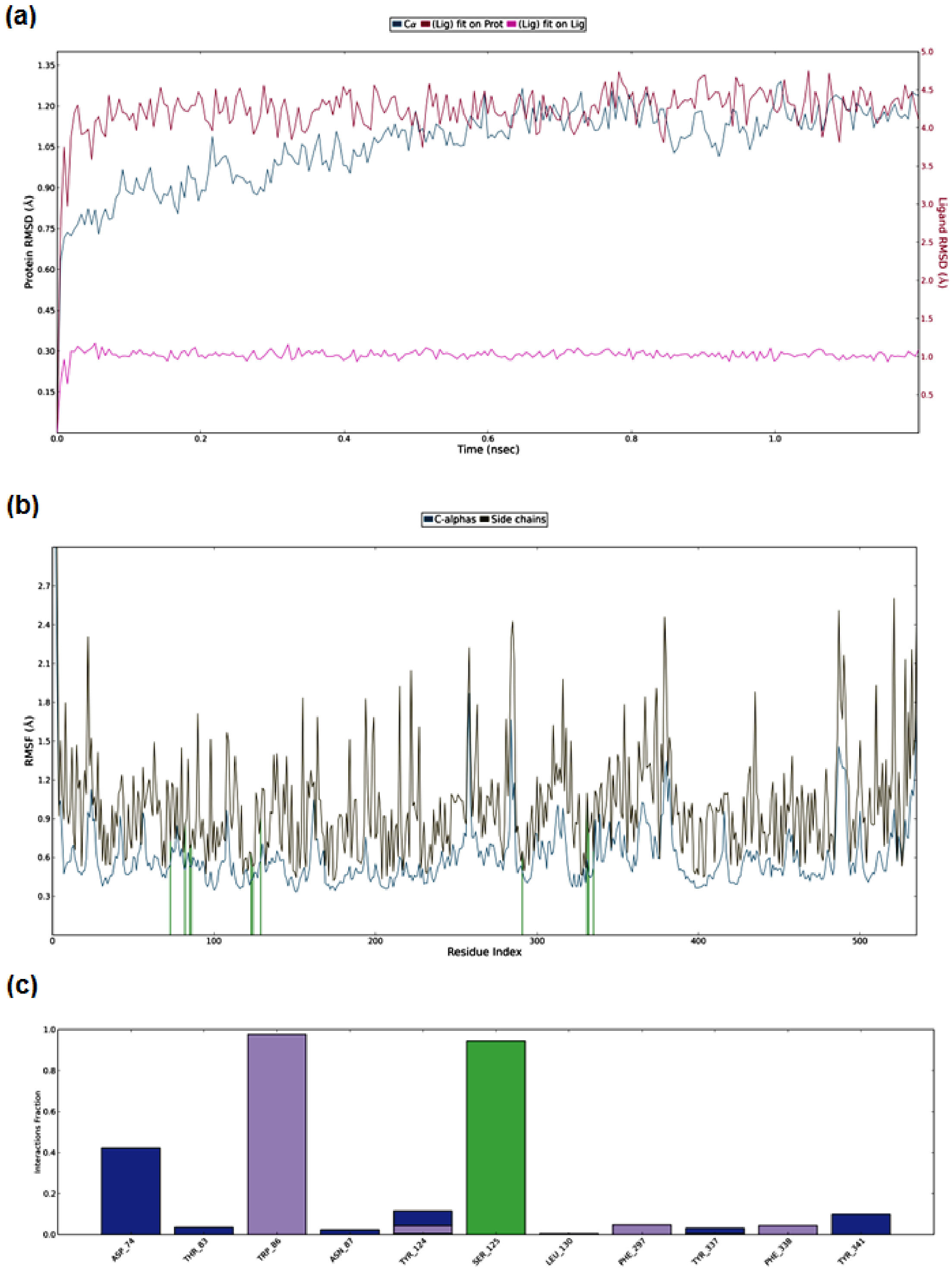

Figure S21. RMSDs (**a**), RMSFs (**b**), and Interaction diagrams (**c**) of *m*AChE in complex with carbaryl. The green bars present hydrogen bonds, the purple bars show hydrophobic interactions, the pink bars show ionic interaction, the blue bars present water bridges.

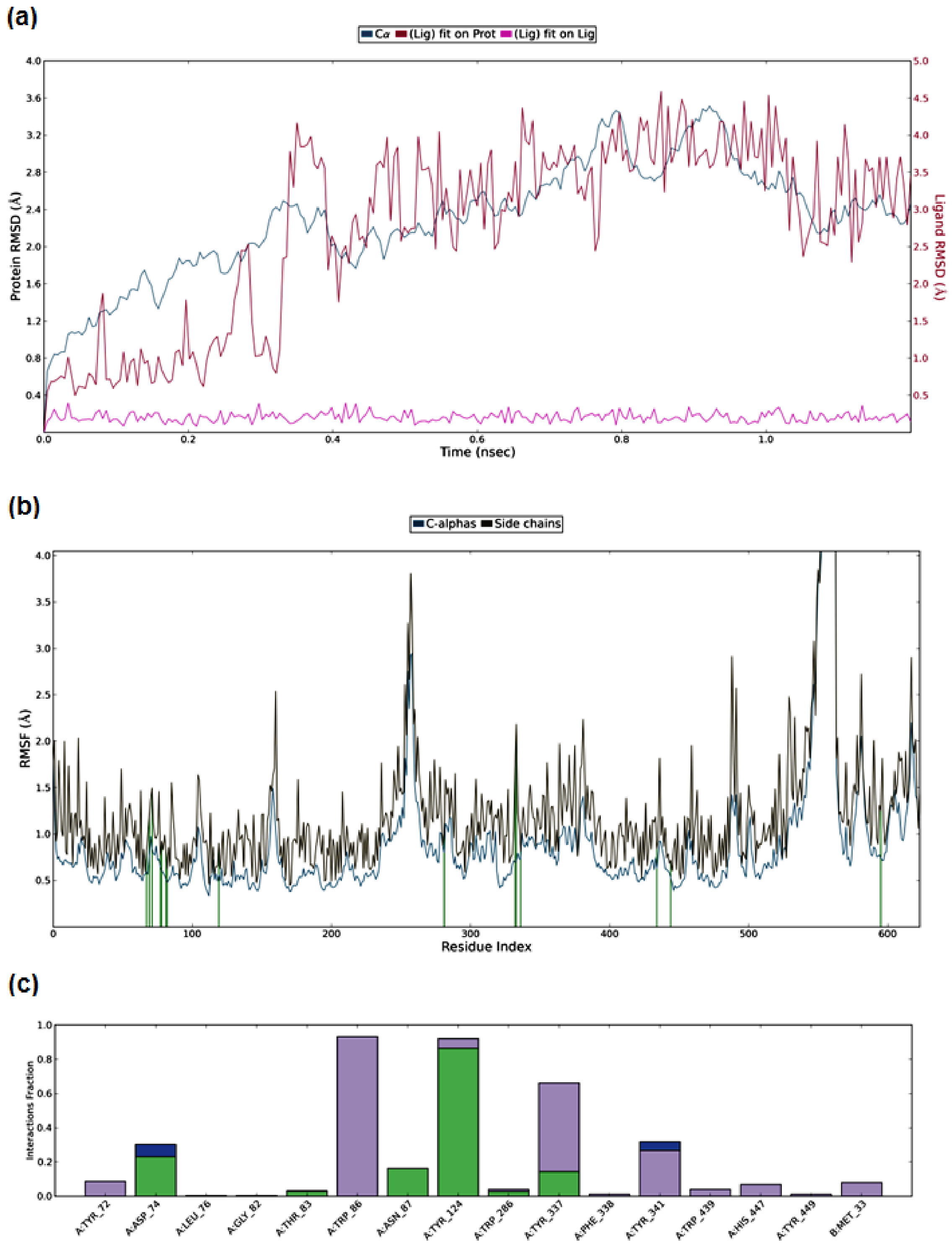

Figure S22. RMSDs (**a**), RMSFs (**b**), and Interaction diagrams (**c**) of *h*AChE in complex with carbaryl. The green bars present hydrogen bonds, the purple bars show hydrophobic interactions, the pink bars show ionic interaction, the blue bars present water bridges.

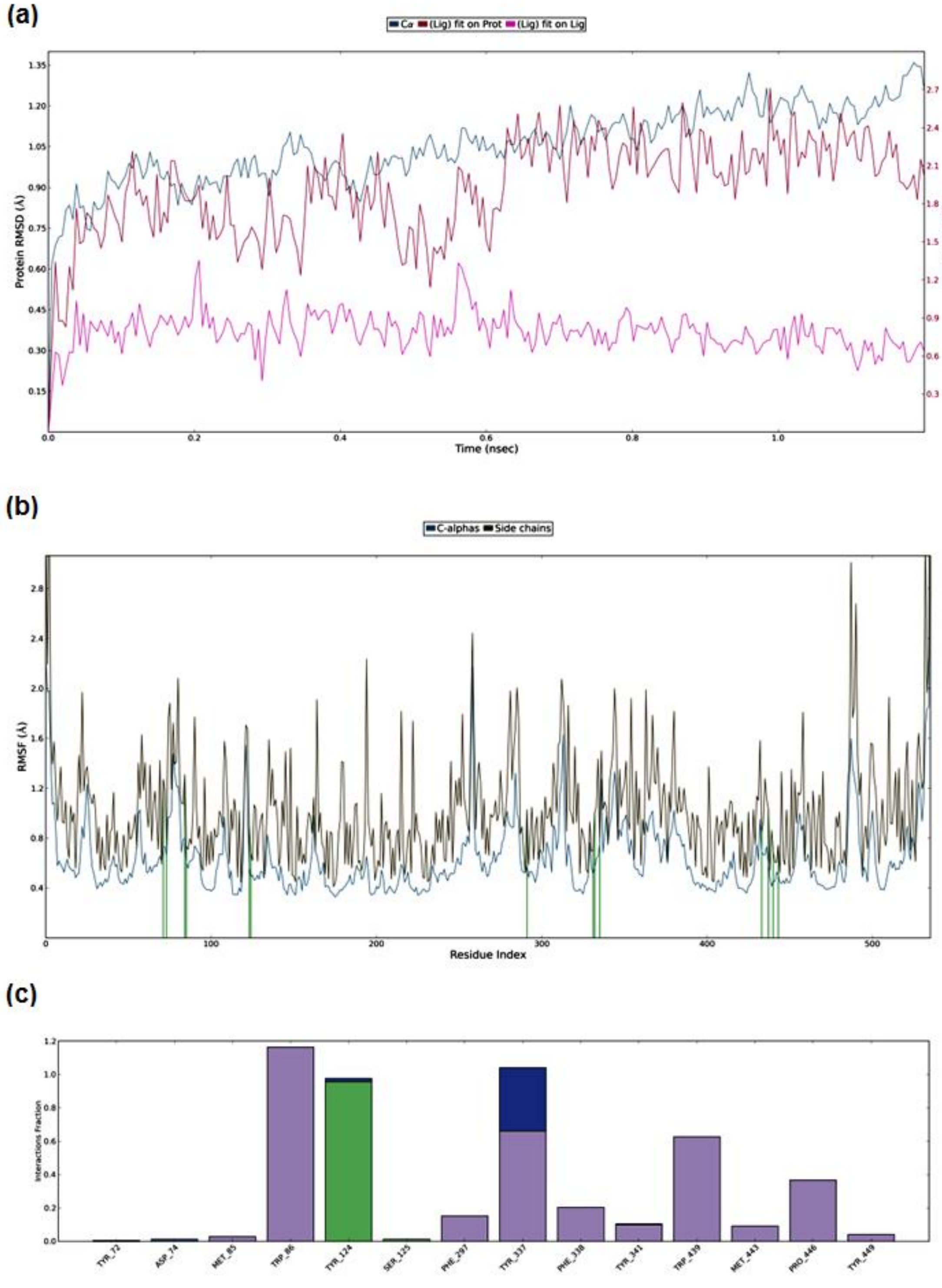

Figure S23. RMSDs (**a**), RMSFs (**b**), and Interaction diagrams (**c**) of *m*AChE in complex with tebufenozide. The green bars present hydrogen bonds, the purple bars show hydrophobic interactions, the pink bars show ionic interaction, the blue bars present water bridges.

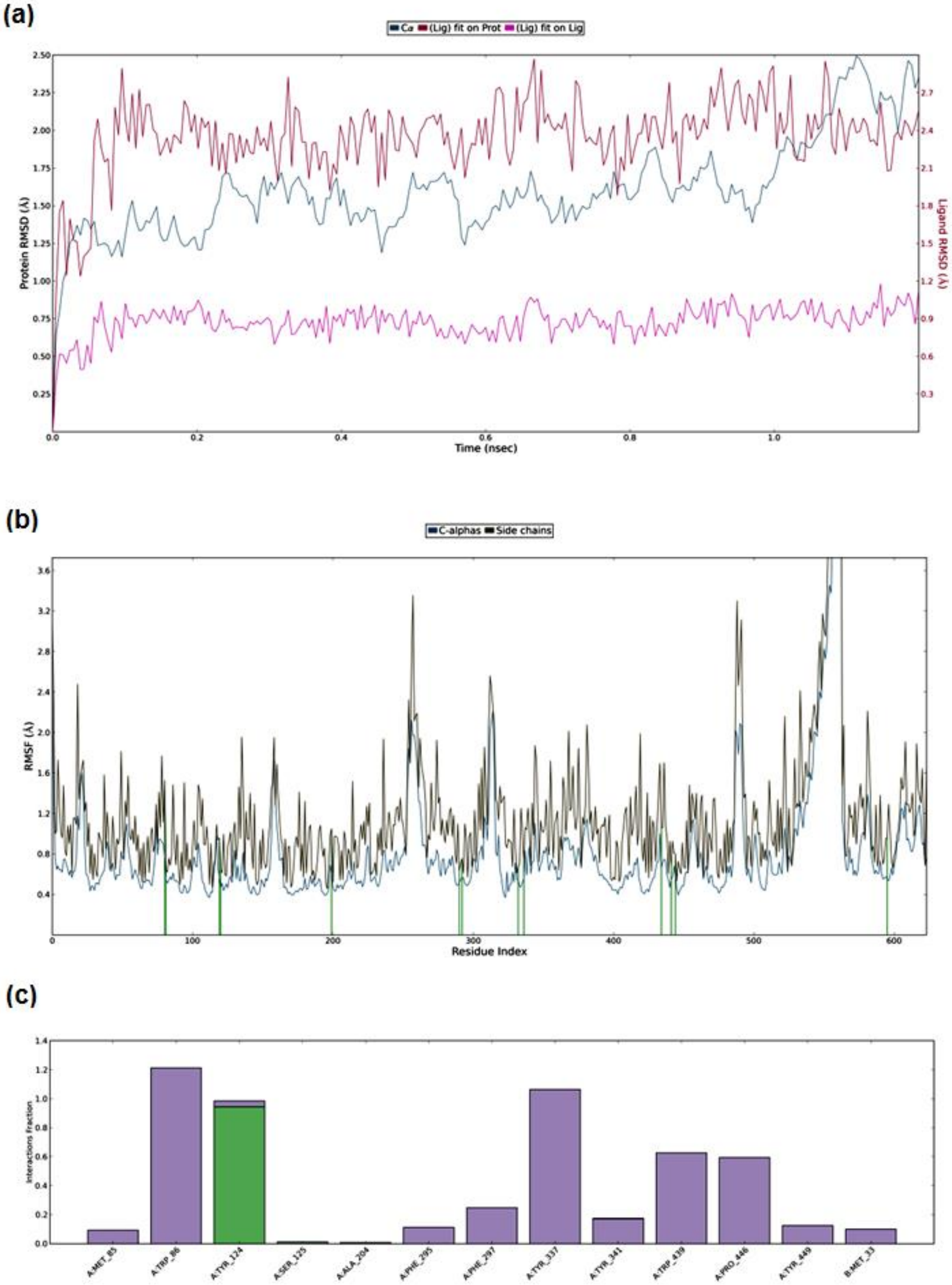

Figure S24. RMSDs (**a**), RMSFs (**b**), and Interaction diagrams (**c**) of *h*AChE in complex with tebufenozide. The green bars present hydrogen bonds, the purple bars show hydrophobic interactions, the pink bars show ionic interaction, the blue bars present water bridges.

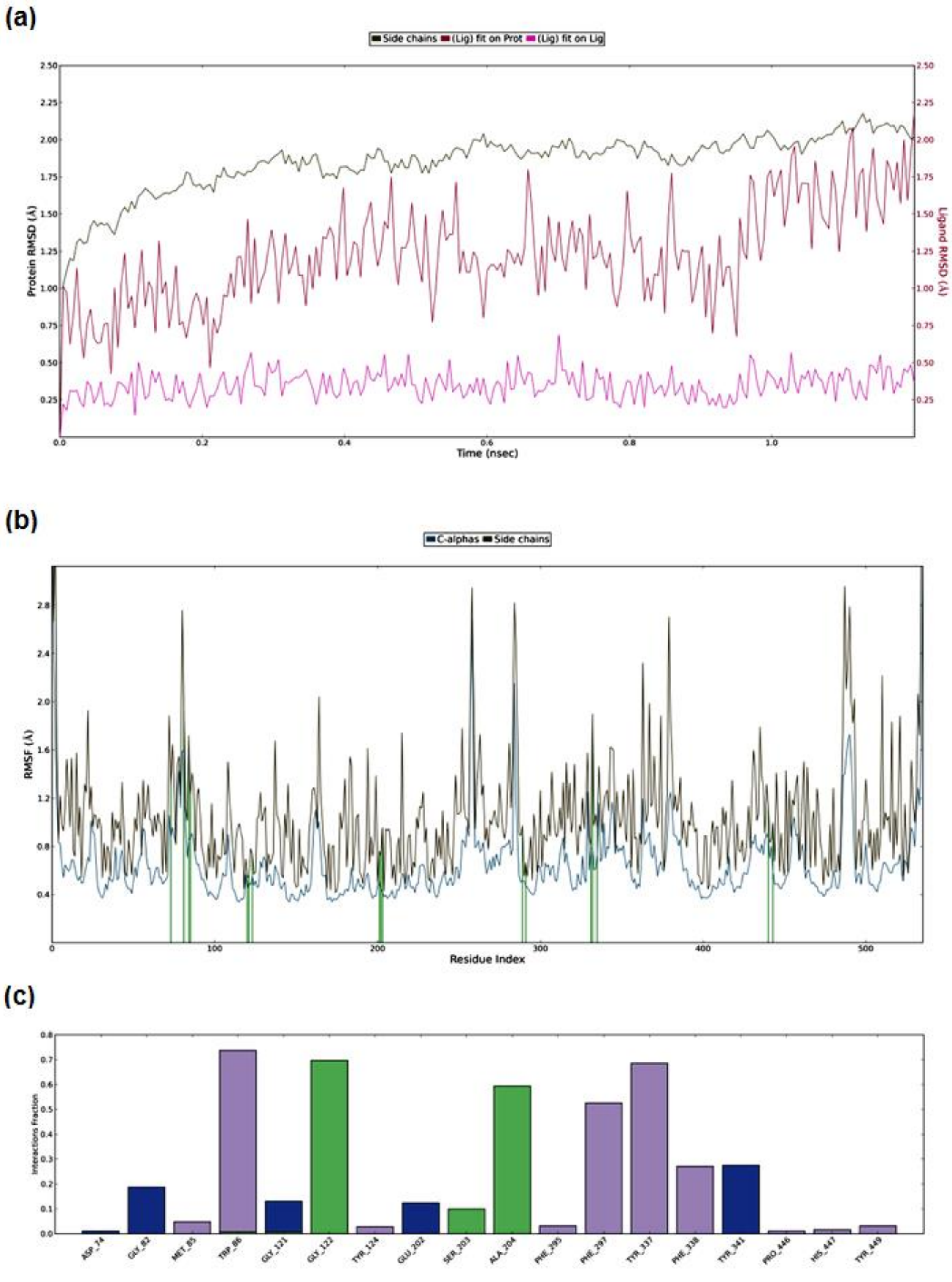

Figure S25. RMSDs (**a**), RMSFs (**b**), and Interaction diagrams (**c**) of *m*AChE in complex with imidacloprid. The green bars present hydrogen bonds, the purple bars show hydrophobic interactions, the pink bars show ionic interaction, the blue bars present water bridges.

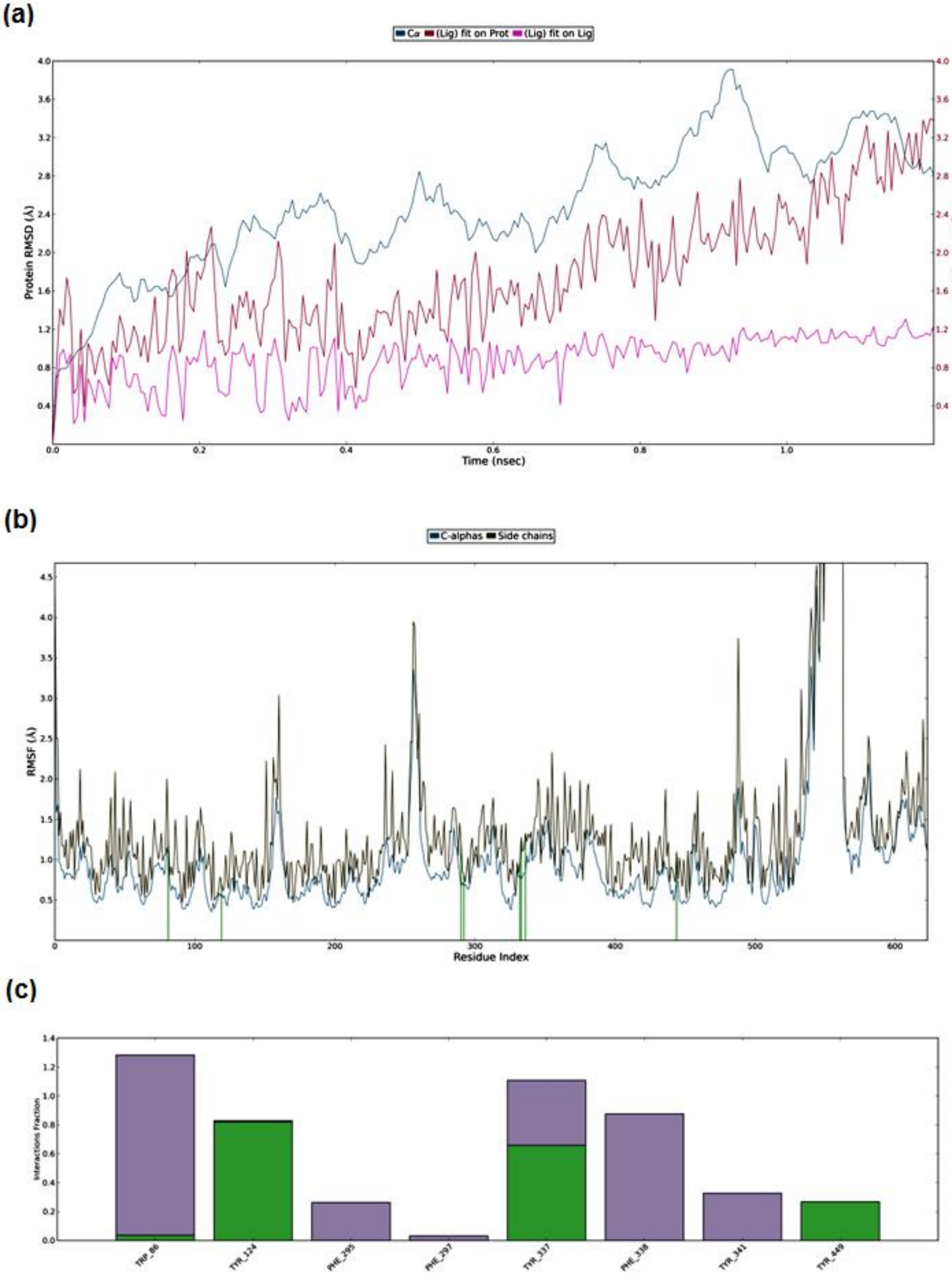

Figure S26. RMSDs (**a**), RMSFs (**b**), Interaction diagrams (**c**) of *h*AChE in complex with imidacloprid. The green bars present hydrogen bonds, the purple bars show hydrophobic interactions, the pink bars show ionic interaction, the blue bars present water bridges.

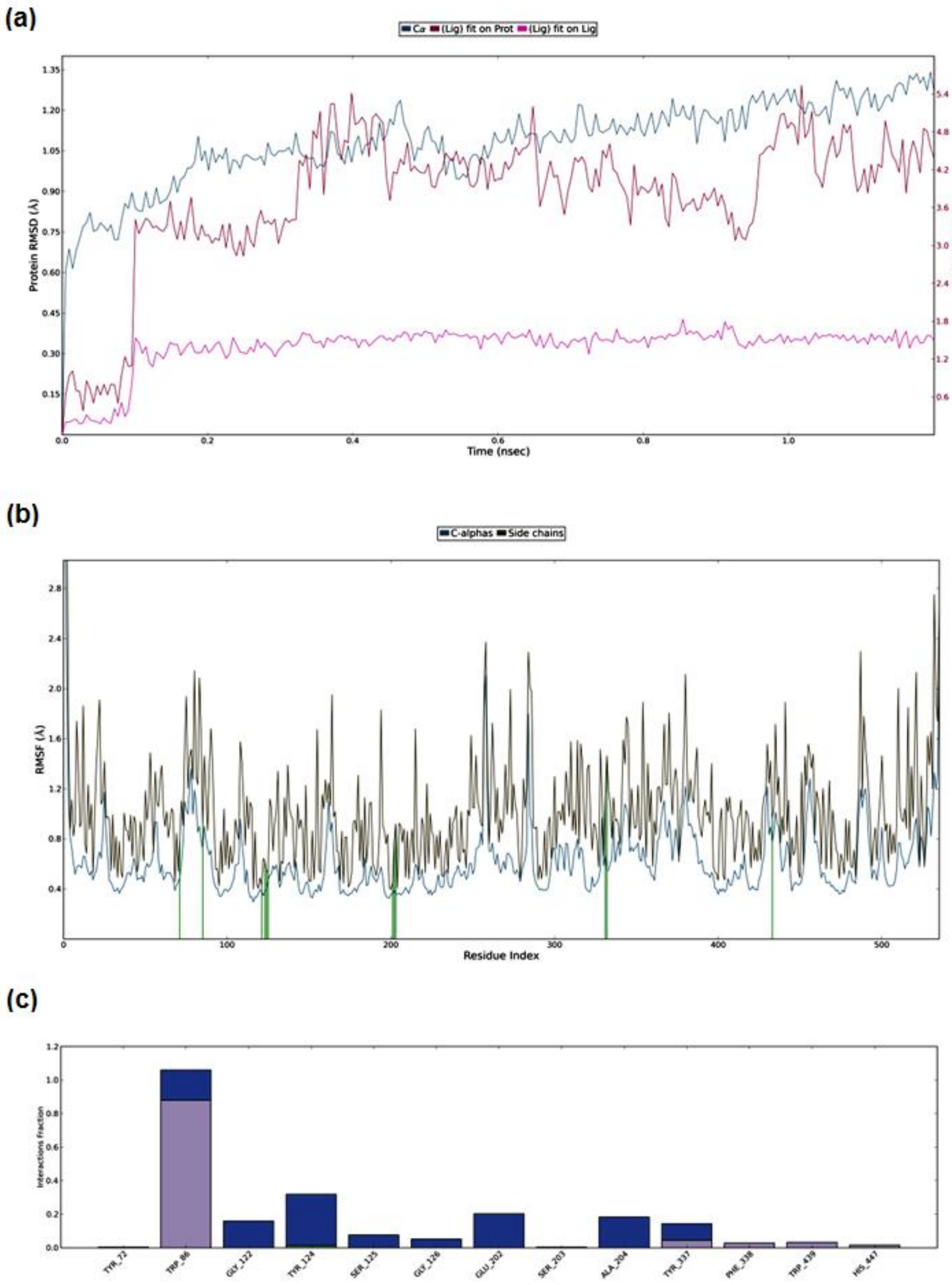

Figure S27. RMSDs (**a**), RMSFs (**b**), and Interaction diagrams (**c**) of *m*AChE in complex with acetamiprid. The green bars present hydrogen bonds, the purple bars show hydrophobic interactions, the pink bars show ionic interaction, the blue bars present water bridges.

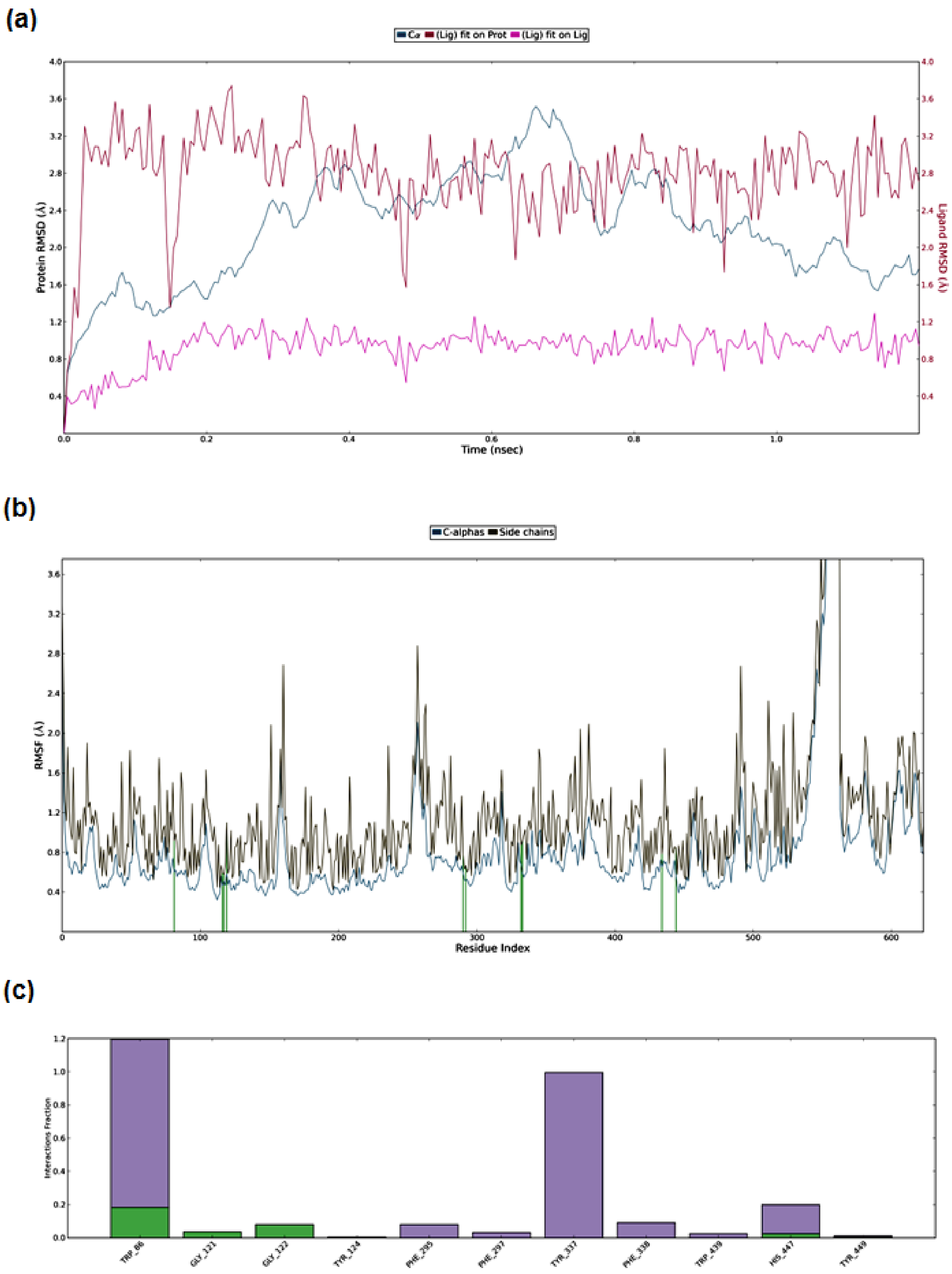

Figure S28. RMSDs (**a**), RMSFs (**b**), and Interaction diagrams (**c**) of *h*AChE in complex with acetamiprid. The green bars present hydrogen bonds, the purple bars show hydrophobic interactions, the pink bars show ionic interaction, the blue bars present water bridges.

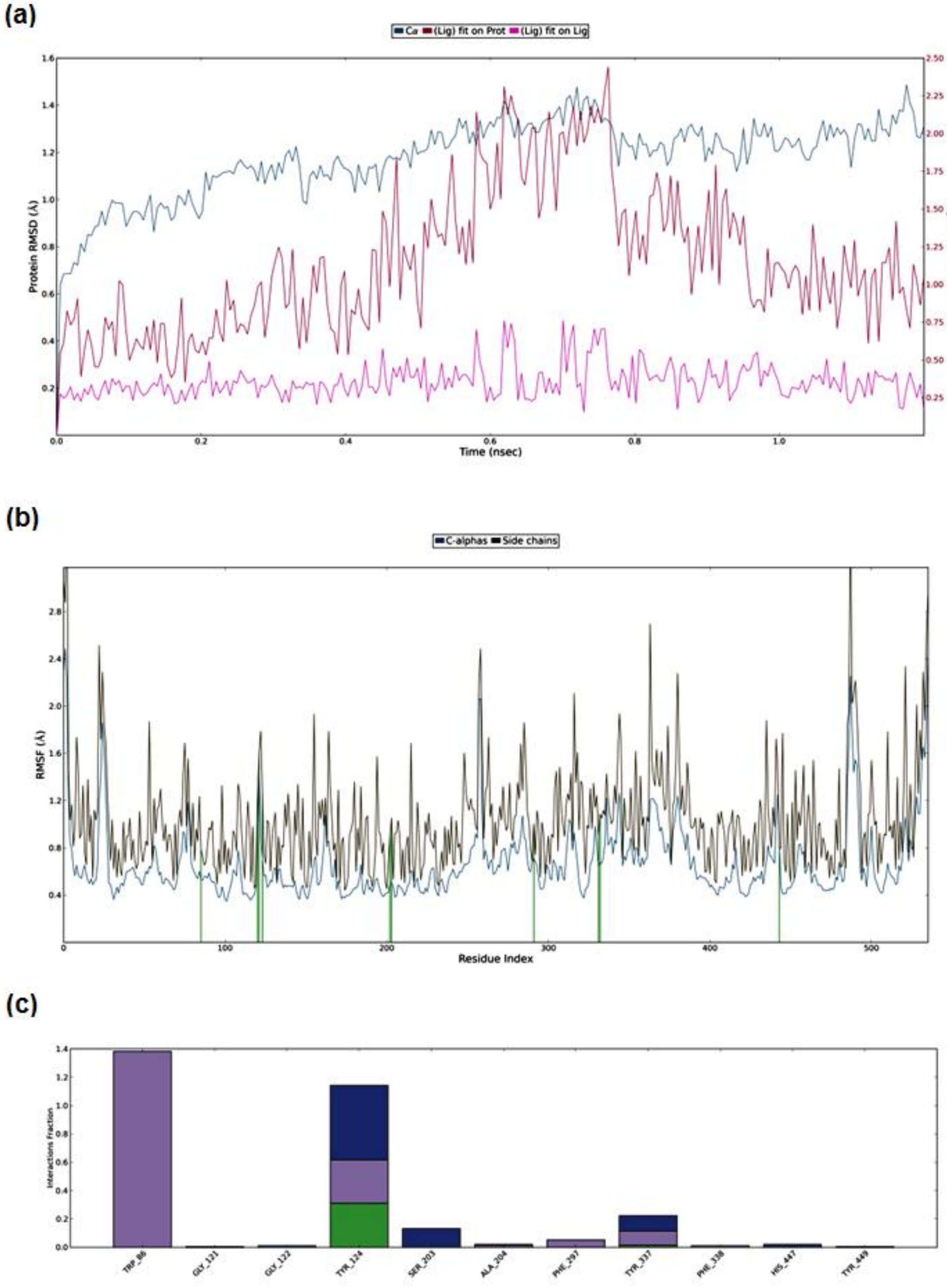

Figure S29. RMSDs (**a**), RMSFs (**b**), and Interaction diagrams (**c**) of *m*AChE in complex with diuron. The green bars present hydrogen bonds, the purple bars show hydrophobic interactions, the pink bars show ionic interaction, the blue bars present water bridges.

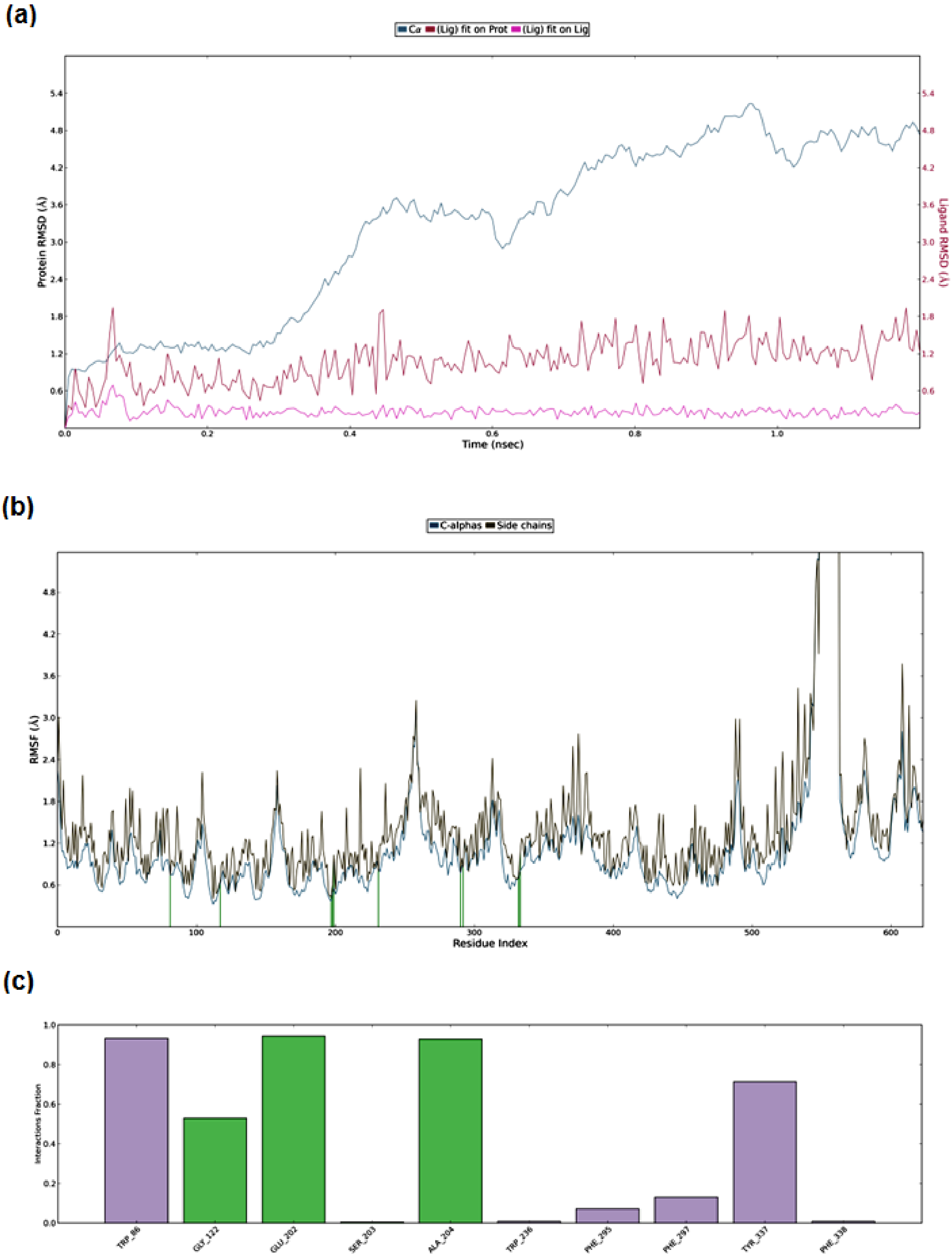

Figure S30. RMSDs (**a**), RMSFs (**b**), and Interaction diagrams (**c**) of *h*AChE in complex with diuron. The green bars present hydrogen bonds, the purple bars show hydrophobic interactions, the pink bars show ionic interaction, the blue bars present water bridges.

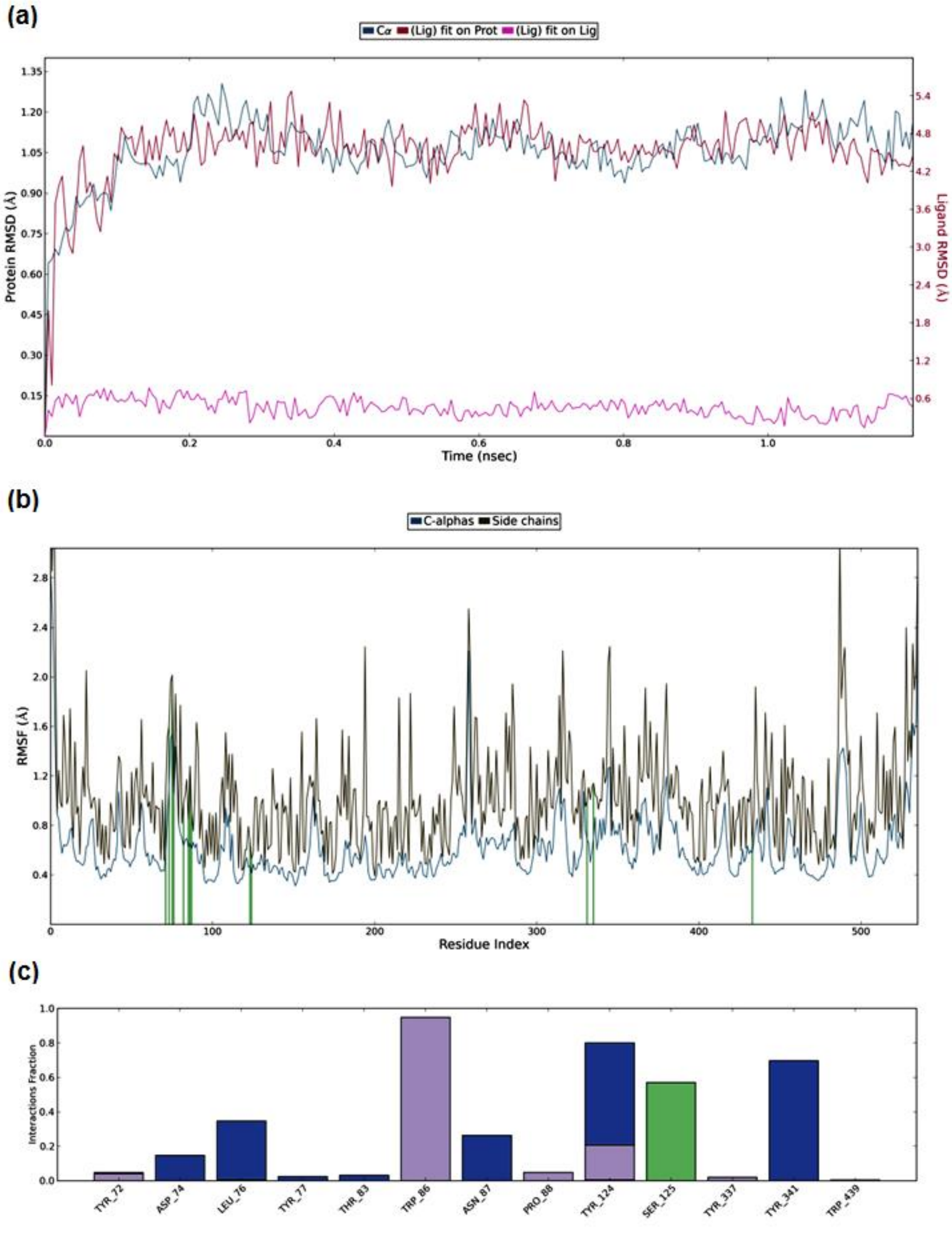

Figure S31. RMSDs (**a**), RMSFs (**b**), and Interaction diagrams (**c**) of *m*AChE in complex with monuron. The green bars present hydrogen bonds, the purple bars show hydrophobic interactions, the pink bars show ionic interaction, the blue bars present water bridges.

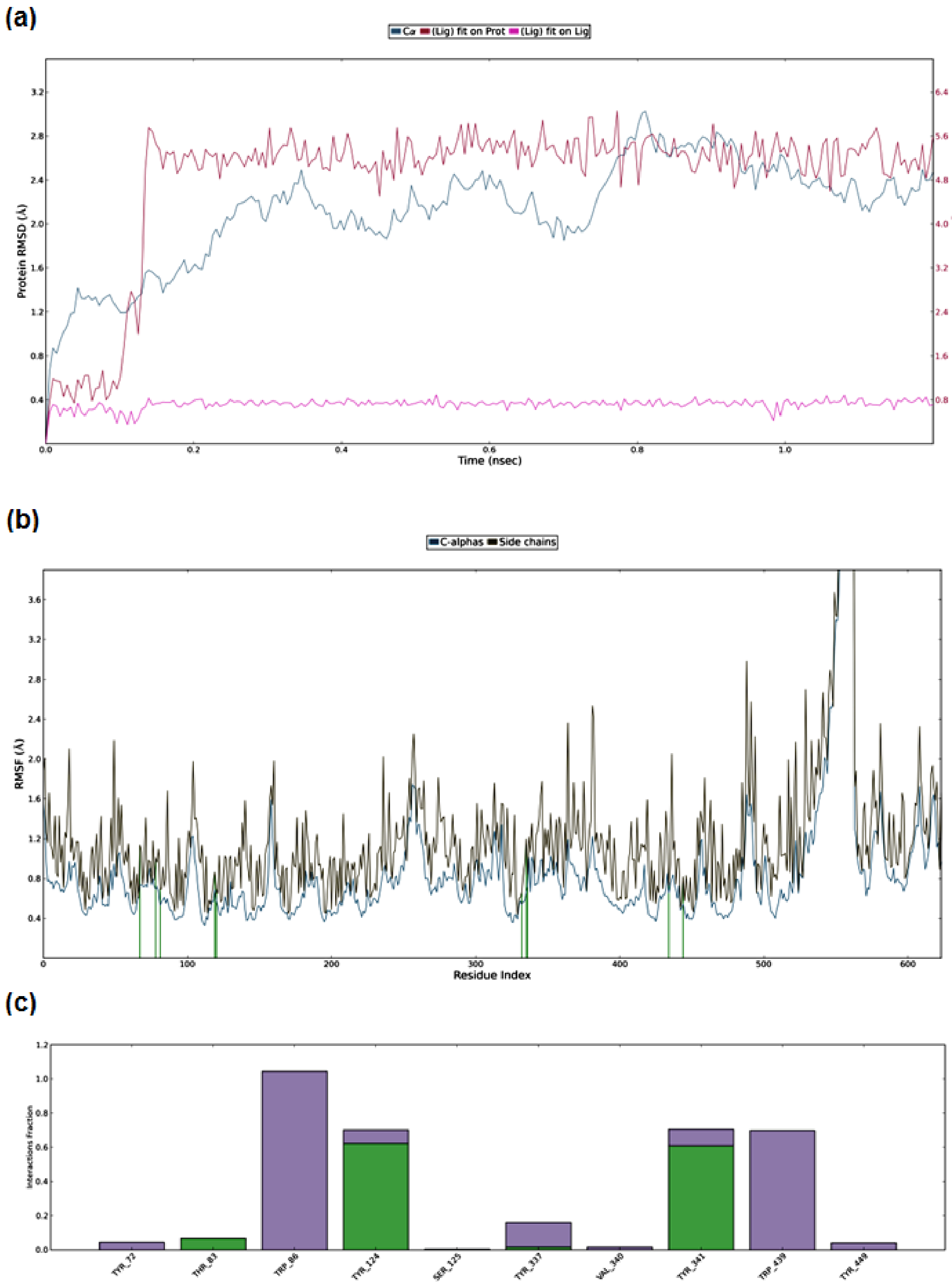

Figure S32. RMSDs (**a**), RMSFs (**b**), and Interaction diagrams (**c**) of *h*AChE in complex with monuron. The green bars present hydrogen bonds, the purple bars show hydrophobic interactions, the pink bars show ionic interaction, the blue bars present water bridges.

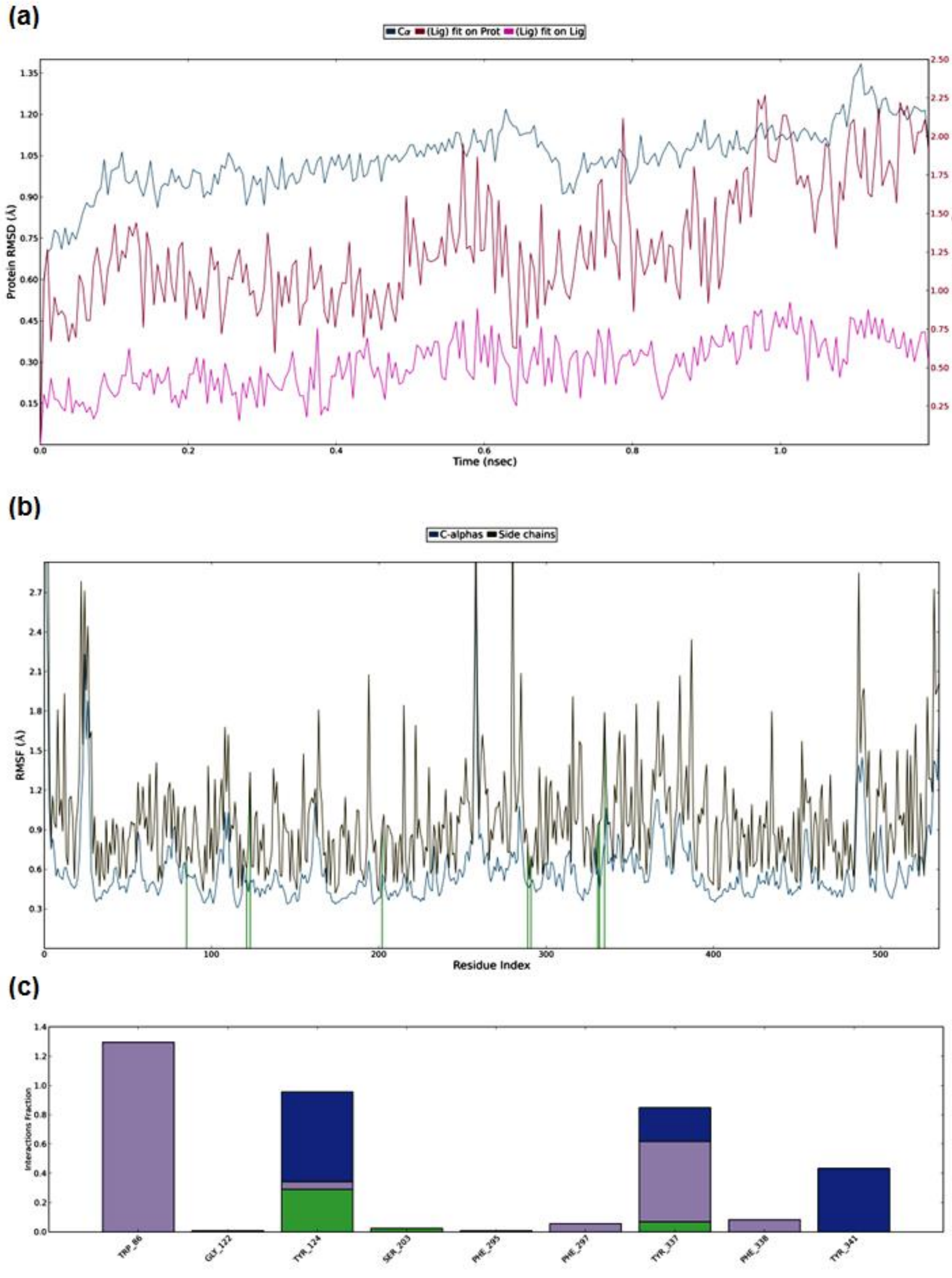

Figure S33. RMSDs (**a**), RMSFs (**b**), and Interaction diagrams (**c**) of *m*AChE in complex with linuron. The green bars present hydrogen bonds, the purple bars show hydrophobic interactions, the pink bars show ionic interaction, the blue bars present water bridges.

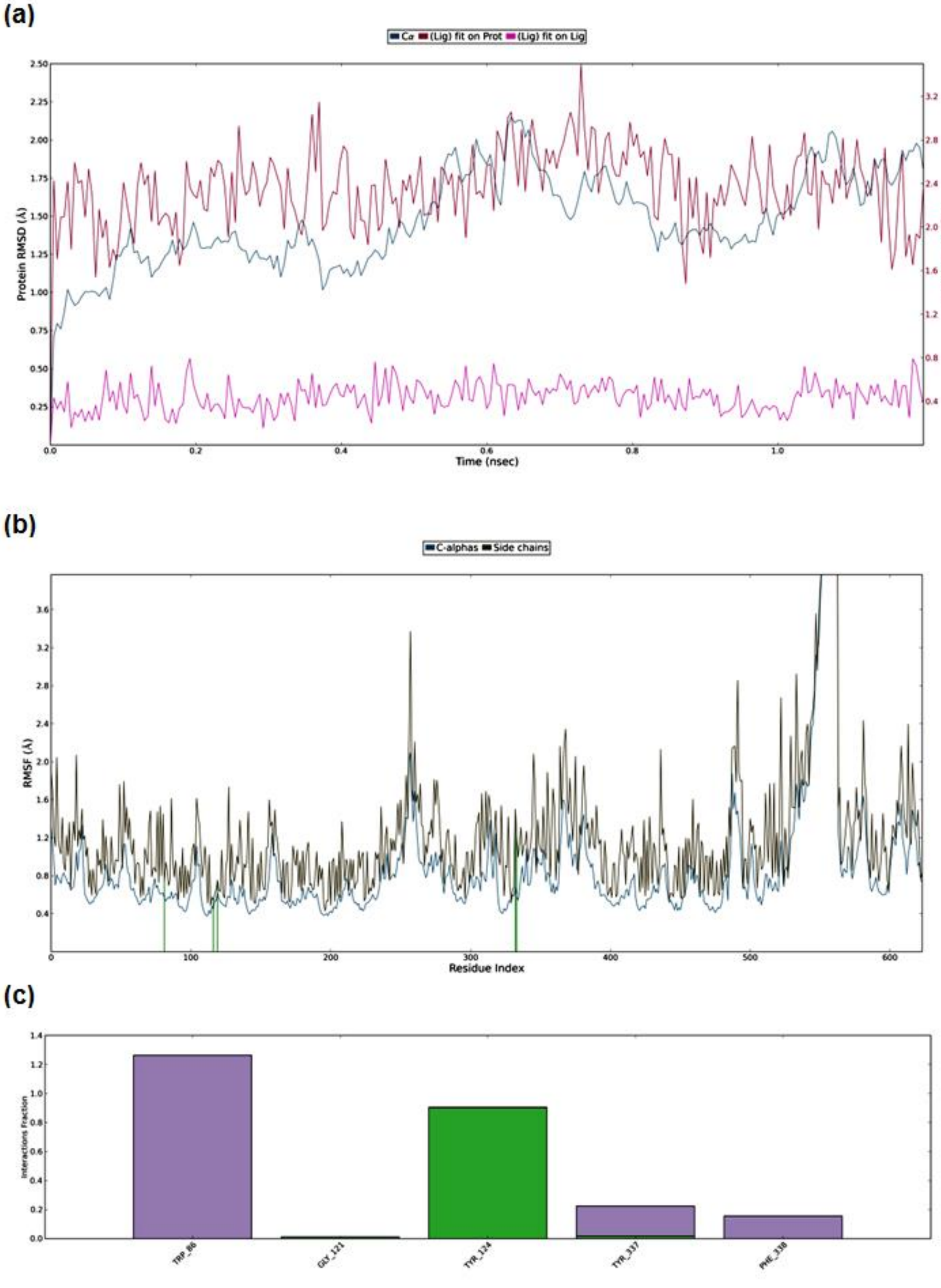

Figure S34. RMSDs (**a**), RMSFs (**b**), and Interaction diagrams (**c**) of *m*AChE in complex with linuron. The green bars present hydrogen bonds, the purple bars show hydrophobic interactions, the pink bars show ionic interaction, the blue bars present water bridges.

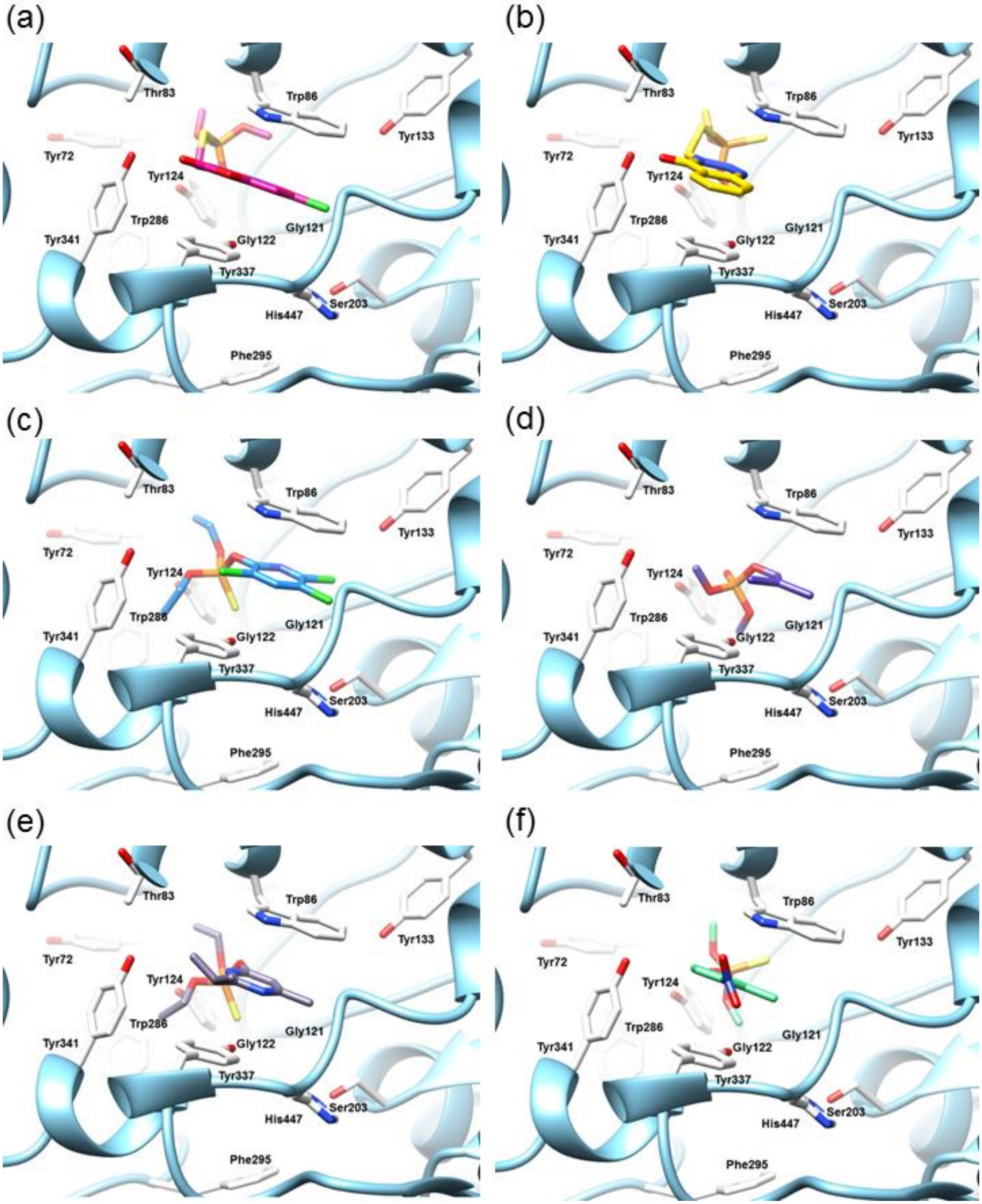

Figure S35. The SB alignment of azamethiphos (**a**), azinphos-methyl (**b**), chlorpyrifos (**c**), DDVP (**d**), diazinon (**e**), and fenitrothion (**f**) into the *m*AChE active site. The enzyme ribbons are presented in blue, active site amino acids are depicted in white. For the clarity purpose, hydrogen atoms are omitted from presentation.

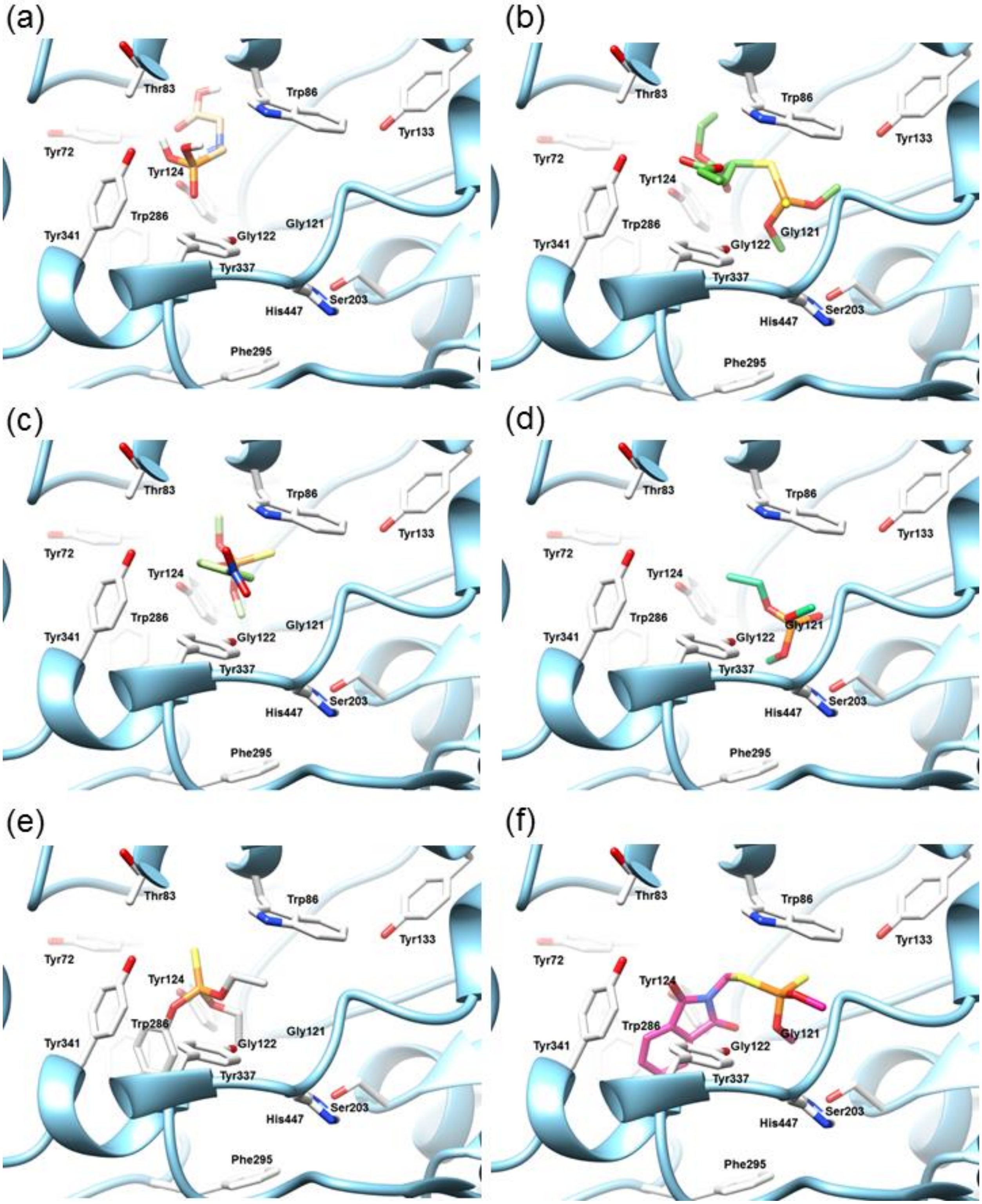

Figure S36. The SB alignment of glyphosate (**a**), malathion (**b**), methyl parathion (**c**), naled (dibrom) (**d**), parathion (**e**), and phosmet (**f**) into the *m*AChE active site. The enzyme ribbons are presented in blue, active site amino acids are depicted in white. For the clarity purpose, hydrogen atoms are omitted from presentation.

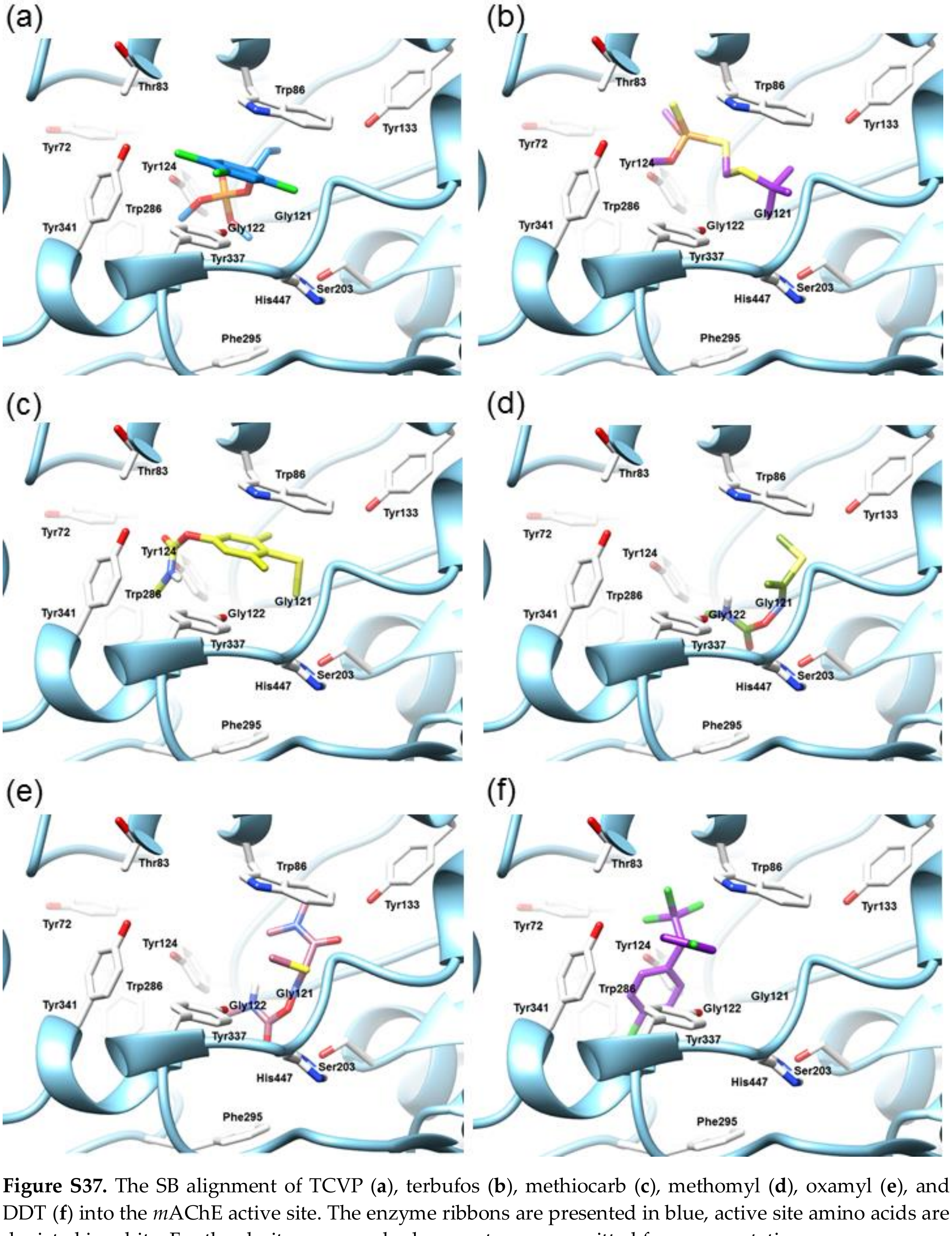

Figure S37. The SB alignment of TCVP (**a**), terbufos (**b**), methiocarb (**c**), methomyl (**d**), oxamyl (**e**), and DDT (**f**) into the *m*AChE active site. The enzyme ribbons are presented in blue, active site amino acids are depicted in white. For the clarity purpose, hydrogen atoms are omitted from presentation.

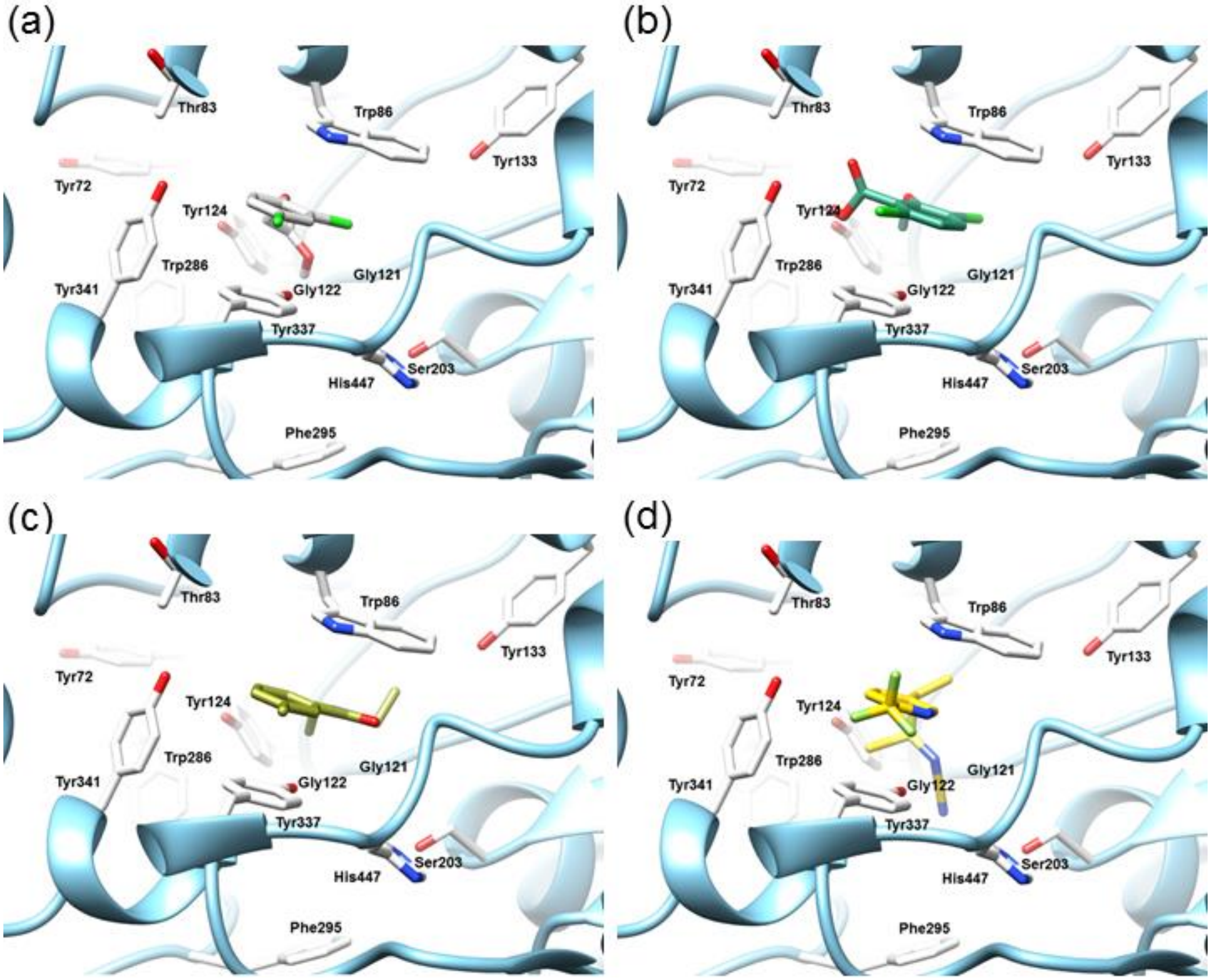

Figure S38. The SB alignment of 2,4-D (**a**), dicamba (**b**), DEET (**c**), and sulfoxaflor (**d**) into the *m*AChE active site. The enzyme ribbons are presented in blue, active site amino acids are depicted in white. For the clarity purpose, hydrogen atoms are omitted from presentation.

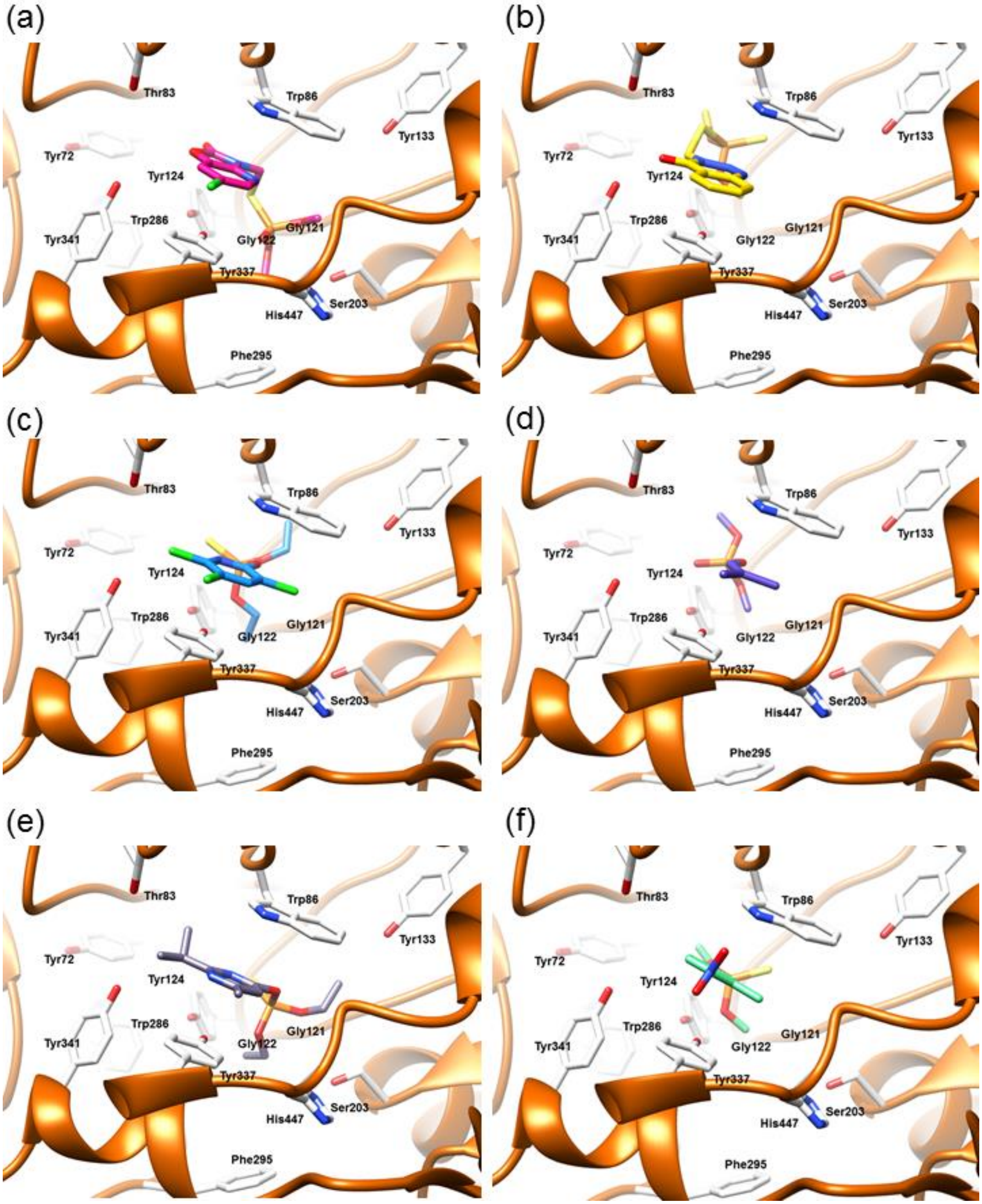

Figure S39. The SB alignment of azamethiphos (**a**), azinphos-methyl (**b**), chlorpyrifos (**c**), DDVP (**d**), diazinon (**e**), and fenitrothion (**f**), into the *h*AChE active site. The enzyme ribbons are presented in orange, active site amino acids are depicted in white. For the clarity purpose, hydrogen atoms are omitted from presentation.

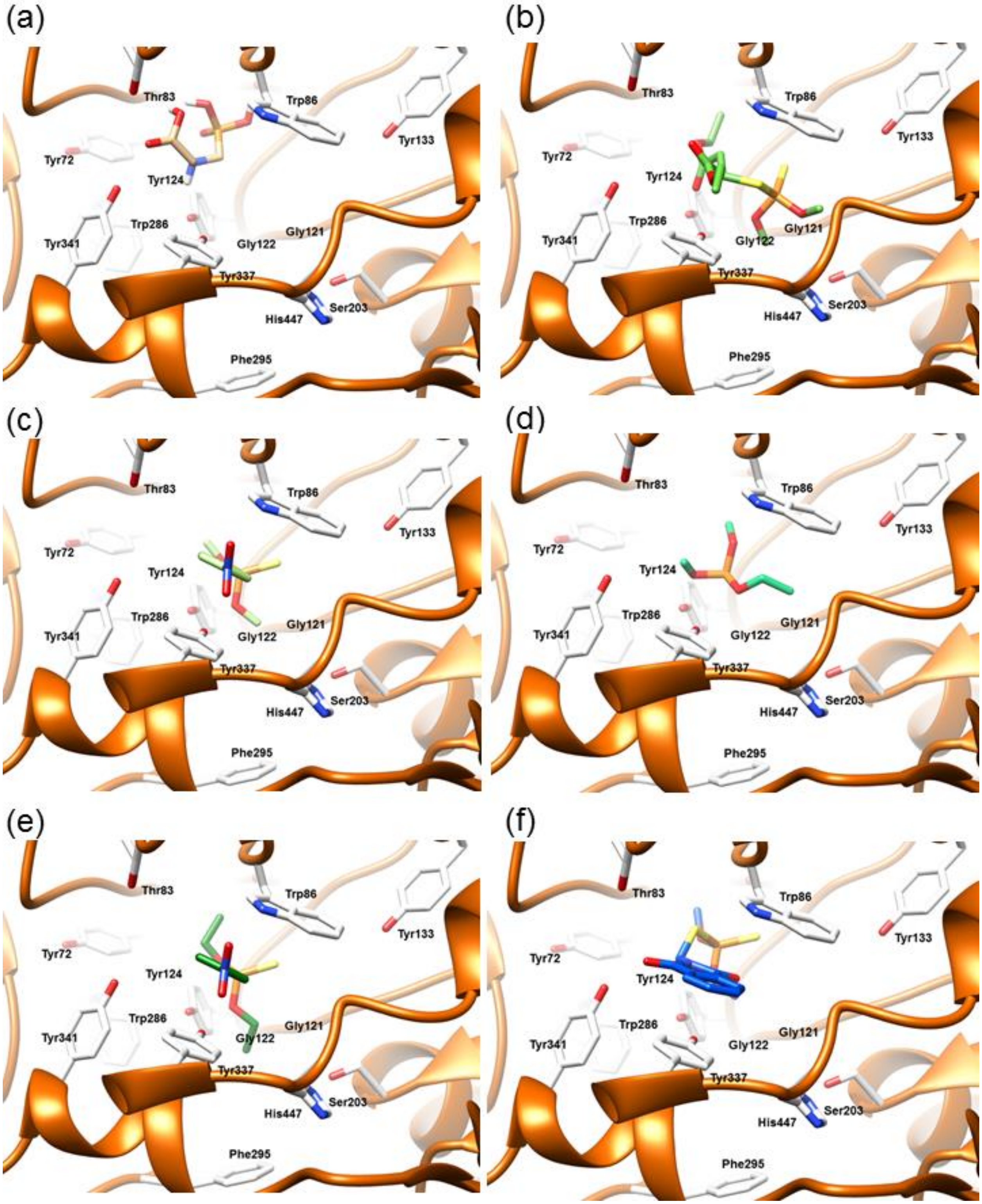

Figure S40. The SB alignment of glyphosate (**a**), malathion (**b**), methyl parathion (**c**), naled (dibrom) (**d**), parathion (**e**), and phosmet (**f**) into the *h*AChE active site. The enzyme ribbons are presented in orange, active site amino acids are depicted in white. For the clarity purpose, hydrogen atoms are omitted from presentation.

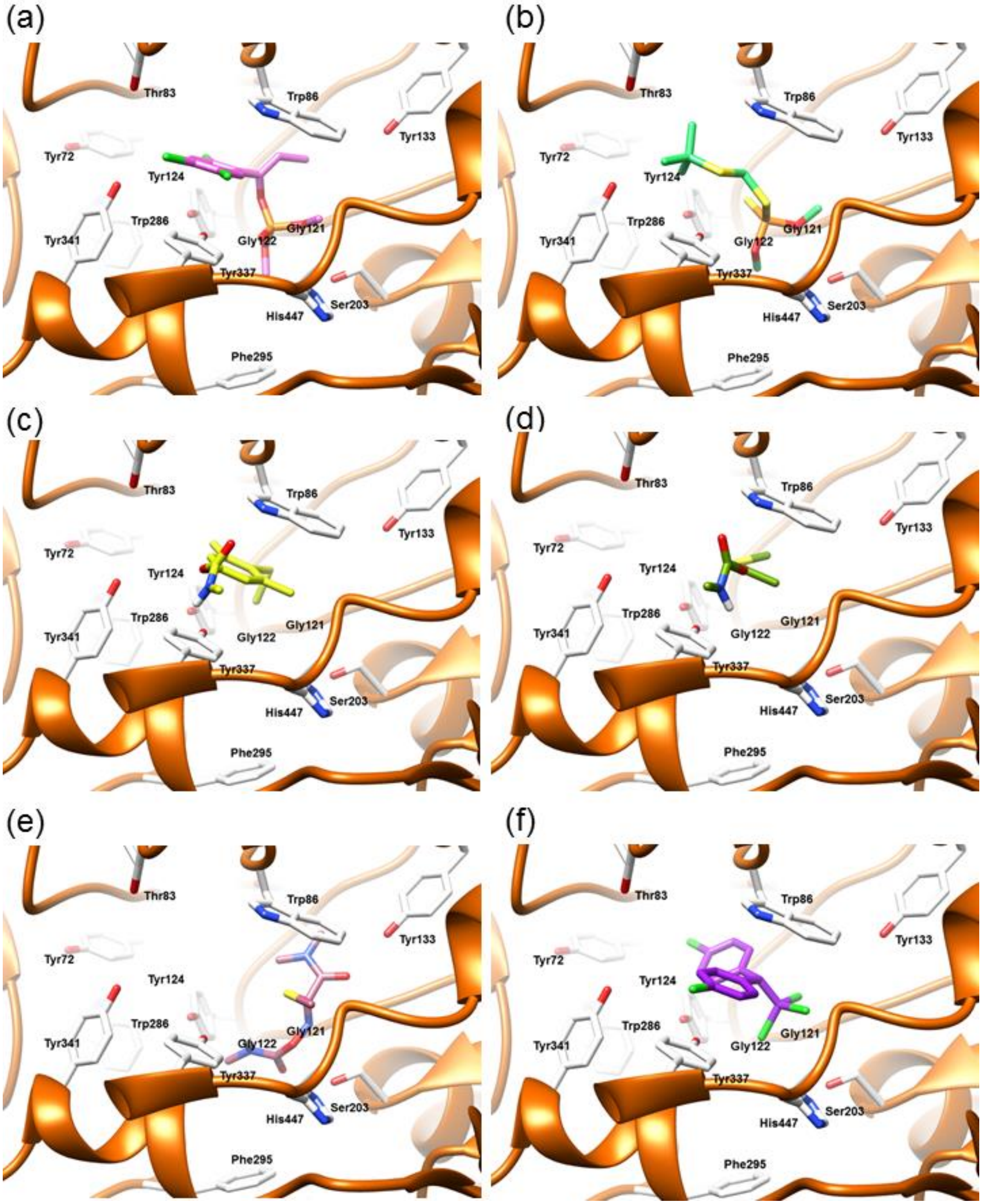

Figure S41. The SB alignment of TCVP (**a**), terbufos (**b**), methiocarb (**c**), methomyl (**d**), oxamyl (**e**), and DDT (**f**) into the *h*AChE active site. The enzyme ribbons are presented in blue, active site amino acids are depicted in white. For the clarity purpose, hydrogen atoms are omitted from presentation.

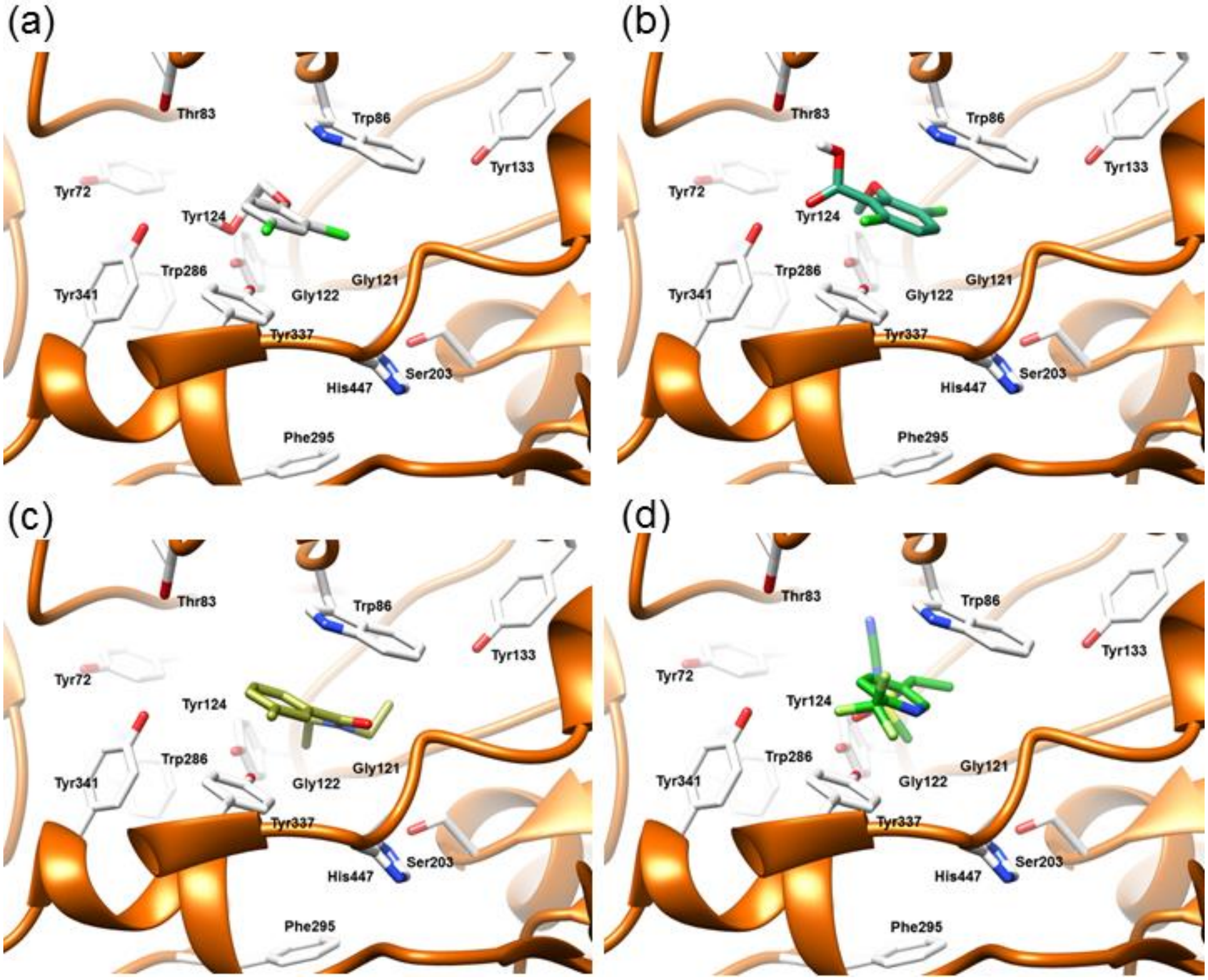

Figure S42. The SB alignment of 2,4-D (**a**), dicamba (**b**), DEET (**c**), and sulfoxaflor (**d**) into the *h*AChE active site. The enzyme ribbons are presented in orange, active site amino acids are depicted in white. For the clarity purpose, hydrogen atoms are omitted from presentation.

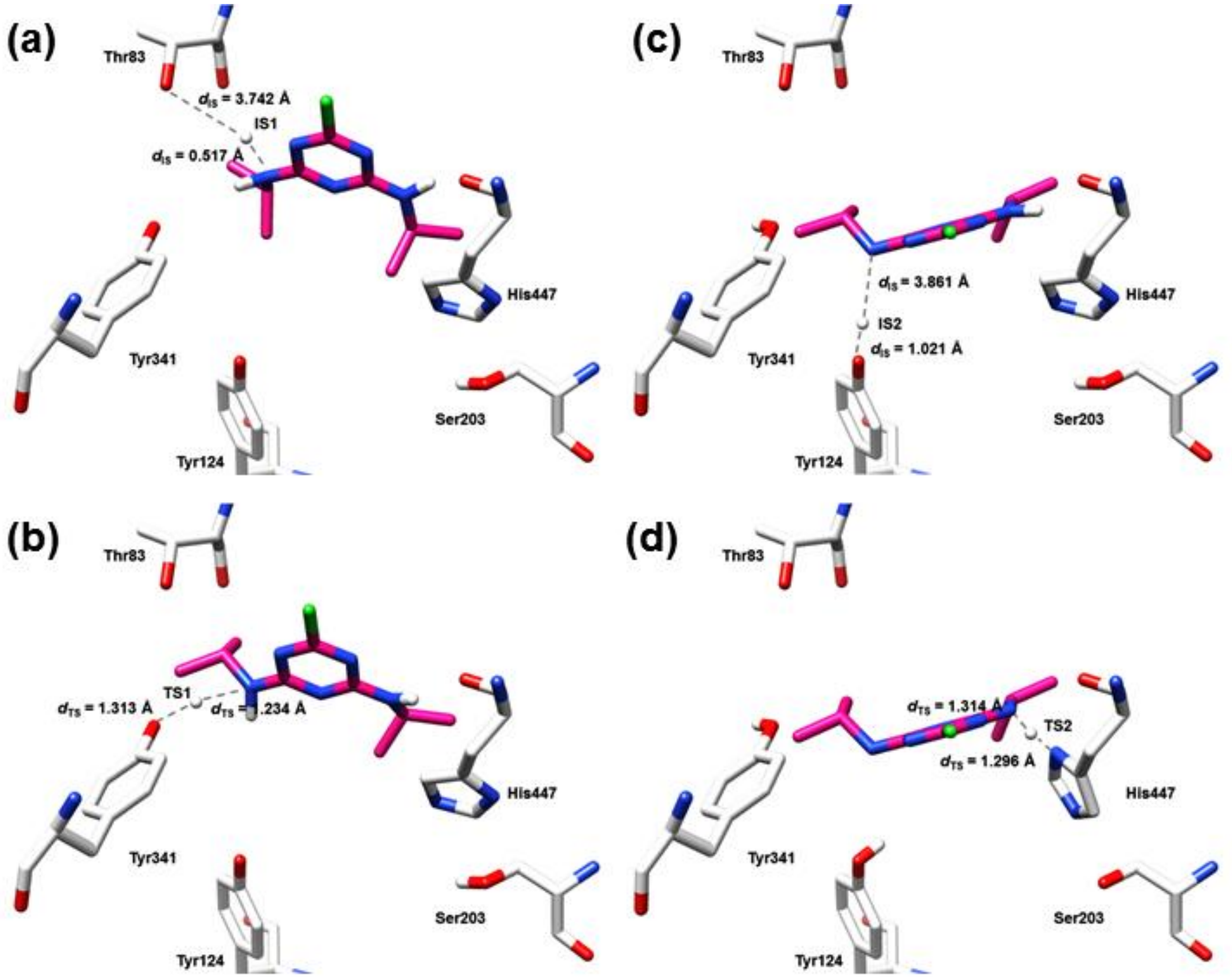

Figure S43. The quantum chemical mechanism of *Homo sapiens* acetylcholinesterase inhibition by propazine. The extracted geometry of IS1 (a); TS1 (b); IS2 (c); TS2 (d).

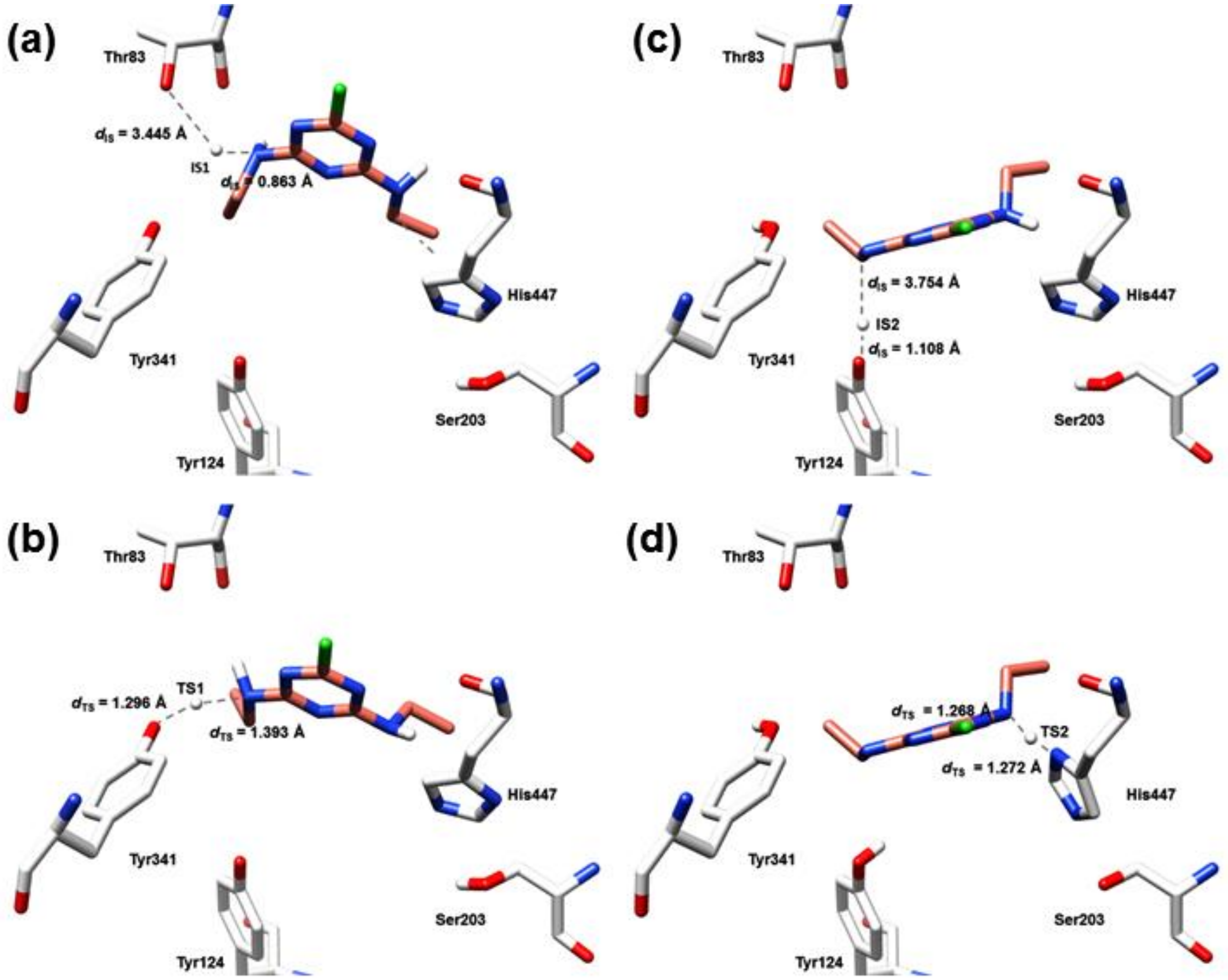

Figure S44. The quantum chemical mechanism of *Homo sapiens* acetylcholinesterase inhibition by simazine. The extracted geometry of IS1 (a); TS1 (b); IS2 (c); TS2 (d).

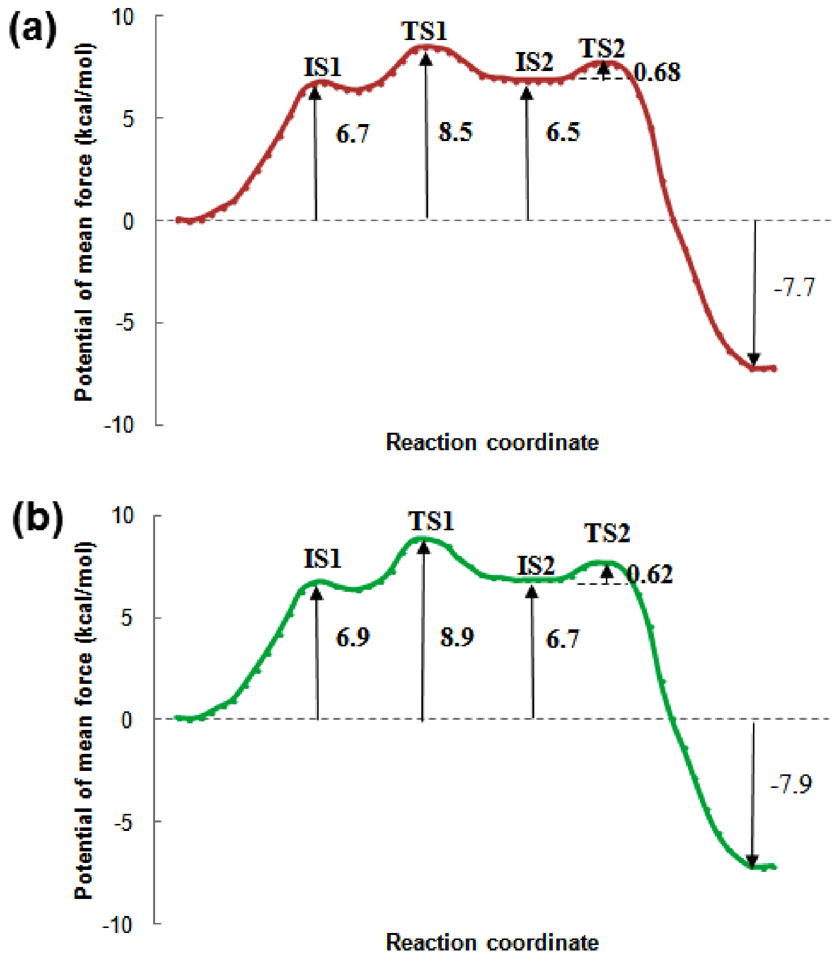

Figure S45. Free energy profile for *Homo sapiens* acetylcholinesterase inhibition by propazine (**a**) and simazine (**b**) by means of B3LYP (6-31G*) QM simulations.

Table S1. Training set pesticides chemical structures, conformational analysis, superposition of generated global minima using various force fields.

Pesticide	FF[a]	E_{glob_min}[b] (kJ/mol)	NGMS[c]	NF[d]	Pesticide alignment[e]	FFDAA[f] RMSD (Å)[g]
**acetylcholine**	MM3	-924.67	41	3026		MM3/MMFF
	AMBER 94	NA[h]	NA	NA		1.086
	MMFF	-992.47	45	3066		MM3/MMFFs
	MMFFs	-979.02	497	4897		1.703
	OPLSAA	-773.90	888	2075		MM3/OPLSAA
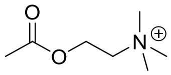					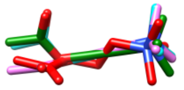	1.329
						MMFF/MMFFs
						1.165
						MMFF/OPLSAA
						1.004
						MMFFs/OPLSAA
						0.649
**atrazine**	MM3	-1161.48	445	23		MM3/MMFF
	AMBER 94	NA	NA	NA		0.892
	MMFF	-1007.32	147	62		MM3/MMFFs
	MMFFs	-992.63	509	20		0.931
	OPLSAA	-783.57	364	19		MM3/OPLSAA
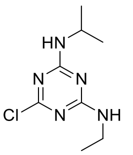					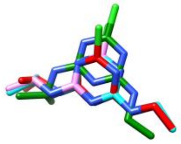	2.523
						MMFF/MMFFs
						0.343
						MMFF/OPLSAA
						2.487
						MMFFs/OPLSAA
						2.514
**propazine**	MM3	-1154.42	445	4370		MM3/MMFF
	AMBER 94	NA	NA	NA		6.346
	MMFF	-992.47	34	3131		MM3/MMFFs
	MMFFs	-979.02	577	4413		6.077
	OPLSAA	-773.9	877	2086		MM3/OPLSAA
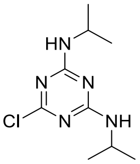					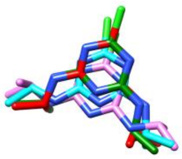	5.727
						MMFF/MMFFs
						2.092
						MMFF/OPLSAA
						2.842
						MMFFs/OPLSAA
						1.990
**simazine**	MM3	NA	NA	NA		MM3/MMFF
	AMBER 94	NA	NA	NA		NA
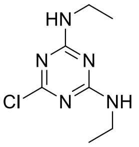	MMFF	-1022.18	136	2782		MM3/MMFFs
	MMFFs	NA	NA	NA		NA
	OPLSAA	-793.61	158	2306	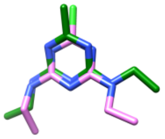	MM3/OPLSAA
						NA
						MMFF/MMFFs

NA
MMFF/OPLSAA
2.109
MMFFs/OPLSAA
NA

[a]Force field; [b]Energy of the global minimum; [c]Number of times that a single global minimum structure was found in 10000 processed structures; [d]Number of families *i.e.* different conformations found in 10000 processed structures; [e]Red - experimental conformation, Violet - MMFF conformation, Blue – MMFFs conformation, Green - OPLSAA conformation; [f]Force field dependent alignment accuracy; [g]RMSD measured between the heavy atoms of pesticides experimental and best performing force field conformations; [h]Not Available.

Table S2. Training set pesticides chemical structures, conformational analysis, superposition of generated global minima using various force fields.

Pesticide	FF[a]	E_{glob_min} [b] (kJ/mol)	NGMS[c]	NF[d]	Pesticide alignment[e]	FFDAA[f] RMSD (Å)[g]
**carbofuran**	MM3	29.60	3617	2		MM3/MMFF
	AMBER 94	NA[h]	NA	NA		NA
	MMFF	20.30	3307	2		MM3/MMFFs
	MMFFs	15.92	3384	2		NA
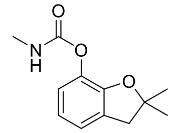	OPLSAA	73.27	5621	75	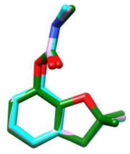	MM3/OPLSAA
						NA
						MMFF/MMFFs
						NA
						MMFF/OPLSAA
						2.109
						MMFFs/OPLSAA
						NA
**monocrotophos**	MM3	NA	NA	NA		MM3/MMFF
	AMBER 94	NA	NA	NA		NA
	MMFF	-307.36	241	62		MM3/MMFFs
	MMFFs	-310.26	218	61		NA
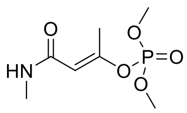	OPLSAA	NA	NA	NA	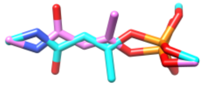	MM3/OPLSAA
						NA
						MMFF/MMFFs
						3.419
						MMFF/OPLSAA
						NA
						MMFFs/OPLSAA
						NA
**dimethoate**	MM3	NA	NA	NA		MM3/MMFF
	AMBER 94	NA	NA	NA		NA
	MMFF	-383.68	340	44		MM3/MMFFs
	MMFFs	-386.71	215	40		NA
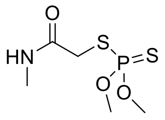	OPLSAA	-228.66	998	15	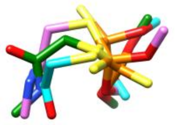	MM3/OPLSAA
						NA
						MMFF/MMFFs
						3.347
						MMFF/OPLSAA
						1.724
						MMFFs/OPLSAA
						3.328
**carbaryl**	MM3	-0.88	10000	1		MM3/MMFF
	AMBER 94	NA	NA	NA		2.019
	MMFF	20.43	10000	1		MM3/MMFFs
	MMFFs	15.66	10000	1		2.022
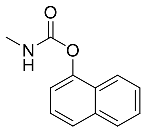	OPLSAA	59.34	9986	1	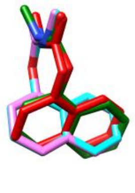	MM3/OPLSAA
						0.454
						MMFF/MMFFs

						0.088
						MMFF/OPLSAA
						1.957
						MMFFs/OPLSAA
						2.514
**tebufenozide**	MM3	NA	NA	NA		MM3/MMFF
	AMBER 94	NA	NA	NA		NA
	MMFF	467.38	1759	4		MM3/MMFFs
	MMFFs	470.26	1799	4		NA
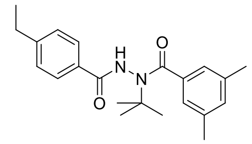	OPLSAA	13.33	1474	8	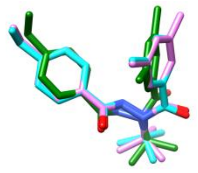	MM3/OPLSAA
						NA
						MMFF/MMFFs
						0.370
						MMFF/OPLSAA
						2.769
						MMFFs/OPLSAA
						2.686

[a]Force field; [b]Energy of the global minimum; [c]Number of times that a single global minimum structure was found in 10000 processed structures; [d]Number of families *i.e.* different conformations found in 10000 processed structures; [e]Red - experimental conformation, Violet - MMFF conformation, Blue – MMFFs conformation, Green - OPLSAA conformation; [f]Force field dependent alignment accuracy; [g]RMSD measured between the heavy atoms of pesticides experimental and best performing force field conformations; [h]Not Available.

Table S3. Training set pesticides chemical structures, conformational analysis, superposition of generated global minima using various force fields.

Pesticide	FF[a]	E_{glob_min}[b] (kJ/mol)	NGMS[c]	NF[d]	Pesticide alignment[e]	FFDAA[f] RMSD (Å)[g]
**imidacloprid**	MM3	NA[h]	NA	NA		MM3/MMFF NA
	AMBER 94	NA	NA	NA		MM3/MMFFs NA
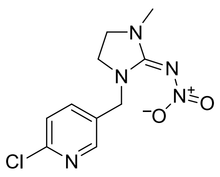	MMFF	-168.00	1027	5	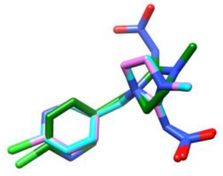	MM3/OPLSAA NA
	MMFFs	-165.41	3643	3		MMFF/MMFFs 0.122
	OPLSAA	90.86	365	23		MMFF/OPLSAA 3.282
						MMFFs/OPLSAA 3.277
**acetamiprid**	MM3	NA	NA	NA		MM3/MMFF NA
	AMBER 94	NA	NA	NA		MM3/MMFFs NA
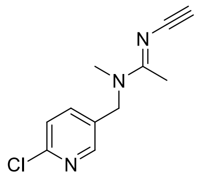	MMFF	-31.45	1916	14	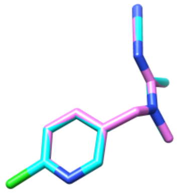	MM3/OPLSAA NA
	MMFFs	-31.29	1816	6		MMFF/MMFFs 0.041
	OPLSAA	NA	NA	NA		MMFF/OPLSAA NA
						MMFFs/OPLSAA NA
**diuron**	MM3	-37.27	3108	2		MM3/MMFF 1.886
	AMBER 94	NA	NA	NA		MM3/MMFFs 1.882
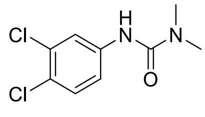	MMFF	-142.96	2537	2	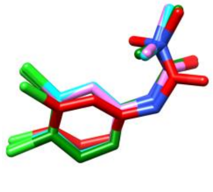	MM3/OPLSAA 1.899
	MMFFs	-140.51	2523	2		MMFF/MMFFs 0.121
	OPLSAA	-3.52	2908	2		MMFF/OPLSAA 0.399
						MMFFs/OPLSAA 0.330
**monuron**	MM3	-76.13	10000	1		MM3/MMFF 0.533
	AMBER 94	NA	NA	NA		MM3/MMFFs 2.004
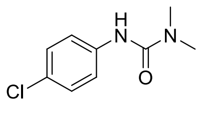	MMFF	-197.63	10000	1	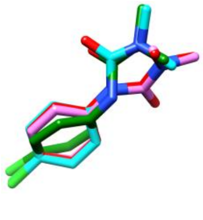	MM3/OPLSAA 2.629
	MMFFs	-199.43	10000	1		MMFF/MMFFs
	OPLSAA	-74.72	7691	2		

						2.049
						MMFF/ OPLSAA
						2.695
						MMFFs/OPLSAA
						2.032
**linuron**	MM3	NA	NA	NA		MM3/MMFF
	AMBER 94	NA	NA	NA		3.597
	MMFF	-992.47	45	3066		MM3/MMFFs
	MMFFs	-979.02	497	4897		3.574
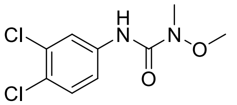	OPLSAA	-773.90	888	2075	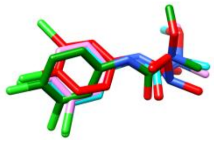	MM3/OPLSAA
						2.951
						MMFF/MMFFs
						0.523
						MMFF/OPLSAA
						1.833
						MMFFs/OPLSAA
						1.889

[a]Force field; [b]Energy of the global minimum; [c]Number of times that a single global minimum structure was found in 10000 processed structures; [d]Number of families *i.e.* different conformations found in 10000 processed structures; [e]Red - experimental conformation, Violet - MMFF conformation, Blue – MMFFs conformation, Green - OPLSAA conformation; [f]Force field dependent alignment accuracy; [g]RMSD measured between the heavy atoms of pesticides experimental and best performing force field conformations; [h]Not Available.

Table S4. Test set pesticides chemical structures and obtained global minima using MMFF as the best performing force field.

Pesticide	E_{glob_min}[a] (kJ/mol)	Conformation	Pesticide	E_{glob_min}[a] (kJ/mol)	Conformation
**azamethiphos** 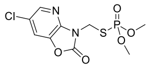	-561.48	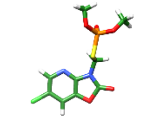	**phosmet** 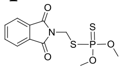	-549.21	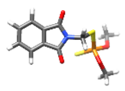
**azinphos-methyl** 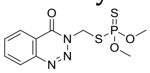	-361.00	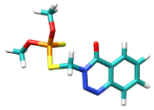	**TCVP** 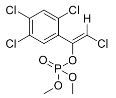	-130.74	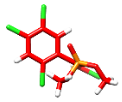
**chlorpyrifos** 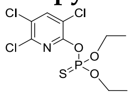	-247.37	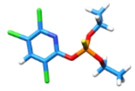	**terbufos** 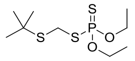	-382.19	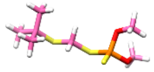
**DDVP** 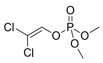	-214.58	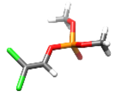	**methiocarb** 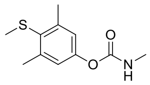	-75.96	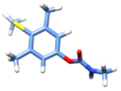
**diazinon** 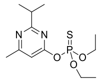	-274.36	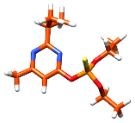	**methomyl** 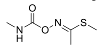	-72.25	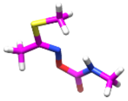
**fenitrothion** 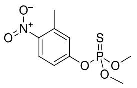	-45.79	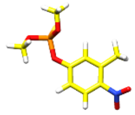	**oxamyl** 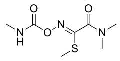	118.32	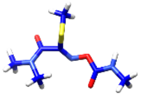
**glyphosate** 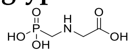	-221.48	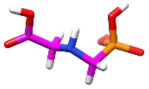	**DDT** 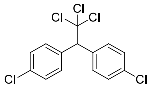	352.49	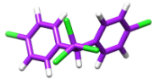
**malathion** 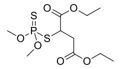	-498.14	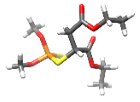	**2,4-D** 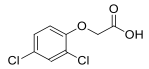	28.66	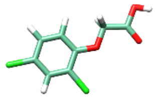
**methyl parathion** 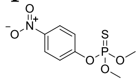	-74.64	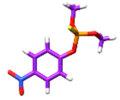	**dicamba** 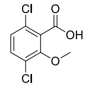	63.27	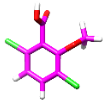
**naled (dibrom)** 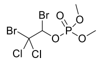	-337.18		**DEET** 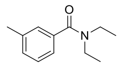	70.17	
**parathion** 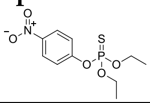	-90.35		**sulfoxaflor** 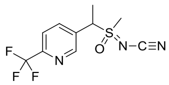	178.42	

[a] $E_{global\_minimum}$

Table S5. Linear regression parameters for QSAR model 2 (acute toxicity against *Mus musculus*).

Compound	LD_{50} (mg/kg)	MW	E_{glob_min} MMFF (kJ/mol)	Exp. pLD_{50}	Fitted pLD_{50}
**atrazine**	0.85	215.69	-1007.3	5.40	4.28
**propazin**	3.18	229.71	-992.47	4.86	4.26
**simazine**	5	201.66	-1022.2	4.61	4.30
**carbofuran**	2	221.11	20.3	5.04	3.09
**monocrotophos**	14	223.16	-307.36	4.20	3.47
**dimethoate**	60	229.25	-383.68	3.58	3.56
**carbaryl**	100	201.22	20.43	3.30	3.09
**tebufenozide**	5000	352.48	467.38	1.85	2.58
**imidacloprid**	131	269.69	-168	3.31	3.31
**acetamiprid**	184	221.69	-31.45	3.08	3.15
**diuron**	500	233.09	-142.96	2.67	3.28
**monuron**	1700	198.65	-197.63	2.07	3.35
**linuron**	2400	249.09	-992.47	2.02	4.26

Table S6. External validation of QSAR model 2 (acute toxicity against *Mus musculus*).

Compound	LD_{50} (mg/kg)	MW	E_{glob_min} MMFF (kJ/mol)	Exp. pLD_{50}	Fitted pLD_{50}
**azamethiphos**	1040.00	324.68	-561.48	4.07	1.98
**azinphos-methyl**	7.00	317.32	-361.00	6.25	2.36
**chlorpyrifos**	2000.00	350.59	-247.37	3.79	2.58
**DDVP**	17.00	220.98	-214.58	5.86	2.64
**diazinon**	66.00	304.35	-274.36	5.27	2.53
**fenitrothion**	500.00	277.23	-45.79	4.39	2.96
**glyphosate**	5000.00	169.07	-221.48	3.39	2.63
**malathion**	290.00	330.36	-498.14	4.63	2.10
**methyl parathion**	18.00	263.21	-74.64	5.84	2.91
**naled (dibrom)**	160.00	380.78	-337.18	4.89	2.41
**parathion**	2.00	291.26	-90.35	6.79	2.88
**phosmet**	113.00	317.32	-549.21	5.04	2.01
**TCVP**	465.00	365.96	-130.74	4.42	2.80
**terbufos**	1.60	288.43	-382.19	6.89	2.32
**methiocarb**	350.00	225.31	-75.96	4.55	2.91
**methomyl**	12.00	162.21	-72.25	6.01	2.91
**oxamyl**	5.40	219.26	118.32	6.36	3.27
**DDT**	113.00	354.49	352.49	5.04	3.72
**2,4-D**	639.00	221.04	28.66	4.29	3.10
**dicamba**	757.00	221.04	63.27	4.21	3.17
**DEET**	1.95	191.27	70.17	6.80	3.18
**sulfoxaflor**	750.00	277.27	178.42	4.22	3.39

Table S7. Linear regression parameters for QSAR model 4 (acute toxicity against *Homo sapiens*).

Compound	LD_{50} (mg/kg)	MW	E_{glob_min} MMFF (kJ/mol)	Calculated pLD_{50}	Fitted pLD_{50}
**atrazine**	0.07	215.69	-1007.30	6.50	5.52
**propazin**	0.26	229.71	-992.47	5.95	5.21
**simazine**	0.41	201.66	-1022.20	5.70	4.74
**carbofuran**	0.16	221.11	20.30	6.14	5.13
**monocrotophos**	1.14	223.16	-307.36	5.29	4.34
**dimethoate**	4.86	229.25	-383.68	4.67	4.10
**carbaryl**	8.11	201.22	20.43	4.39	3.71
**tebufenozide**	405.40	352.48	467.38	2.94	1.98
**imidacloprid**	10.62	269.69	-168.00	4.40	3.95
**acetamiprid**	14.92	221.69	-31.45	4.17	3.63
**diuron**	40.54	233.09	-142.96	3.76	3.00
**monuron**	137.84	198.65	-197.63	3.16	2.84
**linuron**	194.59	249.09	-992.47	3.11	2.21

Table S8. External validation of QSAR model 4 (acute toxicity against *Homo sapiens*).

Compound	LD_{50} (mg/kg)	MW	E_{glob_min} MMFF (kJ/mol)	Calculated pLD_{50}	Fitted pLD_{50}
**azamethiphos**	1040.00	324.68	-561.48	2.49	4.10
**azinphos-methyl**	7.00	317.32	-361.00	4.66	4.62
**chlorpyrifos**	2000.00	350.59	-247.37	2.24	3.48
**DDVP**	17.00	220.98	-214.58	4.11	4.02
**diazinon**	66.00	304.35	-274.36	3.66	4.03
**fenitrothion**	500.00	277.23	-45.79	2.74	4.57
**glyphosate**	5000.00	169.07	-221.48	1.53	5.10
**malathion**	290.00	330.36	-498.14	3.06	4.29
**methyl parathion**	18.00	263.21	-74.64	4.17	4.79
**naled (dibrom)**	160.00	380.78	-337.18	3.38	3.81
**parathion**	2.00	291.26	-90.35	5.16	4.40
**phosmet**	113.00	317.32	-549.21	3.45	4.32
**TCVP**	465.00	365.96	-130.74	2.90	4.29
**terbufos**	1.60	288.43	-382.19	5.26	4.67
**methiocarb**	350.00	225.31	-75.96	2.81	4.56
**methomyl**	12.00	162.21	-72.25	4.13	5.19
**oxamyl**	5.40	219.26	118.32	4.61	4.24
**DDT**	113.00	354.49	352.49	3.50	5.22
**2,4-D**	639.00	221.04	28.66	2.54	4.84
**dicamba**	757.00	221.04	63.27	2.47	4.88
**DEET**	1.95	191.27	70.17	4.99	4.29
**sulfoxaflor**	750.00	277.27	178.42	2.57	3.93

Table S9. Structure-based alignment assessment for wild type *Mus musculus* AChE inhibitors.

Ligand PDB	Ref.	AutoDock BD[a]	AutoDock BC[b]	Vina	DOCK6 R[c]	DOCK6 F[d]	AutoDock BD[a]	AutoDock BC[b]	Vina	DOCK6 R[c]	DOCK6 F[d]
		Experimental Conformation Re-Docking					*Randomized Conformation Re-Docking*				
**1J07**	[SR1]	17.236	18.232	13.274	21.343	22.768	18.396	18.432	16.728	22.341	22.761
**1N5M**	[SR1]	3.942	4.231	4.241	5.941	6.928	3.726	3.246	5.921	6.314	7.043
**1N5R**	[SR1]	2.767	3.243	1.928	3.841	3.992	3.242	3.531	2.941	4.323	4.447
**1Q83**	[SR2]	2.072	2.576	1.248	2.761	2.997	2.437	3.231	1.764	3.347	3.471
**1Q84**	[SR2]	1.076	1.224	0.692	4.271	5.226	1.574	2.248	1.248	5.176	5.449
**2C0P**	[SR3]	7.321	8.246	5.494	9.274	10.111	8.321	9.326	6.724	12.471	13.226
**2C0Q**	[SR3]	8.436	9.072	6.726	11.246	13.276	8.773	9.323	2.892	14.178	14.326
**2GYU**	[SR4]	3.941	3.893	1.547	2.748	2.673	4.276	4.832	1.663	3.437	3.543
**2GYV**	[SR4]	2.996	2.934	1.894	2.896	2.937	2.993	2.837	1.893	3.324	3.333
**2GYW**	[SR4]	2.534	2.637	1.747	2.731	2.834	2.539	2.639	1.767	2.076	2.113
**2H9Y**	[SR5]	3.121	3.276	2.793	7.394	7.776	4.214	5.432	2.992	9.317	10.116
**2HA0**	[SR5]	1.974	2.127	2.432	3.293	4.727	2.126	3.213	2.748	5.471	5.557
**2JEY**	[SR6]	1.006	1.118	2.947	5.436	5.445	1.763	1.846	2.894	1.839	1.937
**2JEZ**	[SR6]	1.872	1.936	2.846	4.373	4.437	2.793	2.937	2.993	1.993	1.973
**2JF0**	[SR6]	2.034	2.122	2.373	3.391	4.937	2.184	2.617	2.446	1.643	1.643
**2JGI**	[SR7]	1.993	1.973	8.476	1.935	2.317	2.932	3.143	8.562	4.374	4.551
**2WHP**	[SR8]	2.967	2.939	1.747	1.993	2.074	3.327	3.446	1.754	1.236	1.279
**2WHQ**	[SR8]	2.224	2.517	1.647	2.851	2.935	3.247	3.536	1.673	1.973	1.984
**2WHR**	[SR8]	1.973	1.868	1.993	2.661	2.639	1.831	1.884	1.961	1.837	1.883
**2WU3**	[SR9]	2.831	2.993	2.713	2.743	2.839	3.936	3.974	2.831	2.532	2.631
**2WU4**	[SR9]	3.296	3.348	2.262	2.276	2.374	4.333	4.536	2.341	2.617	2.814
**3ZLT**	[SR10]	1.276	2.574	1.894	2.761	2.432	2.574	3.473	1.964	2.714	2.971
**3ZLU**	[SR10]	1.543	1.673	1.627	2.641	3.241	2.313	3.116	1.892	2.774	2.931
**3ZLV**	[SR10]	2.056	2.157	1.942	3.417	3.776	2.746	3.547	1.972	2.561	2.549
**4A16**	[SR11]	1.478	1.723	0.712	3.278	3.796	1.536	1.928	1.942	2.736	2.931
**4A23**	[SR12]	4.334	4.593	2.347	2.459	2.547	4.763	4.863	2.492	3.913	3.977
**4ARA**	[SR12]	4.328	4.446	1.336	2.583	2.593	4.331	4.371	1.368	2.031	2.213
**4ARB**	[SR12]	3.287	3.371	0.946	1.943	2.074	4.328	4.463	1.032	1.224	1.237
**4B7Z**	[SR13]	1.924	3.896	2.614	3.293	3.326	1.983	1.976	2.684	2.546	2.661
**4B80**	[SR13]	1.791	1.931	2.761	2.791	3.763	1.883	1.936	2.781	3.073	3.116
**4B81**	[SR13]	2.554	2.668	2.663	3.291	3.394	2.439	2.636	2.669	2.837	2.891
**4B82**	[SR13]	2.437	2.673	3.617	8.639	8.743	2.693	2.721	3.613	4.116	4.227
**4B83**	[SR13]	3.839	3.937	2.461	4.263	4.638	4.261	2.274	2.531	2.674	2.668
**4B84**	[SR13]	2.673	2.769	3.334	4.293	4.398	3.296	3.297	3.557	4.132	4.339
**4B85**	[SR13]	2.553	2.663	2.637	5.324	5.371	2.583	2.661	2.745	3.113	3.273
**4BC0**	[SR14]	2.931	2.537	1.763	1.283	1.334	2.971	3.046	1.893	2.743	2.663
**4BC1**	[SR14]	2.671	2.761	1.831	1.713	1.831	2.772	2.884	1.884	1.743	1.838
**5DTJ**	[SR15]	4.231	4.447	2.398	6.341	6.477	8.392	10.326	2.576	2.838	3.091
**5EHN**	[SR16]	3.928	3.936	6.893	5.432	5.543	4.326	4.436	7.452	6.831	6.373
**5EHQ**	[SR16]	4.331	4.749	5.437	5.448	5.793	3.298	3.393	4.631	5.346	5.519
**5EHZ**	[SR16]	4.237	4.563	4.831	6.831	6.939	4.237	4.117	4.448	3.931	4.126
**5EIA**	[SR16]	6.891	6.661	11.839	5.237	5.831	6.921	6.974	12.903	9.313	9.884
**5EIE**	[SR16]	7.327	6.971	8.396	8.329	8.437	7.432	8.063	8.996	7.439	7.661

**5EIH**	[SR16]	1.561	1.663	1.941	8.883	8.736	1.763	1.833	1.893	2.354	2.931
**5HCU**	[SR15]	1.229	1.291	1.836	7.329	7.638	1.339	1.354	1.884	2.551	2.689
DA		44.55%	40%	55.55	25.55%	20%	34.44%	26.67%	57.78%	28.89%	30%
		Experimental Conformation Cross-Docking					*Randomized Conformation Cross-Docking*				
**1J07**	[SR1]	22.732	24.328	16.872	24.371	25.293	24.372	25.761	22.841	24.873	25.297
**1N5M**	[SR1]	5.231	5.432	7.241	7.341	8.226	6.393	8.426	9.326	7.891	8.137
**1N5R**	[SR1]	3.213	3.738	3.296	4.374	4.448	4.761	4.923	5.841	5.441	5.761
**1Q83**	[SR2]	3.076	3.214	2.741	4.371	4.439	3.573	3.926	4.321	5.484	5.716
**1Q84**	[SR2]	2.327	2.438	1.639	3.917	3.938	3.117	3.234	2.276	4.138	3.348
**2C0P**	[SR3]	10.346	10.713	7.284	12.648	12.732	11.741	11.926	10.232	14.397	14.556
**2C0Q**	[SR3]	10.437	10.891	2.736	12.435	12.681	10.437	10.576	12.984	13.874	13.943
**2GYU**	[SR4]	4.663	4.379	1.743	2.792	3.014	4.679	4.699	1.937	2.804	2.814
**2GYV**	[SR4]	2.991	3.039	1.935	2.934	2.973	3.042	3.037	2.047	2.942	2.763
**2GYW**	[SR4]	2.629	2.693	1.874	7.471	7.536	2.671	2.675	1.963	7.463	7.437
**2H9Y**	[SR5]	4.216	4.378	2.894	6.372	6.449	5.372	5.848	3.342	7.316	7.398
**2HA0**	[SR5]	3.296	3.434	2.220	1.343	1.761	4.327	4.541	3.926	4.378	4.478
**2JEY**	[SR6]	1.939	2.032	2.996	6.391	7.328	1.993	2.146	3.044	6.471	6.484
**2JEZ**	[SR6]	1.937	2.044	2.910	7.341	7.391	1.984	1.989	2.963	7.461	7.473
**2JF0**	[SR6]	2.543	2.633	2.537	4.393	4.551	2.591	2.607	2.668	4.591	4.666
**2JGI**	[SR7]	2.726	2.774	9.373	2.937	3.026	2.831	2.841	10.374	3.116	3.273
**2WHP**	[SR8]	3.039	3.046	1.836	2.591	2.671	2.116	3.598	1.936	2.741	2.794
**2WHQ**	[SR8]	2.791	3.037	1.732	4.397	4.538	2.843	3.868	1.747	4.617	4.882
**2WHR**	[SR8]	2.024	2.071	2.039	2.791	2.937	2.056	2.118	1.993	2.941	2.876
**2WU3**	[SR9]	2.939	3.038	2.883	2.883	2.931	3.049	3.076	2.437	2.995	2.935
**2WU4**	[SR9]	4.326	4.768	2.393	2.973	2.946	4.767	4.794	2.449	3.017	3.176
**3ZLT**	[SR10]	3.394	3.641	2.210	1.393	1.446	4.376	4.222	2.250	2.439	2.556
**3ZLU**	[SR10]	2.719	2.841	2.140	2.439	2.576	3.761	3.814	2.728	2.431	2.316
**3ZLV**	[SR10]	2.764	2.937	2.222	4.325	4.439	3.731	3.941	3.612	2.139	2.261
**4A16**	[SR11]	4.326	4.174	1.821	5.517	5.669	4.438	5.556	2.798	2.249	3.116
**4A23**	[SR12]	4.773	4.934	2.541	3.493	3.561	4.839	4.884	2.674	3.547	3.673
**4ARA**	[SR12]	4.543	4.631	1.559	4.293	4.316	4.661	4.679	1.791	4.397	4.318
**4ARB**	[SR12]	3.637	3.839	1.393	2.553	2.717	3.748	3.791	1.493	2.641	2.661
**4B7Z**	[SR13]	2.092	2.114	3.839	4.393	4.439	2.114	2.174	3.936	4.388	4.397
**4B80**	[SR13]	1.883	1.739	2.946	3.093	3.129	1.984	1.891	3.116	3.273	3.318
**4B81**	[SR13]	2.593	2.542	2.883	3.559	3.776	2.604	2.663	3.731	3.619	3.719
**4B82**	[SR13]	2.938	3.336	4.113	12.931	13.073	2.946	2.944	4.273	12.997	12.874
**4B83**	[SR13]	4.393	4.739	3.096	7.395	7.776	4.484	4.516	3.283	7.436	7.839
**4B84**	[SR13]	2.771	2.895	3.529	6.313	6.416	2.937	2.939	3.661	6.417	6.473
**4B85**	[SR13]	2.636	2.731	3.839	6.419	6.519	2.748	2.816	3.739	6.444	6.393
**4BC0**	[SR14]	3.041	3.077	1.984	2.193	2.293	3.247	3.346	1.996	2.273	2.439
**4BC1**	[SR14]	2.793	2.939	1.739	2.553	2.594	2.811	2.814	1.831	2.617	2.881
**5DTJ**	[SR15]	7.241	7.392	2.935	8.931	8.937	8.321	8.336	3.012	9.241	9.117
**5EHN**	[SR16]	4.436	4.776	7.394	6.931	7.438	4.371	4.384	8.349	6.971	6.593
**5EHQ**	[SR16]	4.363	4.362	6.883	6.771	7.014	4.444	4.472	6.931	6.761	6.831
**5EHZ**	[SR16]	4.373	4.351	6.831	6.931	7.226	4.831	4.829	6.831	6.943	6.933
**5EIA**	[SR16]	7.091	7.116	12.393	6.317	6.339	7.216	7.141	12.449	6.471	6.173
**5EIE**	[SR16]	7.438	7.691	9.837	10.439	10.739	7.519	7.516	10.241	10.558	10.441
**5EIH**	[SR16]	1.737	1.841	2.447	10.536	10.631	1.971	1.988	3.293	10.739	10.432

**5HCU**	[SR15]	1.439	1.553	1.934	10.249	10.555	1.553	1.512	1.974	10.312	10.516
DA		25.55%	22.22%	25.55%	16.67%	13.33%	24.44%	22.22%	32.22%	13.33%	12.22%

[a]Best docked conformation; [b]Best clustered conformation; [c]Rigid docking; [d]F - flexible docking.

Table S10. Structure-based alignment assessment for wild type *Homo sapiens* AChE inhibitors.

Ligand	Ref.	AutoDock		Vina	DOCK6		AutoDock		Vina	DOCK6	
		BDa	BCb		Rc	Fd	BDa	BCb		Rc	Fd
		Experimental Conformation Re-Docking					*Randomized Conformation Re-Docking*				
**2X8B**	[SR17]	5.437	4.974	7.437	1.893	1.764	5.551	5.831	6.393	1.836	1.848
**4BDT**	[SR18]	2.937	2.973	1.697	2.713	2.741	3.491	3.617	1.784	2.973	2.741
**4EY5**	[94]	4.376	4.117	1.761	2.892	2.393	4.372	4.391	1.931	2.931	3.073
**4EY6**	[94]	2.561	2.984	1.936	3.946	3.834	3.072	3.092	2.032	4.032	4.417
**4EY7**	[94]	3.976	3.761	1.774	2.176	2.768	4.894	4.932	1.559	2.813	2.935
**4M0E**	[SR19]	1.773	2.671	1.076	2.076	2.224	1.996	2.081	1.339	1.983	1.996
**4M0F**	[SR19]	2.261	2.558	2.761	1.743	1.839	2.837	2.841	2.547	1.784	1.776
**5FOQ**	[SR20]	2.936	3.041	2.076	3.437	3.338	3.492	3.504	2.841	3.439	3.513
DA		37.5%	18.75%	75.00%	50.00%	50.00%	18.75%	12.50%	68.75%	56.25%	56.25%
		Experimental Conformation Cross-Docking					*Randomized Conformation Cross-Docking*				
**2X8B**	[SR17]	6.838	7.324	4.393	1.949	1.838	5.652	5.559	6.327	2.436	2.389
**4BDT**	[SR18]	2.392	2.224	1.761	2.976	3.721	3.496	3.517	2.937	3.218	3.261
**4EY5**	[94]	4.886	4.596	1.948	3.042	2.984	4.733	4.638	2.996	3.491	3.476
**4EY6**	[94]	2.731	2.837	2.271	1.768	2.072	3.017	2.983	2.746	1.883	1.906
**4EY7**	[94]	3.931	3.915	1.936	1.931	1.836	4.931	4.873	1.582	1.974	1.983
**4M0E**	[SR19]	1.924	2.079	1.948	2.714	2.932	2.041	2.116	1.564	2.892	2.974
**4M0F**	[SR19]	2.736	2.839	2.263	2.046	2.591	2.888	2.913	2.673	2.117	2.217
**5FOQ**	[SR20]	3.498	3.736	2.437	3.966	4.574	3.884	3.941	2.932	3.765	3.964
DA		31.25%	25%	68.75%	56.26%	50%	12.5%	18.75%	56.25%	43.75%	43.75%

[a]Best docked conformation; [b]Best clustered conformation; [c]Rigid docking; [d]F - flexible docking.

Table S11. Binding free energies and individual energy terms of *Mus Musculus* (upper part) and *Homo sapiens* (lower part) AChE in complex with acetylcholine and various targeted pesticides inhibitors.

Complex	ΔE_{ele}[a] (kcal/mol)	ΔE_{vdw}[b] (kcal/mol)	ΔG_{solv}[c] (kcal/mol)	$T\Delta S$[a] (kcal/mol)	ΔG_{bind}[e] (kcal/mol)
**atrazine-*m*AChE**[f]	-41.48±0.62	-51.24±0.91	4.74±0.45	-8.74±0.38	-79.24±0.18
**propazine-*m*AChE**	-52.43±0.47	-50.82±0.35	5.22±0.15	-26.00±0.52	-72.03±0.26
**simazine-*m*AChE**	-53.26±0.41	-50.24±0.63	5.26±0.73	-28.70±0.51	-69.54±0.32
**carbofuran-*m*AChE**	-52.84±0.88	-55.28±0.61	4.37±0.28	-33.32±0.57	-70.43±0.44
**monocrotophos-*m*AChE**	-58.43±0.32	-39.26±0.58	8.24±0.38	-33.99±0.39	-55.46±0.75
**dimethoate-*m*AChE**	-61.24±0.73	-31.36±0.33	9.27±0.27	-30.77±0.22	-52.56±0.43
**carbaryl-*m*AChE**	-51.32±0.38	-49.26±0.68	5.41±0.86	-46.19±0.92	-48.98±0.44
**tebufenozide-*m*AChE**	-41.39±0.41	-39.28±0.19	4.28±0.36	-50.63±0.73	-25.76±0.88
**imidacloprid-*m*AChE**	-38.26±0.78	-51.43±0.28	6.28±0.92	-27.45±0.92	-55.96±0.47
**acetampirid-*m*AChE**	-26.37±0.99	-58.24±0.53	6.32±0.24	-24.93±0.67	-53.36±0.65
**diuron-*m*AChE**	-31.46±0.26	-49.28±0.89	6.03±0.55	-27.87±0.93	-46.84±0.95
**monuron-*m*AChE**	-35.24±0.51	-43.28±0.49	6.33±0.37	-29.66±0.63	-42.53±0.94
**linuron-*m*AChE**	-41.23±0.41	-41.26±0.29	6.05±0.36	-37.50±0.72	-38.94±0.16
**acetylcholine-*m*AChE**	-43.23±0.31	-46.98±0.63	5.28±0.48	-53.48±0.73	-31.45±0.74
**atrazine-*h*AChE**[g]	-53.31±0.62	-41.76±0.91	5.74±0.35	-8.92±0.47	-80.41±0.34
**propazine-*h*AChE**	-58.36±0.47	-47.72±0.35	5.15±0.15	-29.00±0.41	-71.93±0.53
**simazine-*h*AChE**	-57.62±0.74	-51.34±0.41	5.92±0.51	-34.21±0.83	-68.83±0.61
**carbofuran-*h*AChE**	-56.63±0.88	-54.82±0.61	4.95±0.31	-34.56±0.42	-71.94±0.31
**monocrotophos-*h*AChE**	-64.25±0.32	-31.84±0.73	7.89±0.51	-33.38±0.51	-54.82±0.96
**dimethoate-*h*AChE**	-58.43±0.51	-32.72±0.51	8.56±0.46	-28.94±0.68	-53.65±0.93
**carbaryl-*h*AChE**	-53.61±0.63	-48.26±0.22	5.82±0.73	-47.73±0.39	-48.32±0.63
**tebufenozide-*h*AChE**	-40.21±0.37	-42.78±0.22	4.79±0.41	-52.38±0.52	-25.82±0.83
**imidacloprid-*h*AChE**	-47.31±0.83	-50.26±0.45	7.11±0.92	-31.42±0.99	-59.04±0.73
**acetampirid-*h*AChE**	-24.52±0.83	-58.73±0.26	6.57±0.41	-22.70±0.42	-53.98±0.68
**diuron-*h*AChE**	-29.37±0.72	-50.74±0.51	6.17±0.42	-27.72±0.93	-46.22±0.92
**monuron-*h*AChE**	-36.73±0.41	-44.17±0.28	6.54±0.81	-31.24±0.73	-43.12±0.77
**linuron-*h*AChE**	-41.78±0.41	-42.03±0.61	6.52±0.61	-37.57±0.74	-39.72±0.53
**acetylcholine-*h*AChE**	-42.42±0.62	-48.35±0.22	5.41±0.83	-52.69±0.82	-32.67±0.31

[a]Electrostatic energy difference; [b]van der Waals energy difference; [c]Solvation free energy; [d]The entropy change; [e]Predicted binding free energy; [f]*Mus musculus* AChE; [g]*Homo sapiens* AChE.

Table S12. Linear regression parameters for QSAR model 5 (acute toxicity against *Mus musculus*).

Compound	LD_{50} (mg/kg)	MW	$\Delta G_{binding}$ (kcal/mol)	Exp. pLD_{50}	Fitted pLD_{50}
**atrazine**	0.85	215.69	-9.50	5.40	5.05
**propazin**	3.18	229.71	-9.20	4.86	4.81
**simazine**	5	201.66	-8.70	4.61	4.42
**carbofuran**	2	221.11	-8.70	5.04	4.42
**monocrotophos**	14	223.16	-8.40	4.20	4.18
**dimethoate**	60	229.25	-8.20	3.58	4.02
**carbaryl**	100	201.22	-6.10	3.30	2.37
**tebufenozide**	5000	352.48	-7.50	1.85	3.47
**imidacloprid**	131	269.69	-7.80	3.31	3.71
**acetamiprid**	184	221.69	-8.10	3.08	-2.44
**diuron**	500	233.09	-6.50	2.67	2.68
**monuron**	1700	198.65	-6.20	2.07	2.45
**linuron**	2400	249.09	-5.30	2.02	1.74

Table S13. External validation of QSAR model 5 (acute toxicity against *Mus musculus*).

Compound	LD_{50} (mg/kg)	MW	$\Delta G_{binding}$ (kcal/mol)	Exp. pLD_{50}	Fitted pLD_{50}
**azamethiphos**	1040.00	324.68	-8.10	4.07	3.95
**azinphos-methyl**	7.00	317.32	-7.80	6.25	3.71
**chlorpyrifos**	2000.00	350.59	-7.60	3.79	3.55
**DDVP**	17.00	220.98	-5.30	5.86	1.74
**diazinon**	66.00	304.35	-8.00	5.27	3.87
**fenitrothion**	500.00	277.23	-7.70	4.39	3.63
**glyphosate**	5000.00	169.07	-4.90	3.39	1.42
**malathion**	290.00	330.36	-6.80	4.63	2.92
**methyl parathion**	18.00	263.21	-7.20	5.84	3.24
**naled (dibrom)**	160.00	380.78	-5.70	4.89	2.05
**parathion**	2.00	291.26	-7.00	6.79	3.08
**phosmet**	113.00	317.32	-7.80	5.04	3.71
**TCVP**	465.00	365.96	-8.30	4.42	4.10
**terbufos**	1.60	288.43	-5.20	6.89	1.66
**methiocarb**	350.00	225.31	-7.00	4.55	3.08
**methomyl**	12.00	162.21	-5.30	6.01	1.74
**oxamyl**	5.40	219.26	-6.40	6.36	2.61
**DDT**	113.00	354.49	-9.10	5.04	4.74
**2,4-D**	639.00	221.04	-7.00	4.29	3.08
**dicamba**	757.00	221.04	-6.90	4.21	3.00
**DEET**	1.95	191.27	-7.80	6.80	3.71
**sulfoxaflor**	750.00	277.27	-8.30	4.22	4.10

Table S14. Linear regression parameters for QSAR model 5 (acute toxicity against *Homo sapiens*).

Compound	LD_{50} (mg/kg)	MW	$\Delta G_{binding}$ (kcal/mol)	Calculated pLD_{50}	Fitted pLD_{50}
**atrazine**	0.07	215.69	-10.10	6.50	5.52
**propazin**	0.26	229.71	-9.70	5.95	5.21
**simazine**	0.41	201.66	-9.10	5.70	4.74
**carbofuran**	0.16	221.11	-9.60	6.14	5.13
**monocrotophos**	1.14	223.16	-8.60	5.29	4.34
**dimethoate**	4.86	229.25	-8.30	4.67	4.10
**carbaryl**	8.11	201.22	-7.80	4.39	3.71
**tebufenozide**	405.40	352.48	-5.60	2.94	1.98
**imidacloprid**	10.62	269.69	-8.10	4.40	3.95
**acetamiprid**	14.92	221.69	-7.70	4.17	3.63
**diuron**	40.54	233.09	-6.90	3.76	3.00
**monuron**	137.84	198.65	-6.70	3.16	2.84
**linuron**	194.59	249.09	-5.90	3.11	2.21

Table S15. External validation of QSAR model 5 (acute toxicity against *Homo sapiens*).

Compound	LD_{50} (mg/kg)	MW	$\Delta G_{binding}$ (kcal/mol)	Calculated pLD_{50}	Fitted pLD_{50}
**azamethiphos**	1040.00	324.68	-8.20	2.49	4.03
**azinphos-methyl**	7.00	317.32	-8.00	4.66	3.87
**chlorpyrifos**	2000.00	350.59	-8.10	2.24	3.95
**DDVP**	17.00	220.98	-5.40	4.11	1.82
**diazinon**	66.00	304.35	-7.80	3.66	3.71
**fenitrothion**	500.00	277.23	-7.90	2.74	3.79
**glyphosate**	5000.00	169.07	-4.90	1.53	1.42
**malathion**	290.00	330.36	-6.30	3.06	2.53
**methyl parathion**	18.00	263.21	-7.50	4.17	3.47
**naled (dibrom)**	160.00	380.78	-5.70	3.38	2.05
**parathion**	2.00	291.26	-7.90	5.16	3.79
**phosmet**	113.00	317.32	-8.20	3.45	4.03
**TCVP**	465.00	365.96	-8.00	2.90	3.87
**terbufos**	1.60	288.43	-4.90	5.26	1.42
**methiocarb**	350.00	225.31	-7.80	2.81	3.71
**methomyl**	12.00	162.21	-5.40	4.13	1.82
**oxamyl**	5.40	219.26	-6.30	4.61	2.53
**DDT**	113.00	354.49	-9.20	3.50	4.81
**2,4-D**	639.00	221.04	-7.50	2.54	3.47
**dicamba**	757.00	221.04	-7.30	2.47	3.32
**DEET**	1.95	191.27	-7.90	4.99	3.79
**sulfoxaflor**	750.00	277.27	-8.70	2.57	4.42

Supplementary material references (SRs)

SR1. Bourne, Y.; Taylor, P.; Radić, Z.; Marchot, P. Structural insights into ligand interactions at the acetylcholinesterase Peripheral Anionic Site. *EMBO J* **2003**, *22*, 1–12. DOI: 10.1093/emboj/cdg005

SR2. Bourne, Y.; Kolb, H.C.; Radić, Z.; Sharpless, K.B.; Taylor, P.; Marchot, P. Freeze-frame inhibitor captures acetylcholinesterase in a unique conformation. *Proc Natl Acad Sci USA* **2004**, *101*, 1449–1454. DOI: 10.1073/pnas.0308206100

SR3. Ekstrom, F.; Akfur, C.; Tunemalm, A.-K.; Lundberg, S. Structural changes of phenylalanine 338 and histidine 447 revealed by the crystal structures of tabun-inhibited murine acetylcholinesterase. *Biochemistry* **2006**, *45*, 74–81. DOI: 10.1021/bi051286t

SR4. Ekstrom, F.; Pang, Y.P.; Boman, M.; Artursson, E.; Akfur, C.; Borjegren, S. Crystal structures of acetylcholinesterase in complex with HI-6, ortho-7 and obidoxime: Structural basis for differences in the ability to reactivate tabun conjugates. *Biochem Pharmacol* **2006**, *72*, 597–607. DOI: 10.1016/j.bcp.2006.05.027

SR5. Bourne, Y.; Radić, Z.; SulzenbAChEr, G.; Kim, E.; Taylor, P.; Marchot, P. Substrate and product trafficking through the active center gorge of acetylcholinesterase analyzed by crystallography and equilibrium binding. *J Biol Chem* **2006**, *281*, 29256–29267. DOI: 10.1074/jbc.M603018200

SR6. Ekstrom, F.J.; Astot, C.; Pang, Y.P. Novel nerve-agent antidote design based on crystallographic and mass spectrometric analyses of tabun-conjugated acetylcholinesterase in complex with antidotes. *Clin Pharmacol Ther* **2007**, *82*, 282–293. DOI: 10.1038/sj.clpt.6100151

SR7. Hornberg, A.; Tunemalm, A.K.; Ekstrom, F. Crystal structures of acetylcholinesterase in complex with organophosphorus compounds suggest that the acyl pocket modulates the aging reaction by precluding the formation of the trigonal bipyramidal transition state. *Biochemistry* **2007**, *46*, 4815–4825. DOI: 10.1021/bi0621361

SR8. Ekstrom, F.; Hornberg, A.; Artursson, E.; Hammarstrom, L.G.; Schneider, G.; Pang, Y.P. Structure of HI-6•sarin-acetylcholinesterase determined by x-ray crystallography and molecular dynamics simulation: Reactivator mechanism and design. *Plos One* **2009**, *4*, e5957 1–19. DOI: 10.1371/journal.pone.0005957

SR9. Hornberg, A.; Artursson, E.; Warme, R.; Pang, Y-P.; Ekstrom, F. Crystal structures of oxime-bound fenamiphos-acetylcholinesterases: Reactivation involving flipping of the His447 ring to form a reactive Glu334-His447-oxime triad. *Biochem Pharmacol* **2010**, *79*, 507–515. DOI: 10.1016/j.bcp.2009.08.027

SR10. Artursson, E.; Andersson, P.O.; Akfur, C.; Linusson, A.; Borjegren, S.; Ekstrom, F. Catalytic-site conformational equilibrium in nerve-agent adducts of acetylcholinesterase: Possible implications for the HI-6 antidote substrate specificity. *Biochem Pharmacol* **2013**, *85*, 1389–1397. DOI: 10.1016/j.bcp.2013.01.016

SR11. Ronco, C.; Carletti, E.; Colletier, J-P.; Weik, M.; Nachon, F.; Jean, L.; Renard, P-Y. Huprine derivatives as sub-nanomolar human acetylcholinesterase inhibitors: From rational design to validation by x-ray crystallography. *ChemMedChem* **2012**, *7*, 400–405. DOI: 10.1002/cmdc.201100438

SR12. Berg, L.; Andersson, C.D.; Artursson, E.; Hornberg, A.; Tunemalm, A-K.; Linusson, A.; Ekstrom, F. Targeting acetylcholinesterase: Identification of chemical leads by high throughput screening,

structure determination and molecular modelling. *Plos One* **2011**, *6*, e26039 1–12. DOI: 10.1371/journal.pone.0026039

SR13. Andersson, C.D.; Forsgren, N.; Akfur, C.; Allgardsson, A.; Berg, L.; Engdahl, C.; Qian, W.; Ekstrom, F.; Linusson, A. Divergent structure-activity relationships of structurally similar acetylcholinesterase inhibitors. *J Med Chem* **2013**, *56*, 7615–7624. DOI: 10.1021/jm400990p

SR14. Carletti, E.; Colletier, J-P.; Schopfer, L.M.; Santoni, G.; Masson, P.; Lockridge, O.; Nachon, F.; Weik, M. Inhibition pathways of the potent organophosphate CBDP with cholinesterases revealed by x-ray crystallographic snapshots and mass spectrometry. *Chem Res Toxicol* **2013**, *26*, 280–289. DOI: 10.1021/tx3004505

SR15. Katz, F.S.; Pecic, S.; Tran, T.H.; Trakht, I.; Schneider, L.; Zhu, Z.; Ton-That, L.; Luzac, M.; Zlatanic, V.; Damera, S.; Macdonald, J.; Landry, D.W.; Tong, L.; Stojanovic, M.N. Discovery of new classes of compounds that reactivate acetylcholinesterase inhibited by organophosphates. *ChemBioChem* **2015**, *16*, 2205–2215. DOI: 10.1002/cbic.201500348

SR16. Bourne, Y.; Sharpless, K.B.; Taylor, P.; Marchot, P. Steric and dynamic parameters influencing *in situ* cycloadditions to form triazole inhibitors with crystalline acetylcholinesterase. *J Am Chem Soc* **2016**, *138*, 1611–1621. DOI: 10.1021/jacs.5b11384

SR17. Carletti, E.; Colletier, J-P.; Dupeux, F.; Trovaslet, M.; Masson, P.; Nachon, F. Structural evidence that human acetylcholinesterase inhibited by tabun ages through o-dealkylation. *J Med Chem* **2010**, *53*, 4002–4008. DOI: 10.1021/jm901853b

SR18. Nachon, F.; Carletti, E.; Ronco, C.; Trovaslet, M.; Nicolet, Y.; Jean, L.; Renard, P.Y. Crystal structures of human cholinesterases in complex with huprine w and tacrine: Elements of specificity for anti-Alzheimer's drugs targeting acetyl- and butyryl-cholinesterase. *Biochem J* **2013**, *453*, 393–399. DOI: 10.1042/BJ20130013

SR19. Cheung, J.; Gary, E.N.; Shiomi, K.; Rosenberry, T.L. Structures of human acetylcholinesterase bound to dihydrotanshinone i and territrem b show peripheral site flexibility. *ACS Med Chem Lett* **2013**, *4*, 1091–1096. DOI: 10.1021/ml400304w

SR20. Berg, L.; Mishra, B.K.; Andersson, C.D.; Ekstrom, F.; Linusson, A. The nature of activated non-classical hydrogen bonds: A case study on acetylcholinesterase-ligand complexes. *Chemistry* **2016**, *22*, 2672–2681. DOI: 10.1002/chem.201503973
